# Supplementary material for: Global and regional burden estimation of HIV-associated non-Hodgkin’s lymphoma: a meta-analysis and modelling analysis protocol
Source: BMJ Open. 2024 Jun 25;14(6):e075933. doi: 10.1136/bmjopen-2023-075933 (PMC11210503; doi:10.1136/bmjopen-2023-075933)
Supplement: Supplementary data [file bmjopen-2023-075933supp001.pdf]

Appendix

Appendix 1. Electronic databases search strategies in PubMed, Embase, Cochrane Library and Web of Science

PubMed

Set	Search	Results
#1	"lymphoma, non hodgkin"[MeSH Terms]	
#2	"hiv"[Title/Abstract] OR "hiv-1"[Title/Abstract] OR "hiv 1"[Title/Abstract] OR "hiv 2"[Title/Abstract] OR "hiv-2"[Title/Abstract] OR "human immunodeficiency virus"[Title/Abstract] OR "human immuno deficiency virus"[Title/Abstract] OR "human immunedeficiency virus"[Title/Abstract] OR "human immune deficiency virus"[Title/Abstract] OR "acquired immunodeficiency syndrome"[MeSH Terms] OR "acquired immunodeficiency syndrome"[Title/Abstract] OR "acquired immuno-deficiency syndrome"[Title/Abstract]OR "acquired immunedeficiency syndrome"[Title/Abstract]	
#3	"lymphoma non hodgkin"[Title/Abstract] OR (("Lymphoma"[MeSH Terms] OR "Lymphoma"[All Fields] OR "Lymphomas"[All Fields] OR "lymphoma s"[All Fields]) AND "small cleaved cell diffuse"[Title/Abstract]) OR ((("Lymphoma"[MeSH Terms] OR "Lymphoma"[All Fields] OR "Lymphomas"[All Fields] OR "lymphoma s"[All Fields]) AND ("atypic"[All Fields] OR "atypical"[All Fields] OR "atypicalities"[All Fields] OR "atypicality"[All Fields] OR "atypically"[All Fields] OR "atypicals"[All Fields] OR "atypism"[All Fields] OR "atypisms"[All Fields]) AND ("diffusable"[All Fields] OR "diffusant"[All Fields] OR "diffusants"[All Fields] OR "Diffuse"[All Fields] OR "diffusely"[All Fields] OR "diffuses"[All Fields] OR "diffusibility"[All Fields] OR "diffusible"[All Fields] OR "diffusion"[MeSH Terms] OR "diffusion"[All Fields] OR "diffused"[All Fields] OR "diffusing"[All Fields] OR "diffusions"[All Fields] OR "diffusive"[All Fields] OR "diffusively"[All Fields] OR "diffusivities"[All Fields] OR "diffusivity"[All Fields])) AND "small lymphoid"[Title/Abstract]) OR "diffuse small cleaved cell lymphoma"[Title/Abstract] OR "diffuse small cleaved cell lymphoma"[Title/Abstract] OR (("Lymphoma"[MeSH Terms] OR "Lymphoma"[All Fields] OR "Lymphomas"[All Fields] OR "lymphoma s"[All Fields]) AND "small cleaved cell diffuse"[Title/Abstract]) OR (("Lymphoma"[MeSH Terms] OR "Lymphoma"[All Fields] OR "Lymphomas"[All Fields] OR "lymphoma s"[All Fields]) AND "Nonhodgkins"[Title/Abstract]) OR "nonhodgkins lymphoma"[Title/Abstract] OR "lymphoma non hodgkins"[Title/Abstract] OR "lymphoma non hodgkins"[Title/Abstract] OR "non hodgkins lymphoma"[Title/Abstract] OR "non hodgkin lymphoma"[Title/Abstract] OR "non hodgkin lymphoma"[Title/Abstract] OR "lymphoma nonhodgkin s"[Title/Abstract] OR	

	"lymphoma nonhodgkin"[Title/Abstract] OR "small cleaved cell lymphoma diffuse"[Title/Abstract] OR "small cleaved cell lymphoma diffuse"[Title/Abstract] OR "lymphoma non hodgkin s"[Title/Abstract] OR "lymphoma non hodgkin s"[Title/Abstract] OR "lymphoma diffuse"[Title/Abstract] OR "diffuse lymphoma"[Title/Abstract] OR "diffuse lymphomas"[Title/Abstract] OR "sarcoma lymphatic"[Title/Abstract] OR "Lymphosarcoma"[Title/Abstract] OR "Lymphosarcomas"[Title/Abstract] OR "lymphatic sarcoma"[Title/Abstract] OR "lymphatic sarcomas"[Title/Abstract] OR "lymphoma low grade"[Title/Abstract] OR "low grade lymphoma"[Title/Abstract] OR "low grade lymphomas"[Title/Abstract] OR "lymphoma low grade"[Title/Abstract] OR "lymphoma mixed cell"[Title/Abstract] OR "lymphoma mixed cell"[Title/Abstract] OR "mixed cell lymphoma"[Title/Abstract] OR "mixed cell lymphoma"[Title/Abstract] OR "mixed cell lymphomas"[Title/Abstract] OR "lymphoma mixed"[Title/Abstract] OR "mixed lymphoma"[Title/Abstract] OR "mixed lymphomas"[Title/Abstract] OR "lymphoma mixed lymphocytic histiocytic"[Title/Abstract] OR ("Lymphocytic-Histiocytic"[All Fields] AND "lymphoma mixed"[Title/Abstract]) OR ("Lymphocytic-Histiocytic"[All Fields] AND "lymphomas mixed"[Title/Abstract]) OR "lymphoma mixed lymphocytic histiocytic"[Title/Abstract] OR "mixed lymphocytic histiocytic lymphoma"[Title/Abstract] OR "mixed lymphocytic histiocytic lymphomas"[Title/Abstract] OR (("lymphoma, non hodgkin"[MeSH Terms] OR ("Lymphoma"[All Fields] AND "Non-Hodgkin"[All Fields]) OR "non-hodgkin lymphoma"[All Fields] OR ("Lymphoma"[All Fields] AND "Non"[All Fields] AND "Hodgkin"[All Fields]) OR "lymphoma non hodgkin"[All Fields]) AND "Familial"[Title/Abstract]) OR "lymphoma small noncleaved cell"[Title/Abstract] OR "lymphoma small noncleaved cell"[Title/Abstract] OR "small noncleaved cell lymphoma"[Title/Abstract] OR ("Noncleaved-Cell"[All Fields] AND "lymphoma small"[Title/Abstract]) OR "small noncleaved cell lymphoma"[Title/Abstract] OR "small noncleaved cell lymphomas"[Title/Abstract] OR "diffuse undifferentiated lymphoma"[Title/Abstract] OR "diffuse undifferentiated lymphomas"[Title/Abstract] OR "lymphoma diffuse undifferentiated"[Title/Abstract] OR "undifferentiated lymphoma diffuse"[Title/Abstract] OR "lymphoma small non cleaved cell"[Title/Abstract] OR "lymphoma small non cleaved cell"[Title/Abstract] OR ("Non-Cleaved-Cell"[All Fields] AND "lymphoma small"[Title/Abstract]) OR "small non cleaved cell lymphomas"[Title/Abstract] OR "small non cleaved cell lymphoma"[Title/Abstract] OR "small non cleaved cell lymphoma"[Title/Abstract] OR (("Lymphoma"[MeSH Terms] OR "Lymphoma"[All Fields] OR "Lymphomas"[All Fields] OR "lymphoma s"[All Fields]) AND "undifferentiated diffuse"[Title/Abstract]) OR "lymphoma intermediate grade"[Title/Abstract] OR "intermediate grade lymphoma"[Title/Abstract] OR "intermediate grade lymphomas"[Title/Abstract]	

	OR "lymphoma intermediate grade"[Title/Abstract] OR "lymphoma undifferentiated"[Title/Abstract] OR "lymphoma pleomorphic"[Title/Abstract] OR "pleomorphic lymphoma"[Title/Abstract] OR "diffuse mixed cell lymphomas"[Title/Abstract] OR "diffuse mixed cell lymphoma"[Title/Abstract] OR "diffuse mixed cell lymphoma"[Title/Abstract] OR "non hodgkin s lymphoma"[Title/Abstract] OR "non hodgkin s lymphoma"[Title/Abstract] OR "nonhodgkin s lymphoma"[Title/Abstract] OR ("diffuse mixed small"[Title/Abstract] AND "large cell lymphoma"[Title/Abstract]) OR (("Mixed"[All Fields] OR "mixes"[All Fields] OR "mixing"[All Fields] OR "mixings"[All Fields]) AND "cell lymphoma diffuse"[Title/Abstract]) OR ("Mixed-Cell"[All Fields] AND "lymphoma diffuse"[Title/Abstract]) OR (("Lymphoma"[MeSH Terms] OR "Lymphoma"[All Fields] OR "Lymphomas"[All Fields] OR "lymphoma s"[All Fields]) AND "mixed cell diffuse"[Title/Abstract]) OR ("lymphoma small"[Title/Abstract] AND ((("Large"[All Fields] OR "largely"[All Fields] OR "larges"[All Fields]) AND "Cleaved-Cell"[All Fields]) AND "Diffuse"[Title/Abstract])) OR (("lymphoma, non hodgkin"[MeSH Terms] OR ("Lymphoma"[All Fields] AND "Non-Hodgkin"[All Fields]) OR "non-hodgkin lymphoma"[All Fields] OR ("Lymphoma"[All Fields] AND "Diffuse"[All Fields]) OR "lymphoma diffuse"[All Fields]) AND "mixed lymphocytic histiocytic"[Title/Abstract]) OR ("lymphoma mixed small"[Title/Abstract] AND "large cell diffuse"[Title/Abstract]) OR ("mixed small"[Title/Abstract] AND "large cell lymphoma diffuse"[Title/Abstract]) OR (("Lymphoma"[MeSH Terms] OR "Lymphoma"[All Fields] OR "Lymphomas"[All Fields] OR "lymphoma s"[All Fields]) AND "mixed cell diffuse"[Title/Abstract]) OR "lymphoma high grade"[Title/Abstract] OR "high grade lymphomas"[Title/Abstract] OR "high grade lymphoma"[Title/Abstract] OR "lymphoma high grade"[Title/Abstract] OR "Reticulosarcomas"[Title/Abstract] OR "Reticulosarcoma"[Title/Abstract] OR "reticulum cell sarcomas"[Title/Abstract] OR "reticulum cell sarcoma"[Title/Abstract] OR "sarcoma reticulum cell"[Title/Abstract] OR "sarcoma reticulum cell"[Title/Abstract] OR "reticulum cell sarcomas"[Title/Abstract] OR "reticulum cell sarcoma"[Title/Abstract] OR "undifferentiated lymphomas"[Title/Abstract] OR "undifferentiated lymphoma"[Title/Abstract] OR "pleomorphic lymphomas"[Title/Abstract]	
#4	"prevalence"[Text Word] OR "incidence"[Text Word] OR "epidemiol*"[Text Word] OR "survey"[Text Word] OR "rapid assessment"[Text Word] OR "situation assessment"[Text Word] OR "situational assessment"[Text Word] OR "rar"[Text Word] OR "cohort"[Text Word] OR "surveillance"[Text Word] OR "seroprevalence"[Text Word] OR "seroincidence"[Text Word] OR "screening"[Text Word] OR "mortality"[Title/Abstract] OR "mortalities"[Title/Abstract] OR "mortal"[Title/Abstract] OR "dead"[Title/Abstract] OR "death"[Title/Abstract] OR "deaths"[Title/Abstract] OR "decease*"[Title/Abstract] OR "demise*"[Title/Abstract] OR "die"[Title/Abstract] OR "died"[Title/Abstract] OR "dies"[Title/Abstract] OR	

	"dying"[Title/Abstract] OR "fatal"[Title/Abstract] OR "fatality"[Title/Abstract] OR "fatalities"[Title/Abstract] OR "nonsurvival*"[Title/Abstract] OR "nonsurvival*"[Title/Abstract] OR "survival*"[Title/Abstract] OR "lethality"[Title/Abstract] OR "epidemiologic methods"[MeSH Major Topic] OR "epidemiologic studies"[MeSH Major Topic] OR "sentinel surveillance"[MeSH Major Topic] OR "seroepidemiologic studies"[MeSH Major Topic] OR "cohort studies"[MeSH Major Topic] OR "longitudinal studies"[MeSH Major Topic] OR "follow up studies"[MeSH Major Topic] OR "prospective studies"[MeSH Major Topic] OR "diagnostic services"[MeSH Major Topic] OR "mass screening"[MeSH Major Topic] OR "mortality"[MeSH Major Topic] OR "hospital mortality"[MeSH Major Topic] OR "death"[MeSH Major Topic] OR "cause of death"[MeSH Major Topic] OR "death certificates"[MeSH Major Topic] OR "fatal outcome"[MeSH Major Topic] OR "autopsy"[MeSH Major Topic] OR "survival rate"[MeSH Major Topic]	
#5	#1 OR #2	
#6	#3 and #4 and #5	

Embase

Set	Search	Results
#1	'human immunodeficiency virus infection'/exp OR 'human immunodeficiency virus'/exp OR 'hiv survivor'/exp OR hiv:ti,ab,kw OR 'hiv-1':ti,ab,kw OR 'hiv-2':ti,ab,kw OR 'human immunodeficiency virus':ti,ab,kw OR 'human immunodeficiency':ti,ab,kw OR 'human immunedeficiency virus':ti,ab,kw OR 'human immune deficiency virus':ti,ab,kw OR 'human immune-deficiency virus':ti,ab,kw OR 'acquired immune-deficiency syndrome':ti,ab,kw OR 'acquired immunedeficiency syndrome':ti,ab,kw OR 'acquired immunodeficiency syndrome':ti,ab,kw OR 'acquired immuno-deficiency syndrome':ti,ab,kw	
#2	prevalence:ti,ab,de OR incidence:ti,ab,de OR epidemiol*:ti,ab,de OR survey:ti,ab,de OR 'rapid assessment':ti,ab,de OR 'situation assessment':ti,ab,de OR 'situational assessment':ti,ab,de OR rar:ti,ab,de OR cohort:ti,ab,de OR surveillance:ti,ab,de OR seroprevalence:ti,ab,de OR seroincidence:ti,ab,de OR seroepidemiol*:ti,ab,de OR screening:ti,ab,de OR 'mass screening':ti,ab,de OR mortality:ti,ab OR mortalities:ti,ab OR mortal:ti,ab OR dead:ti,ab OR death:ti,ab OR deaths:ti,ab OR decease*:ti,ab OR demise*:ti,ab OR die:ti,ab OR died:ti,ab OR dies:ti,ab OR dying:ti,ab OR fatal:ti,ab OR fatality:ti,ab OR fatalities:ti,ab OR 'non survival*':ti,ab OR nonsurvival*:ti,ab OR survival*:ti,ab OR lethality:ti,ab OR epidemiology:de OR 'cancer epidemiology'/exp OR 'cancer statistics'/exp OR 'disease surveillance'/exp OR 'health survey'/exp OR 'incidence'/exp OR 'infection rate'/exp OR 'prevelance' OR 'sentinel surveillance'/exp OR 'seroepidemiology'/exp OR 'cohort analysis'/exp OR 'longitudinal study'/exp OR 'prospective study'/exp OR 'preventive health service'/exp OR 'mass	

	screening'/exp OR 'cancer mortality'/exp OR 'all cause mortality'/exp OR 'mortality':de OR 'hospital mortality'/exp OR 'mortality rate'/exp OR 'death'/exp OR 'cause of death'/exp OR 'dying'/exp OR 'fatality'/exp OR 'lethality'/exp OR 'death certificate'/exp OR 'autopsy'/exp OR 'cancer survival'/exp OR 'survival rate'/exp	
#3	'non-hodgkin lymphoma'/exp	
#4	'lymphoma, non hodgkin':ab,ti	
#5	'lymphoma, small cleaved-cell, diffuse':ab,ti	
#6	'lymphoma, atypical diffuse small lymphoid':ab,ti	
#7	'diffuse small cleaved-cell lymphoma':ab,ti	
#8	'diffuse small cleaved cell lymphoma':ab,ti	
#9	'lymphoma, small cleaved cell, diffuse':ab,ti	
#10	'lymphoma, nonhodgkins':ab,ti	
#11	'nonhodgkins lymphoma':ab,ti	
#12	'lymphoma, non-hodgkins':ab,ti	
#13	'lymphoma, non hodgkins':ab,ti	
#14	'lymphoma, non hodgkins':ab,ti	
#15	'non-hodgkins lymphoma':ab,ti	
#16	'non-hodgkin lymphoma':ab,ti	
#17	'lymphoma, nonhodgkin':ab,ti	
#18	'small cleaved-cell lymphoma, diffuse':ab,ti	
#19	'lymphoma, diffuse':ab,ti	
#20	'ldiffuse lymphomae':ab,ti	
#21	'diffuse lymphomae':ab,ti	
#22	'diffuse lymphomas':ab,ti	
#23	'diffuse lymphoma':ab,ti	
#24	'sarcoma, lymphatic':ab,ti	
#25	'lymphosarcoma':ab,ti	
#26	'lymphosarcomas':ab,ti	
#27	'llymphatic sarcomas':ab,ti	
#28	'lymphatic sarcomas':ab,ti	
#29	'lymphatic sarcomas':ab,ti	
#30	'lymphoma, low-grade':ab,ti	
#31	'low-grade lymphoma':ab,ti	
#32	'low-grade lymphomas':ab,ti	
#33	'lymphoma, low grade':ab,ti	
#34	'lymphoma, mixed-cell':ab,ti	
#35	'lymphoma, mixed cell':ab,ti	
#36	'mixed-cell lymphoma':ab,ti	
#37	'mixed cell lymphoma':ab,ti	
#38	'mixed-cell lymphomas':ab,ti	
#39	'lymphoma, mixed':ab,ti	
#40	'mixed lymphoma':ab,ti	

#41	'lymphoma, mixed lymphocytic-histiocytic':ab,ti	
#42	'lymphocytic-histiocytic lymphoma, mixed':ab,ti	
#43	'mixed lymphocytic-histiocytic lymphoma':ab,ti	
#44	'mixed lymphocytic-histiocytic lymphomas':ab,ti	
#45	'lymphoma, non-hodgkin, familial':ab,ti	
#46	'lymphoma, small noncleaved-cell':ab,ti	
#47	'lymphoma, small noncleaved cell':ab,ti	
#48	'small noncleaved-cell lymphoma':ab,ti	
#49	'noncleaved-cell lymphoma, small':ab,ti	
#50	'small noncleaved cell lymphoma':ab,ti	
#51	'small noncleaved-cell lymphomas':ab,ti	
#52	'diffuse undifferentiated lymphoma':ab,ti	
#53	'diffuse undifferentiated lymphomas':ab,ti	
#54	#3 OR #4 OR #5 OR #6 OR #7 OR #8 OR #9 OR #10 OR #11 OR #12 OR #13 OR #14 OR #15 OR #16 OR #17 OR #18 OR #19 OR #20 OR #21 OR #22 OR #23 OR #24 OR #25 OR #26 OR #27 OR #28 OR #29 OR #30 OR #31 OR #32 OR #33 OR #34 OR #35 OR #36 OR #37 OR #38 OR #39 OR #40 OR #41 OR #42 OR #43 OR #44 OR #45 OR #46 OR #47 OR #48 OR #49 OR #50 OR #51 OR #52 OR #53	
#55	#1 AND #2 AND #54	

Cochrane Library

Set	Search	Results
#1	MeSH descriptor: [HIV] explode all trees	
#2	(hiv):ti,ab,kw (Word variations have been searched)	
#3	#1 OR #2	
#4	("non-Hodgkin lymphoma"):ti,ab,kw (Word variations have been searched)	
#5	MeSH descriptor: [Lymphoma, Non-Hodgkin] explode all trees	
#6	#4 OR #5	
#7	#6 OR #3	

Web of Science

Set	Search	Results
#1	"TS=(prevalence OR incidence OR epidemiol* OR survey OR ""rapid assessment"" OR ""situation assessment"" OR ""situational assessment"" OR rar OR cohort OR surveillance OR seroprevalence OR seroincidence OR seroepidemiol* OR screening OR mortality OR mortalities OR mortal OR dead OR death OR deaths OR decease* OR demise* OR die OR died OR dies OR dying OR fatal OR fatality OR fatalities OR non-survival* OR Nonsurvival* OR survival* OR lethality OR ""longitudinal studies"" OR ""follow-up studies"" OR ""prospective studies"" OR ""Mass Screening"" OR	

	""Autopsy"")	
#2	"TS=(“hiv” OR “hiv-1” OR “hiv-2” OR “hiv1” OR “hiv2” OR hiv infect* OR “human immunodeficiency virus” OR “human immunedeficiency virus” OR “human immuno-deficiency virus” OR “human immune-deficiency virus” OR ((human immun*) AND (“deficiency virus”)) OR “acquired immunodeficiency syndrome” OR “acquired immunedeficiency syndrome” OR “acquired immuno-deficiency syndrome” OR “acquired immune-deficiency syndrome” OR ((acquired immun*) AND (“deficiency syndrome”)))	
#3	"TS=(“Non-Hodgkin lymphoma” OR “Lymphoma, Non Hodgkin” OR “Lymphoma, Small Cleaved-Cell, Diffuse” OR “Lymphoma, Atypical Diffuse Small Lymphoid” OR “Diffuse Small Cleaved-Cell Lymphoma” OR “Diffuse Small Cleaved Cell Lymphoma” OR “Lymphoma, Small Cleaved Cell, Diffuse” OR “Lymphoma, Nonhodgkins” OR “Nonhodgkins Lymphoma” OR “Lymphoma, Non-Hodgkins” OR “Lymphoma, Non Hodgkins” OR “Non-Hodgkins Lymphoma” OR “Non-Hodgkin Lymphoma” OR “Non Hodgkin Lymphoma” OR “Lymphoma, Nonhodgkin's” OR “Lymphoma, Nonhodgkin” OR “Small Cleaved-Cell Lymphoma, Diffuse” OR “Small Cleaved Cell Lymphoma, Diffuse” OR “Lymphoma, Non-Hodgkin's” OR “Lymphoma, Non Hodgkin's” OR “Lymphoma, Diffuse” OR “Diffuse Lymphoma” OR “Diffuse Lymphomas” OR “Sarcoma, Lymphatic” OR “Lymphosarcoma” OR “Lymphosarcomas” OR “Lymphatic Sarcoma” OR “Lymphatic Sarcomas” OR “Lymphoma, Low-Grade” OR “Low-Grade Lymphoma” OR “Low-Grade Lymphomas” OR “Lymphoma, Low Grade” OR “Lymphoma, Mixed-Cell” OR “Lymphoma, Mixed Cell” OR “Mixed-Cell Lymphoma” OR “Mixed Cell Lymphoma” OR “Mixed-Cell Lymphomas” OR “Lymphoma, Mixed” OR “Mixed Lymphoma” OR “Mixed Lymphomas” OR “Lymphoma, Mixed Lymphocytic-Histiocytic”OR “Lymphocytic-Histiocytic Lymphoma, Mixed” OR “Lymphocytic-Histiocytic Lymphomas, Mixed” OR “Lymphoma, Mixed Lymphocytic Histiocytic” OR “Mixed Lymphocytic-Histiocytic Lymphoma” OR “Mixed Lymphocytic-Histiocytic Lymphomas” OR “Lymphoma, Non-Hodgkin, Familial” OR “Lymphoma, Small Noncleaved-Cell” OR “Lymphoma, Small Noncleaved Cell” OR “Small Noncleaved-Cell Lymphoma” OR “Noncleaved-Cell Lymphoma, Small” OR “Small Noncleaved Cell Lymphoma” OR “Small Noncleaved-Cell Lymphomas” OR “Diffuse Undifferentiated Lymphoma” OR “Diffuse Undifferentiated Lymphomas” OR “Lymphoma, Diffuse Undifferentiated” OR “Undifferentiated Lymphoma, Diffuse” OR “Lymphoma, Small Non-Cleaved-Cell” OR “Lymphoma, Small Non Cleaved Cell” OR “Non-Cleaved-Cell Lymphoma, Small” OR “Small Non-Cleaved-Cell Lymphomas” OR “Small Non-Cleaved-Cell Lymphoma” OR “Small Non Cleaved Cell Lymphoma” OR “Lymphoma, Undifferentiated, Diffuse” OR “Lymphoma, Intermediate-Grade” OR “Intermediate-Grade Lymphoma” OR “Intermediate-Grade Lymphomas” OR “Lymphoma,	

	Intermediate Grade” OR “Lymphoma, Undifferentiated” OR “Lymphoma, Pleomorphic” OR “Pleomorphic Lymphoma” OR “Pleomorphic Lymphomas” OR “Undifferentiated Lymphoma” OR “Undifferentiated Lymphomas” OR “Reticulum-Cell Sarcoma” OR “Reticulum-Cell Sarcomas” OR “Sarcoma, Reticulum-Cell” OR “Sarcoma, Reticulum Cell” OR “Reticulum Cell Sarcoma” OR “Reticulum Cell Sarcomas” OR “Reticulosarcoma” OR “Reticulosarcomas” OR “Lymphoma, High-Grade” OR “High-Grade Lymphoma” OR “High-Grade Lymphomas” OR “Lymphoma, High Grade” OR “Lymphoma, Mixed-Cell, Diffuse” OR “Mixed Small and Large Cell Lymphoma, Diffuse” OR “Lymphoma, Mixed Small and Large Cell, Diffuse” OR “Lymphoma, Diffuse, Mixed Lymphocytic-Histiocytic” OR “Lymphoma, Small and Large Cleaved-Cell, Diffuse” OR “Lymphoma, Mixed Cell, Diffuse” OR “Mixed-Cell Lymphoma, Diffuse” OR “Mixed Cell Lymphoma, Diffuse” OR “Diffuse Mixed Small and Large Cell Lymphoma” OR “Diffuse Mixed-Cell Lymphoma” OR “Diffuse Mixed Cell Lymphoma” OR “Diffuse Mixed-Cell Lymphomas” OR “Lymphoma, Diffuse Mixed-Cel” OR “Non-Hodgkin's Lymphoma” OR “Non Hodgkin's Lymphoma” OR “Nonhodgkin's Lymphoma”)	
#4	#1 AND #2 AND #3	

Appendix 2. Countries and regions by UNAIDS

**UNAIDS Regions**	**Country**	**ISO Code**	**Unaids Regions**	**Country**	**ISO Code**
**Global**	—	03M49WLD	**Latin America**	Argentina	ARG
**Asia and the Pacific**	Afghanistan	AFG		Bolivia	BOL
	Australia	AUS		Brazil	BRA
	Bangladesh	BGD		Chile	CHL
	Bhutan	BTN		Colombia	COL
	Brunei	BRN		Costa Rica	CRI
	Cambodia	KHM		Ecuador	ECU
	China	CHN		El Salvador	SLV
	South Korea	PRK		Guatemala	GTM
	Micronesia	FSM		Honduras	HND
	Fiji	FJI		Mexico	MEX
	India	IND		Nicaragua	NIC
	Indonesia	IDN		Panama	PAN
	Japan	JPN		Paraguay	PRY
	Kiribati	KIR		Peru	PER
	Laos	LAO		Uruguay	URY
	Malaysia	MYS		Venezuela	VEN
	Maldives	MDV	**Middle East and North Africa**	Algeria	DZA
	Marshall Islands	MHL		Bahrain	BHR
	Mongolia	MNG		Djibouti	DJI
	Myanmar	MMR		Egypt	EGY
	Nauru	NRU		Iraq	IRQ

	Nepal	NPL		Iran	IRN
	New Zealand	NZL		Jordan	JOR
	Pakistan	PAK		Kuwait	KWT
	Palau	PLW		Lebanon	LBN
	Papua New Guinea	PNG		Libya	LBY
	Philippines	PHL		Morocco	MAR
	North Korea	KOR		Oman	OMN
	Samoa	WSM		Qatar	QAT
	Singapore	SGP		Saudi Arabia	SAU
	Solomon Islands	SLB		Somalia	SOM
	Sri Lanka	LKA		Sudan	SDN
	Thailand	THA		Syria	SYR
	Timor-Leste	TLS		Tunisia	TUN
	Tonga	TON		United Arab Emirates	ARE
	Tuvalu	TUV		Yemen	YEM
	Vanuatu	VUT	**Western and central Africa**	Benin	BEN
	Vietnam	VNM		Burkina Faso	BFA
**Caribbean**	Antigua	ATG		Burundi	BDI
	Bahamas	BMH		Ivory Coast	CIV
	Barbados	BRB		Cameroon	CMR
	Belize	BLZ		Cape Verde	CPV
	Cuba	CUB		Central African Republic	CAF
	Dominica	DMA		Chad	TCD
	Dominican Republic	DOM		Republic of Congo	COG
	Grenada	GRD		Democratic Republic of	COD

				the Congo	
	Guyana	GUY		Equatorial Guinea	GNQ
	Haiti	HTI		Gabon	GAB
	Jamaica	JAM		Gambia	GMB
	Saint Kitts	KNA		Ghana	GHA
	Saint Lucia	LCA		Guinea	GIN
	Saint Vincent	VCT		Guinea-Bissau	GNB
	Suriname	SUR		Liberia	LBR
	Trinidad	TTO		Mali	MLI
**Eastern and southern Africa**	Angola	AGO		Mauritania	MRT
	Botswana	BWA		Niger	NER
	Comoros	COM		Nigeria	NGA
	Eritrea	ERI		Sao Tome and Principe	STP
	Swaziland	SWZ		Senegal	SEN
	Ethiopia	ETH		Sierra Leone	SLE
	Kenya	KEN		Togo	TGO
	Lesotho	LSO	**Western and central Europe and North America**	Andorra	AND
	Madagascar	MDG		Austria	AUT
	Malawi	MWI		Belgium	BEL
	Mauritius	MUS		Bulgaria	BGR
	Mozambique	MOZ		Canada	CAN
	Namibia	NAM		Croatia	HRV
	Rwanda	RWA		Cyprus	CYP
	Seychelles	SYC		Czech Republic	CZE
	South Africa	ZAF		Denmark	DNK

	South Sudan	SSD		Estonia	EST
	Uganda	UGA		Finland	FIN
	Tanzania	TZA		France	FRA
	Zambia	ZMB		Germany	DEU
	Zimbabwe	ZWE		Greece	GRC
**Eastern Europe and central Asia**	Albania	ALB		Hungary	HUN
	Armenia	ARM		Iceland	ISL
	Azerbaijan	AZE		Ireland	IRL
	Belarus	BLR		Israel	ISR
	Bosnia and Herzegovina	BIH		Italy	ITA
	Georgia	GEO		Latvia	LVA
	Kazakhstan	KAZ		Lithuania	LTU
	Kyrgyzstan	KGZ		Luxembourg	LUX
	Montenegro	MNE		Netherlands	NLD
	Moldova	MDA		Norway	NOR
	Russia	RUS		Poland	POL
	Tajikistan	TJK		Portugal	PRT
	Micronesia (Federated States of)	MKD		Romania	ROU
	Turkmenistan	TKM		Serbia	SRB
	Ukraine	UKR		Slovakia	SVK
	Uzbekistan	UZB		Slovenia	SVN
				Spain	ESP
				Sweden	SWE
				Switzerland	CHE

				Turkey	TUR
				UK	GBR
				USA	USA
