# Supplementary material for: Global and regional burden estimation of HIV-associated non-Hodgkin’s lymphoma: a meta-analysis and modelling analysis protocol
Source: BMJ Open. 2024 Jun 25;14(6):e075933. doi: 10.1136/bmjopen-2023-075933 (PMC11210503; doi:10.1136/bmjopen-2023-075933)
Supplement: Author's manuscript [file bmjopen-2023-075933.draft_revisions.pdf]

BMJ Open is committed to open peer review. As part of this commitment we make the peer review history of every article we publish publicly available.

When an article is published we post the peer reviewers' comments and the authors' responses online. We also post the versions of the paper that were used during peer review. These are the versions that the peer review comments apply to.

The versions of the paper that follow are the versions that were submitted during the peer review process. They are not the versions of record or the final published versions. They should not be cited or distributed as the published version of this manuscript.

BMJ Open is an open access journal and the full, final, typeset and author-corrected version of record of the manuscript is available on our site with no access controls, subscription charges or pay-per-view fees (http://bmjopen.bmj.com).

If you have any questions on BMJ Open's open peer review process please email info.bmjopen@bmj.com

BMJ Open

Global and regional burden estimation of HIV-associated Non-Hodgkin Lymphoma: a meta-analysis and modeling analysis protocol

Journal:	*BMJ Open*
Manuscript ID	bmjopen-2023-075933
Article Type:	Protocol
Date Submitted by the Author:	23-May-2023

Complete List of Authors:	Chen, Yan; Southwest Medical University, School of Public Health<br>Sun, Ping; University of Electronic Science and Technology of China, Department of Health Management Center & Institute of Health Management<br>Sun, Zhaochen; Southwest Medical University, School of Public Health; University of Electronic Science and Technology of China, Department of Health Management Center & Institute of Health Management<br>Liu, Yuping; University of Electronic Science and Technology of China, Department of Health Management Center & Institute of Health Management<br>Wan, Zhengwei; University of Electronic Science and Technology of China, Department of Health Management Center & Institute of Health Management<br>Ye, Yunli; Southwest Medical University, School of Public Health

Keywords:	Lymphoma < HAEMATOLOGY, HIV & AIDS < INFECTIOUS DISEASES, Public health < INFECTIOUS DISEASES, Health policy < HEALTH SERVICES ADMINISTRATION & MANAGEMENT

SCHOLARONE™
Manuscripts

Global and regional burden estimation of HIV-associated Non-Hodgkin Lymphoma: a meta-analysis and modeling analysis protocol

Yan Chen[1,2], Ping Sun[2,3], Zhaochen Sun[1,2], Yuping Liu[2,3], Zhengwei Wan[2,3#], Yunli Ye[1#]

[1] School of Public Health, Southwest Medical University, Luzhou, China.

[2] Department of Health Management Center & Institute of Health Management, Sichuan Provincial People's Hospital, University of Electronic Science and Technology of China, Chengdu, China.

[3]Chinese Academy of Sciences Sichuan Translational Medicine Research Hospital, Chengdu, China.

Email address:

Yan Chen: chenyan122071@163.com

Ping Sun: sunping612@foxmail.com

Zhaochen Sun: 13585928551@163.com

Yuping Liu: 18981838972@163.com

[#] Corresponding authors

Prof. Yunli Ye

Address: No.1 First Section, Xianglin Rd, Longmatan Dist, Luzhou, China;

E-mail: wushuangyewu@163.com;

Tel: +86 18982785179.

Dr. Zhengwei Wan

Address: No. 32 West Second Section, First Ring Rd., Qingyang Dist., Chengdu, China;

E-mail: 18715799366@163.com;

Tel: +86 18715799366.

For peer review only

ABSTRACT

Introduction Human immunodeficiency virus (HIV) infection is one of the complex etiology of Non-Hodgkin lymphoma (NHL). However, the contribution of HIV to the disease burden of NHL across times and regions has yet to be comprehensively reported and quantified. Thus, the purpose of this study is to evaluate the relative risk of NHL in individuals with HIV-infection compared to those without HIV-infection by performing a comprehensive meta-analysis. Additionally, we intend to further estimate quantitatively the degree of HIV contributing to the burden of NHL with population attributable fraction (PAF) modeling analysis.

Methods and analysis This study will screen a mass of records searched from electronic databases (PubMed, EMBASE, Cochrane Library, and Web of Science). The main outcomes of this review are specific effect values and corresponding 95% confidence intervals (95% CI) for NHL among persons with HIV compared to those without HIV to quantify the association between HIV-infection and NHL. After quality assessment and data extraction, a meta-analysis will be performed to evaluate the pooled relative risk (RR) of NHL for HIV-infected population. Furthermore, the pooled RR value will be subjected to population attributable fraction (PAF) modeling analysis, combined with the number of age-specific disability-adjusted life years (DALY) and HIV prevalence data of 15 years-old and above from 1990 to 2019, at global, regional, and country levels. We will mainly calculate the PAF, HIV-associated DALYs number and age-standardized rates (ASRs) to quantify the burden of HIV-associated NHL.

Ethics and dissemination Our study is based on published articles thus the ethnic approval is not essential. In addition, we intend to publish the results on peer-reviewed journal for better discussion. We believe that conducting research on estimating the global burden of NHL can provide valuable insights for developing targeted prevention and control strategies, thereby achieving significant benefits.

For peer review only

PROSPERO registration number: CRD 42023404150.

Keywords: HIV, Non-Hodgkin Lymphoma, meta-analysis, population attributable fraction, global burden

Strengths and limitations of this study

1. The study will include meta-analysis and population attributable fraction estimation modeling analysis, which aiding in the formulation of precise prevention and control policies.

2. The quality of included studies will be assessed using an 11-iterm checklist recommended by the Agency for Healthcare Research and Quality (AHRQ).

3. The dissemination of the results may be limited because of the language restriction.

4. The pooled RR value will be applied in population attributable fraction modeling across the global, regions and all countries, which may dampens the estimated result.

For peer review only

Introduction

Non-Hodgkin lymphoma (NHL) is a common lymphoid tissues neoplasm that originates from B cell precursors, mature B cells, T cell precursors, and mature T cells.[1] The classification of Non-Hodgkin lymphoma (NHL) is intricate and dynamic, encompassing over 50 subtypes as listed in the World Health Organization (WHO) classification of 2016, with common subtypes including diffuse large B cell lymphoma (DLBCL), Burkitt lymphoma, follicular lymphoma, primary central nervous system (CNS) lymphoma, and Mantle cell lymphoma.[2 3] According to the GLOBOCAN database of WHO's International Agency for Research on Cancer (IARC), which includes 36 types of cancer worldwide in 2020, Non-Hodgkin lymphoma (NHL) accounted for 2.8% of total cancers with 544,352 new cases, and 2.6% of total cancer-related deaths with 259,793 new deaths, ranking NHL within the top 10 cancers for both males (ranked 8th) and females (ranked 10th).[4]

The etiology of NHL exhibits significant complexity. Published articles have identified various potential causes of NHL, including but not limited to immunosuppression, viral infection (eg, human immunodeficiency virus, HIV), family factor, occupational and environmental exposures, organ transplantation and autoimmune diseases.[5-7] Due to its association with immunosuppression, NHL is regarded as a defining event of acquired immune deficiency syndrome (AIDS),[6] thus establishing a strong connection between HIV and NHL. Based on a population-based review encompassing the United States, Australia and Italy, the risk of NHL among individuals with AIDS, when compared to the general population, ranged from 15-fold for low-grade NHL to 400-fold for high-grade NHL.[8] In addition, certain subtypes of NHL have been found to be associated with HIV infection. Another multicohort study conducted in Europe revealed a higher incidence of NHL among individuals with HIV, regardless of whether they received combination antiretroviral therapy (cART) treatment.[9] Although the introduction of cART in the late 1990s has significantly reduced the incidence of NHL,[10 11] such as the United States alone recorded 74,680 new NHL cancer diagnoses and 19,910 NHL-related deaths in 2018. However, the high burden of HIV infection in South Africa has

For peer review only

contributed to an increased incidence of Plasmablastic lymphoma (PBL), which is a subtype of NHL.[6 12]

Because of the significant variation of HIV-associated NHL burden, however, the contribution of HIV to the disease burden of NHL across times and regions has yet to be comprehensively reported and quantified. In this study, we aimed to perform a comprehensive meta-analysis to evaluate the relative risk of NHL in individuals with HIV-infection compared to those without HIV-infection. Additionally, we intend to further estimate quantitatively the degree of HIV contributing to the burden of NHL with population attributable fraction (PAF) modeling analysis. The anticipated outcome of this study is expected to provide valuable insights for the development of targeted prevention and control strategies for NHL.

Objectives

This study will aim to resolve questions:

1. Although NHL is considered an AIDS-defined disease, no meta-analysis has quantified the pooled relative risk of NHL in HIV-infected individuals compared to those without HIV infection.

2. The contribution of HIV to the disease burden of NHL across times and regions has yet to be comprehensively reported and quantified.

Methods and analysis

Our study will comprise two essential components as follow: 1) meta-analysis to evaluate relative risk of NHL for HIV-infected population; 2) PAF modeling analysis to assess HIV-associated burden of NHL.

1. Meta-analysis to assess the relative risk of NHL in HIV-infected population compared to people without HIV-infection

1.1 Registration

This protocol will follow to the guidelines of Preferred Reporting Items for Systematic reviews and Meta-Analyses for Protocols 2015 (PRISMA-P 2015)[13] and has registered

For peer review only

in the PROSPERO International prospective register of systematic reviews (https://www.crd.york.ac.uk/PROSPERO/) (CRD 42023404150).

1.2 Outcomes definition and eligibility criteria

The main outcomes of this review are specific effect values and corresponding 95% confidence intervals (95% CI) for NHL among persons with HIV compared to those without HIV to quantify the association between HIV and NHL. Effect values we intend to consider are risk ratio (RR), odds ratio (OR), standardized incidence rate (SIR), incidence rate ratio (IRR) and hazard ratio (HR).

For eligible studies, our target design types are cohort study, case-control study or cross-sectional study with effective control group and the studies expound the association between HIV and NHL using specific effect value. In addition, eligible studies should provide detailed participant information, including the total number of participants, the number of participants in the case and control groups, as well as the quantity of NHL cases in the case group. We will exclude records that are review articles, opinion pieces, poster presentations, letters, case reports and case series. For duplicate records, we will exclude incomplete or earlier period version. Subjects having overlap will also be eliminated.

1.3 Search strategy

We will search for English articles without restrictions of date or setting from four electronic databases: PubMed, Excerpta Medica database (EMBASE), Cochrane Library and Web of Science up to 31 March 2023 with keywords non-hodgkin lymphoma, NHL, Burkitt lymphoma, BL, diffuse large B-cell lymphoma, DLBCL, human immunodeficiency virus and HIV (i.e. NHL, subtypes of NHL, HIV and their equivalent terms). In addition, references of eligible records will be also searched to provide a more comprehensive searching strategy. The full electronic search strategies are included in the additional information (Appendix 1).

For peer review only

1.4 Data management and study selection

All retrieved records resulting from the search strings will be imported into Endnote X9.2 for the purposes of organizing and screening eligible studies. Research members will enact selection procedures and involved criteria in advance all together.

Step 1: Duplicate records and particular types screening

Two reviewers will initially utilize the built-in function of Endnote X9.2 to eliminate duplicate records. Subsequently, they will employ keyword searches within the software to exclude review articles and case reports, which are not relevant to the type of literature we require. Due to the limitation of incomplete duplication checking function, certain duplicate records still need to be manually removed. Thus, we leverage the advantage of two-person independent operation to reduce the possibility of omissions. Following the manual deletion process, the two reviewers will cross-check the exclusion results to ensure the highest level of completeness in the final record selection.

Step 2: Title and abstract screening

Next, two authors will review the titles and abstracts of the remaining records, excluding those that do not involve HIV population or outcome of NHL. If any records contain the required effect values in the abstracts, they will be considered potentially eligible. For records without abstract or with uncertain factors, the final decision will be made after finding the full text and evaluating whether they meet the inclusion criteria.

Step 3: Full text and eligible records assessment

The full texts of the provisionally selected records will be downloaded from databases. Two authors will conduct a thorough examination to identify the necessary information that can be utilized for statistical analyses, while simultaneously maintaining clear and comprehensive documentation of the relevant items. Articles will be ultimately included if they meet the eligibility criteria. Throughout the screening process, any

For peer review only

discrepancies or conflicts will be solved by consensus or arbitration from a third reviewer. Additionally, meticulous and detailed records will be maintained for each step of the screening process, which will facilitate the creation of a comprehensive flowchart and the composition of a formal article.

1.5 Data extraction

Two authors independently evaluated all retrieved articles by title, abstract, and full text according to the above-mentioned inclusion and exclusion criteria. Solution of discrepancies or conflicts will be same as the part of study selection. EndNote X9.2 will be used to manage the citations and extract data. The following data will be considered to extract (Table 1).

1.6 Quality assessment

All included studies will be independently assessed by two authors using an 11-item checklist recommended by the Agency for Healthcare Research and Quality (AHRQ) (https://www.wjx.cn/m/85284180.aspx). In this checklist, an item would be scored as '0' if the answer is 'NO' or 'UNCLEAR', and '1' if the answer is 'YES'. The determination of study quality will be complied with correspondence relationships as follows: low quality (0-3), moderate quality (4-7), high quality (8-11).[14] The judgement of studies' risk level will accord to the result of study quality assessment to divide into high, middle and low risk level corresponding to low, middle, high quality in sequence. Any disagreements between the two authors will be resolved according to the procedures outlined in the study selection process.

1.7 Statistical analysis

Overall statistical analyses and calculation will be completed by the R software (version 4.2.2). Firstly, we will calculate a pooled relative risk (RR) and the corresponding 95% confidence intervals (95% CI) to quantitatively estimate the association between HIV and NHL. Then the heterogeneity will be assessed using the Chi-squared test on Cochrane's Q statistic, and the quantitation of heterogeneity will be measured by the

For peer review only - http://bmjopen.bmj.com/site/about/guidelines.xhtml

Higgins's I^2 value presented as a percentage.[15] If the I^2 value exceeds 50%, subgroup analyses will be utilized to find the potential source of heterogeneity, including study conducting region, study design types, setting of study (multi-center or single-center), article quality risk level.

Furthermore, a meta-regression will be performed to ensure whether these factors can explain the heterogeneity and the degree to which they account for it. The estimation of pooled effect value will choose fixed effect model when I^2 value is less than or equal to 50%. Regarding sensitivity analysis, we intend to analysis influence of single study by omitting study one-by-one and different risk levels. Furthermore, we will also use funnel plot and Egger's linear regression (if outcomes with more than 10 individual studies) to find potential publication bias from these articles. If publication bias is detected, we will address it using the trim-and-fill method to adjust for the bias.

2. Population attributable fraction modeling analysis for HIV-associated burden of NHL estimation

2.1 Information sources

The number of age-specific disability-adjusted life years (DALY) will be obtained from the Global Health Data Exchange (GHDx) query tool (https://vizhub.healthdata.org/gbd-results/) in 5 years-old intervals. We will download national or territorial HIV prevalence of 15 years-old and above from 1990 to 2019 from the Joint United Nations Programme on HIV/AIDS (UNAIDS) (https://aidsinfo.unaids.org/). The criteria for dividing countries and regions will be based on UNAIDS classifications using ISO codes alpha-3 (Appendix 2).

2.2 Population attributable fraction modeling implement

Once we have obtained the relative risk (RR) value from the meta-analysis, we will proceed with the population attributable fraction (PAF) modeling to estimate the burden of HIV-associated NHL (including HIV associated disability-adjusted life years

For peer review only

(DALYs), and age-standardized DALYs rate) following the following steps.

Step 1: Population attributable fraction (PAF, %) calculation

We will calculate PAF combined with HIV prevalence and RR value, with the following assumptions: 1) the pooled RR is applicable across the global, regions and all countries; 2) we ignore various confounding biases between primary studies, which may cause slight deviation in our estimation; 3) we assume the RR fits log normal distribution and beta distribution for the HIV prevalence, then we calculate 10,000 samples of PAF to obtain uncertainty intervals (2.5%, 97.5%) by R software. We will estimate the PAF using the formula as follows:

$$PAF = \frac{HIV\ prevalence * (RR - 1)}{1 + HIV\ prevalence * (RR - 1)}$$

Figure 1 illustrates the calculation steps of PAF, using South Africa as an example.

Step 2: Estimation of NHL disability-adjusted life years (DALYs) attributable to HIV (HIV-associated DALYs)

We calculate age standardized incidence rates (ASRs) per 100,000 population of all-cause disability-adjusted life years (DALYs) and corresponding 95% confidence intervals by direct method, based on the age-specific all-cause NHL DALYs and world standard population reported in the GBD 2019. Formulas for calculating the ASR as below:

1) for each age group:

$$Crude\ Rate = \frac{Case}{Population}$$

2)

$$Proportion = \frac{Age\ Group}{\sum Age\ Group}$$

3) for each age group: $Adjust\ Rate = Crude\ Rate * Proportion$

4) for ⩾15 years old: $Adjust\ Rate = 100000 * sum\ (Adjust\ Rates)$

Then we assume that both number of DALYs and ASRs fit log normal distribution and we calculate 10,000 samples of HIV-associated DALYs and ASRs to obtain uncertainty intervals (2.5%, 97.5%) by R software. We estimate the HIV-associated DALYs

number and ASRs using fomulas as below:

1) $DALY_{\text{attributed to HIV}} = All\_cause\ DALY * PAF$

2) $DALY\ ASR_{\text{attributed to HIV}} = All\_cause\ DALYs\ ASR * PAF$

Figure 2 shows the calculation steps of the HIV-associated NHL DALYs for South Africa.

Discussion

The incidence rate of NHL exhibited a dramatic increase starting around 1970, and despite experiencing a plateau in between, it still recorded over 500 thousand of new cases in 2020.[4 16] The complex etiology of NHL, coupled with the accelerated aging process of global population, is expected to further contributed to an increase of patients and deaths number for NHL.[6 16] According to the UNAIDS, there are approximately 4,000 new HIV infections reported daily, with 1,100 of those are young people (aged 15 to 24 years).[17] Given to the close relationship between HIV and the NHL, it is crucial to be concerned about the trajectory of the burden of HIV-associated NHL.

Our study aims to investigate the risk of non-Hodgkin lymphoma (NHL) in individuals with HIV infection compared to those without HIV, and to quantify the contribution of HIV to the incidence of NHL. To ensure the readability of the literature, we will only include articles published in English, which may limit the generalizability of our findings. Regarding the dissemination of our results, we will make every effort to publish them in a peer-reviewed journal as an academic article, and also present our findings at relevant field meetings. Since this review is based on published data, an ethical review is not required. Considering the global impact of HIV-associated NHL, we believe that conducting research on estimating the global burden of the disease can provide valuable insights for developing targeted prevention and control strategies, thereby achieving significant benefits.

Authors' contributions YC, YLY, ZWW, PS and ZCS designed the study protocol. YC and ZCS prepared the first draft. YLY, ZWW and YPL reviewed and revised the

For peer review only

first draft. All authors read and approved the final manuscript.

Funding statement This work was supported by [Science Foundation for Young Scholars of Sichuan Provincial People's Hospital] grant number [NO. 2022QN18] and [Subject Founds of Health Care for Cadres of Sichuan Province] grant number [NO. 2022-220].

Competing interests The authors declared that they had no competing interests.

Patient consent for publication Not required.

References

1. Sapkota S, Shaikh H. Non-Hodgkin Lymphoma. StatPearls. Treasure Island (FL): StatPearls Publishing; 2023 Jan-.2023.
2. Armitage JO, Weisenburger DD. New approach to classifying non-Hodgkin's lymphomas: clinical features of the major histologic subtypes. Non-Hodgkin's Lymphoma Classification Project. *J Clin Oncol* 1998;16(8):2780-95. doi: 10.1200/JCO.1998.16.8.2780 [published Online First: 1998/08/15]
3. Swerdlow SH, Campo E, Pileri SA, et al. The 2016 revision of the World Health Organization classification of lymphoid neoplasms. *Blood* 2016;127(20):2375-90. doi: 10.1182/blood-2016-01-643569 [published Online First: 2016/03/17]
4. Sung H, Ferlay J, Siegel RL, et al. Global Cancer Statistics 2020: GLOBOCAN Estimates of Incidence and Mortality Worldwide for 36 Cancers in 185 Countries. *CA Cancer J Clin* 2021;71(3):209-49. doi: 10.3322/caac.21660 [published Online First: 2021/02/05]
5. Muller AM, Ihorst G, Mertelsmann R, et al. Epidemiology of non-Hodgkin's lymphoma (NHL): trends, geographic distribution, and etiology. *Ann Hematol* 2005;84(1):1-12. doi: 10.1007/s00277-004-0939-7 [published Online First: 2004/10/14]
6. Bispo JAB, Pinheiro PS, Kobetz EK. Epidemiology and Etiology of Leukemia and Lymphoma. *Cold Spring Harb Perspect Med* 2020;10(6) doi: 10.1101/cshperspect.a034819 [published Online

For peer review only

First: 2019/11/16]

7. Rana I, Dahlberg S, Steinmaus C, et al. Benzene exposure and non-Hodgkin lymphoma: a systematic review and meta-analysis of human studies. *Lancet Planet Health* 2021;5(9):e633-e43. doi: 10.1016/S2542-5196(21)00149-2 [published Online First: 2021/08/28]

8. Dal Maso L, Franceschi S. Epidemiology of non-Hodgkin lymphomas and other haemolymphopoietic neoplasms in people with AIDS. *Lancet Oncol* 2003;4(2):110-9. doi: 10.1016/s1470-2045(03)00983-5 [published Online First: 2003/02/08]

9. Collaboration of Observational HIVERESG, Bohlius J, Schmidlin K, et al. Incidence and risk factors of HIV-related non-Hodgkin's lymphoma in the era of combination antiretroviral therapy: a European multicohort study. *Antivir Ther* 2009;14(8):1065-74. doi: 10.3851/IMP1462 [published Online First: 2009/12/25]

10. Jacobson LP, Yamashita TE, Detels R, et al. Impact of potent antiretroviral therapy on the incidence of Kaposi's sarcoma and non-Hodgkin's lymphomas among HIV-1-infected individuals. Multicenter AIDS Cohort Study. *J Acquir Immune Defic Syndr* 1999;21 Suppl 1:S34-41. [published Online First: 1999/08/03]

11. Polesel J, Clifford GM, Rickenbach M, et al. Non-Hodgkin lymphoma incidence in the Swiss HIV Cohort Study before and after highly active antiretroviral therapy. *AIDS* 2008;22(2):301-6. doi: 10.1097/QAD.0b013e3282f2705d [published Online First: 2007/12/22]

12. Vaughan J, Perner Y, Mayne E, et al. Plasmablastic lymphoma in Johannesburg, South Africa, in the era of widescale antiretroviral therapy use. *HIV Med* 2021;22(3):225-30. doi: 10.1111/hiv.12965 [published Online First: 2020/10/07]

13. Moher D, Shamseer L, Clarke M, et al. Preferred reporting items for systematic review and meta-analysis protocols (PRISMA-P) 2015 statement. *Syst Rev* 2015;4(1):1. doi: 10.1186/2046-4053-4-1 [published Online First: 2015/01/03]

14. Hu J, Dong Y, Chen X, et al. Prevalence of suicide attempts among Chinese adolescents: A meta-analysis of cross-sectional studies. *Compr Psychiatry* 2015;61:78-89. doi: 10.1016/j.comppsych.2015.05.001 [published Online First: 2015/05/26]

15. Higgins JP, Thompson SG. Quantifying heterogeneity in a meta-analysis. *Stat Med* 2002;21(11):1539-58. doi: 10.1002/sim.1186 [published Online First: 2002/07/12]

16. Skrabek P, Turner D, Seftel M. Epidemiology of non-Hodgkin lymphoma. *Transfus Apher Sci*

For peer review only

2013;49(2):133-8. doi: 10.1016/j.transci.2013.07.014 [published Online First: 2013/08/21]

17. UNAIDS. Joint United Nations Programme on HIV/AIDS. IN DANGER: UNAIDS Global AIDS Update 2022. 2022. https://www.unaids.org/en/resources/documents/2022/in-danger-global-aids-update. accessed 14 May 2023.

For peer review only

Table 1. Data to be extracted from eligible articles

	Data
Publication details	Author, publication year
Type of design	Cohort study, CCS or cross-sectional study with effective control group
Information of study	Time period of study, country and region of research conducted, study setting (multi-center or single-center), disease classification criterion used, data source or name of cohort registered
Effect values	RR, OR, SIR, IRR and HR and their corresponding 95% CI
Study participants	Total number of HIV patients, total number of control individuals, number of NHL in the case group and number of control group

Abbreviations: CCS-Case-control study; RR-Risk ratio; OR-Odds ratio; SIR-Standardized incidence rate; IRR-Incidence rate ratio; HR-Hazard ratio; CI-Confidence intervals; HIV-Human immunodeficiency virus; NHL-Non-Hodgkin lymphoma

Figure legends:

Figure 1. Calculation steps of PAF for South Africa

Schematic diagram of calculating PAF, using South Africa as an example.

Figure 2. Calculation steps of the HIV-associated NHL DALYs for South Africa

Schematic diagram of calculating the HIV-associated NHL DALYs, using South Africa as an example.

For peer review only

South Africa in 2000:

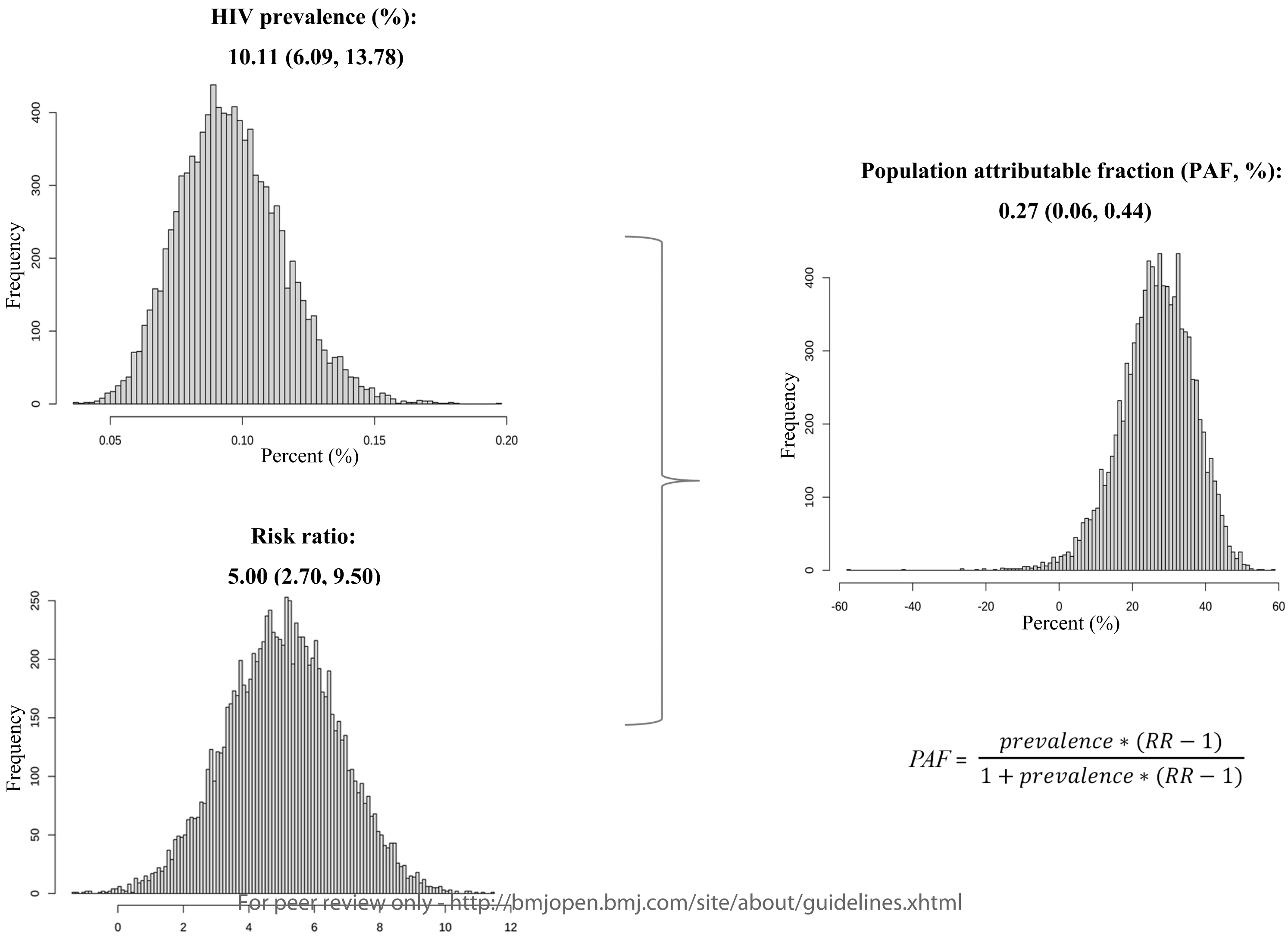

$$PAF = \frac{prevalence * (RR - 1)}{1 + prevalence * (RR - 1)}$$

For peer review only - http://bmjopen.bmj.com/site/about/guidelines.xhtml

South Africa in 2000:

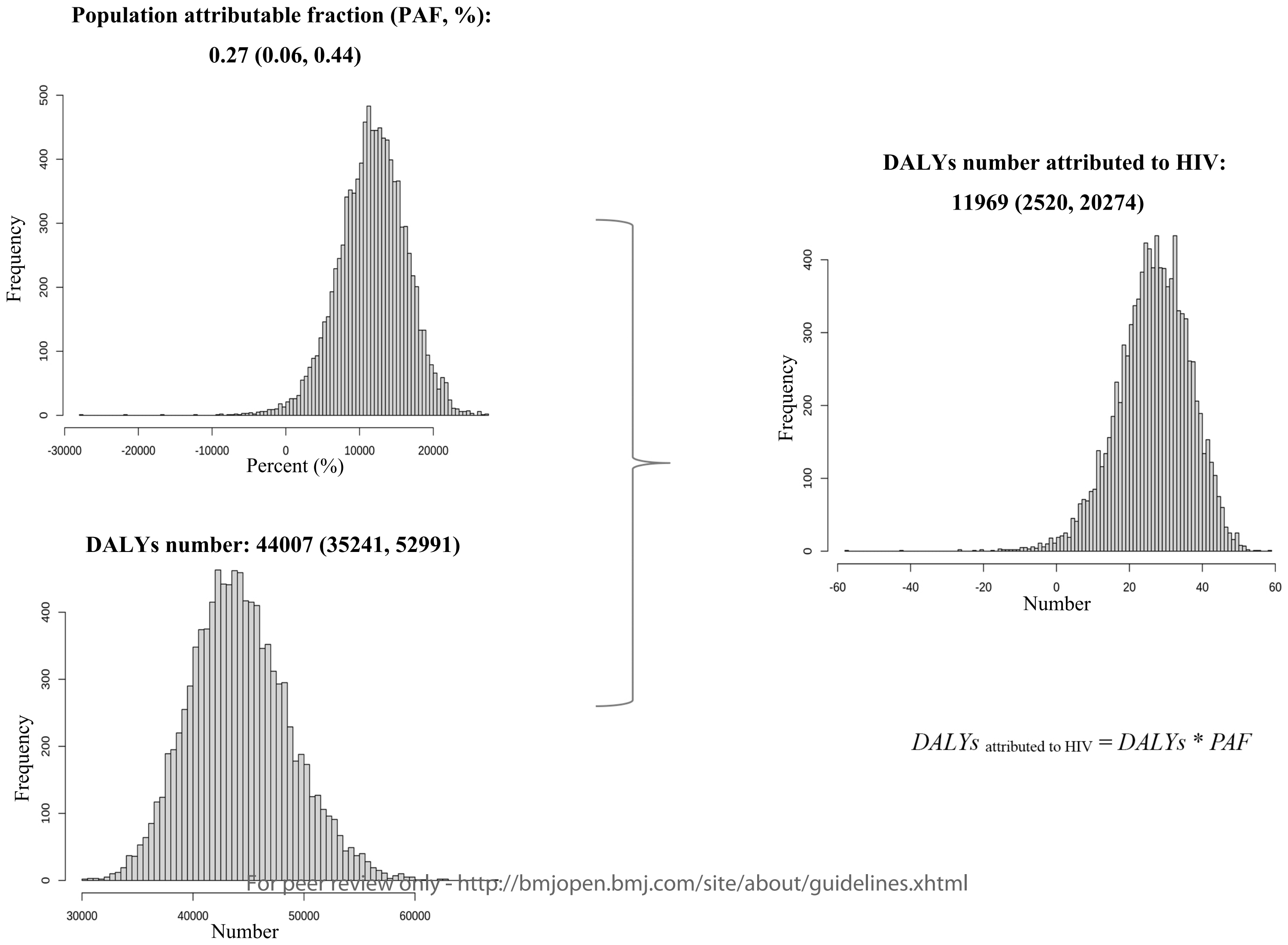

For peer review only - http://bmjopen.bmj.com/site/about/guidelines.xhtml

Appendix

Appendix 1. Electronic databases search strategies in PubMed, Embase, Cochrane Library and Web of Science

Pubmed

Set	Search	Results
#1	"lymphoma, non hodgkin"[MeSH Terms]	
#2	"hiv"[Title/Abstract] OR "hiv-1"[Title/Abstract] OR "hiv 1"[Title/Abstract] OR "hiv 2"[Title/Abstract] OR "hiv-2"[Title/Abstract] OR "human immunodeficiency virus"[Title/Abstract] OR "human immuno deficiency virus"[Title/Abstract] OR "human immunedeficiency virus"[Title/Abstract] OR "human immune deficiency virus"[Title/Abstract] OR "acquired immunodeficiency syndrome"[MeSH Terms] OR "acquired immunodeficiency syndrome"[Title/Abstract] OR "acquired immuno-deficiency syndrome"[Title/Abstract]OR "acquired immunedeficiency syndrome"[Title/Abstract]	
#3	"lymphoma non hodgkin"[Title/Abstract] OR (("Lymphoma"[MeSH Terms] OR "Lymphoma"[All Fields] OR "Lymphomas"[All Fields] OR "lymphoma s"[All Fields]) AND "small cleaved cell diffuse"[Title/Abstract]) OR ((("Lymphoma"[MeSH Terms] OR "Lymphoma"[All Fields] OR "Lymphomas"[All Fields] OR "lymphoma s"[All Fields]) AND ("atypic"[All Fields] OR "atypical"[All Fields] OR "atypicalities"[All Fields] OR "atypicality"[All Fields] OR "atypically"[All Fields] OR "atypicals"[All Fields] OR "atypism"[All Fields] OR "atypisms"[All Fields]) AND ("diffusable"[All Fields] OR "diffusant"[All Fields] OR "diffusants"[All Fields] OR "Diffuse"[All Fields] OR "diffusely"[All Fields] OR "diffuses"[All Fields] OR "diffusibility"[All Fields] OR "diffusible"[All Fields] OR "diffusion"[MeSH Terms] OR "diffusion"[All Fields] OR "diffused"[All Fields] OR "diffusing"[All Fields] OR "diffusions"[All Fields] OR "diffusive"[All Fields] OR "diffusively"[All Fields] OR "diffusivities"[All Fields] OR "diffusivity"[All Fields])) AND "small lymphoid"[Title/Abstract]) OR "diffuse small cleaved cell lymphoma"[Title/Abstract] OR "diffuse small cleaved cell lymphoma"[Title/Abstract] OR (("Lymphoma"[MeSH Terms] OR "Lymphoma"[All Fields] OR "Lymphomas"[All Fields] OR "lymphoma s"[All Fields]) AND "small cleaved cell diffuse"[Title/Abstract]) OR (("Lymphoma"[MeSH Terms] OR "Lymphoma"[All Fields] OR "Lymphomas"[All Fields] OR "lymphoma s"[All Fields]) AND "Nonhodgkins"[Title/Abstract]) OR "nonhodgkins lymphoma"[Title/Abstract] OR "lymphoma non hodgkins"[Title/Abstract] OR "lymphoma non hodgkins"[Title/Abstract] OR "non hodgkins lymphoma"[Title/Abstract] OR "non hodgkin lymphoma"[Title/Abstract] OR "non hodgkin lymphoma"[Title/Abstract] OR "lymphoma nonhodgkin s"[Title/Abstract] OR	

For peer review only

	"lymphoma nonhodgkin"[Title/Abstract] OR "small cleaved cell lymphoma diffuse"[Title/Abstract] OR "small cleaved cell lymphoma diffuse"[Title/Abstract] OR "lymphoma non hodgkin s"[Title/Abstract] OR "lymphoma non hodgkin s"[Title/Abstract] OR "lymphoma diffuse"[Title/Abstract] OR "diffuse lymphoma"[Title/Abstract] OR "diffuse lymphomas"[Title/Abstract] OR "sarcoma lymphatic"[Title/Abstract] OR "Lymphosarcoma"[Title/Abstract] OR "Lymphosarcomas"[Title/Abstract] OR "lymphatic sarcoma"[Title/Abstract] OR "lymphatic sarcomas"[Title/Abstract] OR "lymphoma low grade"[Title/Abstract] OR "low grade lymphoma"[Title/Abstract] OR "low grade lymphomas"[Title/Abstract] OR "lymphoma low grade"[Title/Abstract] OR "lymphoma mixed cell"[Title/Abstract] OR "lymphoma mixed cell"[Title/Abstract] OR "mixed cell lymphoma"[Title/Abstract] OR "mixed cell lymphoma"[Title/Abstract] OR "mixed cell lymphomas"[Title/Abstract] OR "lymphoma mixed"[Title/Abstract] OR "mixed lymphoma"[Title/Abstract] OR "mixed lymphomas"[Title/Abstract] OR "lymphoma mixed lymphocytic histiocytic"[Title/Abstract] OR ("Lymphocytic-Histiocytic"[All Fields] AND "lymphoma mixed"[Title/Abstract]) OR ("Lymphocytic-Histiocytic"[All Fields] AND "lymphomas mixed"[Title/Abstract]) OR "lymphoma mixed lymphocytic histiocytic"[Title/Abstract] OR "mixed lymphocytic histiocytic lymphoma"[Title/Abstract] OR "mixed lymphocytic histiocytic lymphomas"[Title/Abstract] OR (("lymphoma, non hodgkin"[MeSH Terms] OR ("Lymphoma"[All Fields] AND "Non-Hodgkin"[All Fields]) OR "non-hodgkin lymphoma"[All Fields] OR ("Lymphoma"[All Fields] AND "Non"[All Fields] AND "Hodgkin"[All Fields]) OR "lymphoma non hodgkin"[All Fields]) AND "Familial"[Title/Abstract]) OR "lymphoma small noncleaved cell"[Title/Abstract] OR "lymphoma small noncleaved cell"[Title/Abstract] OR "small noncleaved cell lymphoma"[Title/Abstract] OR ("Noncleaved-Cell"[All Fields] AND "lymphoma small"[Title/Abstract]) OR "small noncleaved cell lymphoma"[Title/Abstract] OR "small noncleaved cell lymphomas"[Title/Abstract] OR "diffuse undifferentiated lymphoma"[Title/Abstract] OR "diffuse undifferentiated lymphomas"[Title/Abstract] OR "lymphoma diffuse undifferentiated"[Title/Abstract] OR "undifferentiated lymphoma diffuse"[Title/Abstract] OR "lymphoma small non cleaved cell"[Title/Abstract] OR "lymphoma small non cleaved cell"[Title/Abstract] OR ("Non-Cleaved-Cell"[All Fields] AND "lymphoma small"[Title/Abstract]) OR "small non cleaved cell lymphomas"[Title/Abstract] OR "small non cleaved cell lymphoma"[Title/Abstract] OR "small non cleaved cell lymphoma"[Title/Abstract] OR (("Lymphoma"[MeSH Terms] OR "Lymphoma"[All Fields] OR "Lymphomas"[All Fields] OR "lymphoma s"[All Fields]) AND "undifferentiated diffuse"[Title/Abstract]) OR "lymphoma intermediate grade"[Title/Abstract] OR "intermediate grade lymphoma"[Title/Abstract] OR "intermediate grade lymphomas"[Title/Abstract]	

	OR "lymphoma intermediate grade"[Title/Abstract] OR "lymphoma undifferentiated"[Title/Abstract] OR "lymphoma pleomorphic"[Title/Abstract] OR "pleomorphic lymphoma"[Title/Abstract] OR "diffuse mixed cell lymphomas"[Title/Abstract] OR "diffuse mixed cell lymphoma"[Title/Abstract] OR "diffuse mixed cell lymphoma"[Title/Abstract] OR "non hodgkin s lymphoma"[Title/Abstract] OR "non hodgkin s lymphoma"[Title/Abstract] OR "nonhodgkin s lymphoma"[Title/Abstract] OR ("diffuse mixed small"[Title/Abstract] AND "large cell lymphoma"[Title/Abstract]) OR (("Mixed"[All Fields] OR "mixes"[All Fields] OR "mixing"[All Fields] OR "mixings"[All Fields]) AND "cell lymphoma diffuse"[Title/Abstract]) OR ("Mixed-Cell"[All Fields] AND "lymphoma diffuse"[Title/Abstract]) OR (("Lymphoma"[MeSH Terms] OR "Lymphoma"[All Fields] OR "Lymphomas"[All Fields] OR "lymphoma s"[All Fields]) AND "mixed cell diffuse"[Title/Abstract]) OR ("lymphoma small"[Title/Abstract] AND ((("Large"[All Fields] OR "largely"[All Fields] OR "larges"[All Fields]) AND "Cleaved-Cell"[All Fields]) AND "Diffuse"[Title/Abstract])) OR (("lymphoma, non hodgkin"[MeSH Terms] OR ("Lymphoma"[All Fields] AND "Non-Hodgkin"[All Fields]) OR "non-hodgkin lymphoma"[All Fields] OR ("Lymphoma"[All Fields] AND "Diffuse"[All Fields]) OR "lymphoma diffuse"[All Fields]) AND "mixed lymphocytic histiocytic"[Title/Abstract]) OR ("lymphoma mixed small"[Title/Abstract] AND "large cell diffuse"[Title/Abstract]) OR ("mixed small"[Title/Abstract] AND "large cell lymphoma diffuse"[Title/Abstract]) OR (("Lymphoma"[MeSH Terms] OR "Lymphoma"[All Fields] OR "Lymphomas"[All Fields] OR "lymphoma s"[All Fields]) AND "mixed cell diffuse"[Title/Abstract]) OR "lymphoma high grade"[Title/Abstract] OR "high grade lymphomas"[Title/Abstract] OR "high grade lymphoma"[Title/Abstract] OR "lymphoma high grade"[Title/Abstract] OR "Reticulosarcomas"[Title/Abstract] OR "Reticulosarcoma"[Title/Abstract] OR "reticulum cell sarcomas"[Title/Abstract] OR "reticulum cell sarcoma"[Title/Abstract] OR "sarcoma reticulum cell"[Title/Abstract] OR "sarcoma reticulum cell"[Title/Abstract] OR "reticulum cell sarcomas"[Title/Abstract] OR "reticulum cell sarcoma"[Title/Abstract] OR "undifferentiated lymphomas"[Title/Abstract] OR "undifferentiated lymphoma"[Title/Abstract] OR "pleomorphic lymphomas"[Title/Abstract]	
#4	"prevalence"[Text Word] OR "incidence"[Text Word] OR "epidemiol*"[Text Word] OR "survey"[Text Word] OR "rapid assessment"[Text Word] OR "situation assessment"[Text Word] OR "situational assessment"[Text Word] OR "rar"[Text Word] OR "cohort"[Text Word] OR "surveillance"[Text Word] OR "seroprevalence"[Text Word] OR "seroincidence"[Text Word] OR "screening"[Text Word] OR "mortality"[Title/Abstract] OR "mortalities"[Title/Abstract] OR "mortal"[Title/Abstract] OR "dead"[Title/Abstract] OR "death"[Title/Abstract] OR "deaths"[Title/Abstract] OR "decease*"[Title/Abstract] OR "demise*"[Title/Abstract] OR "die"[Title/Abstract] OR "died"[Title/Abstract] OR "dies"[Title/Abstract] OR	

For peer review only

	"dying"[Title/Abstract] OR "fatal"[Title/Abstract] OR "fatality"[Title/Abstract] OR "fatalities"[Title/Abstract] OR "nonsurvival*"[Title/Abstract] OR "nonsurvival*"[Title/Abstract] OR "survival*"[Title/Abstract] OR "lethality"[Title/Abstract] OR "epidemiologic methods"[MeSH Major Topic] OR "epidemiologic studies"[MeSH Major Topic] OR "sentinel surveillance"[MeSH Major Topic] OR "seroepidemiologic studies"[MeSH Major Topic] OR "cohort studies"[MeSH Major Topic] OR "longitudinal studies"[MeSH Major Topic] OR "follow up studies"[MeSH Major Topic] OR "prospective studies"[MeSH Major Topic] OR "diagnostic services"[MeSH Major Topic] OR "mass screening"[MeSH Major Topic] OR "mortality"[MeSH Major Topic] OR "hospital mortality"[MeSH Major Topic] OR "death"[MeSH Major Topic] OR "cause of death"[MeSH Major Topic] OR "death certificates"[MeSH Major Topic] OR "fatal outcome"[MeSH Major Topic] OR "autopsy"[MeSH Major Topic] OR "survival rate"[MeSH Major Topic]	
#5	#1 or #2	
#6	#3 and #4 and #5	

Embase

Set	Search	Results
#1	'human immunodeficiency virus infection'/exp OR 'human immunodeficiency virus'/exp OR 'hiv survivor'/exp OR hiv:ti,ab,kw OR 'hiv-1':ti,ab,kw OR 'hiv-2':ti,ab,kw OR 'human immunodeficiency virus':ti,ab,kw OR 'human immunodeficiency':ti,ab,kw OR 'human immunedeficiency virus':ti,ab,kw OR 'human immune deficiency virus':ti,ab,kw OR 'human immune-deficiency virus':ti,ab,kw OR 'acquired immune-deficiency syndrome':ti,ab,kw OR 'acquired immunedeficiency syndrome':ti,ab,kw OR 'acquired immunodeficiency syndrome':ti,ab,kw OR 'acquired immuno-deficiency syndrome':ti,ab,kw	
#2	prevalence:ti,ab,de OR incidence:ti,ab,de OR epidemiol*:ti,ab,de OR survey:ti,ab,de OR 'rapid assessment':ti,ab,de OR 'situation assessment':ti,ab,de OR 'situational assessment':ti,ab,de OR rar:ti,ab,de OR cohort:ti,ab,de OR surveillance:ti,ab,de OR seroprevalence:ti,ab,de OR seroincidence:ti,ab,de OR seroepidemiol*:ti,ab,de OR screening:ti,ab,de OR 'mass screening':ti,ab,de OR mortality:ti,ab OR mortalities:ti,ab OR mortal:ti,ab OR dead:ti,ab OR death:ti,ab OR deaths:ti,ab OR decease*:ti,ab OR demise*:ti,ab OR die:ti,ab OR died:ti,ab OR dies:ti,ab OR dying:ti,ab OR fatal:ti,ab OR fatality:ti,ab OR fatalities:ti,ab OR 'non survival*':ti,ab OR nonsurvival*:ti,ab OR survival*:ti,ab OR lethality:ti,ab OR epidemiology:de OR 'cancer epidemiology'/exp OR 'cancer statistics'/exp OR 'disease surveillance'/exp OR 'health survey'/exp OR 'incidence'/exp OR 'infection rate'/exp OR 'prevelance' OR 'sentinel surveillance'/exp OR 'seroepidemiology'/exp OR 'cohort analysis'/exp OR 'longitudinal study'/exp OR 'prospective study'/exp OR 'preventive health service'/exp OR 'mass	

For peer review only

	screening'/exp OR 'cancer mortality'/exp OR 'all cause mortality'/exp OR 'mortality':de OR 'hospital mortality'/exp OR 'mortality rate'/exp OR 'death'/exp OR 'cause of death'/exp OR 'dying'/exp OR 'fatality'/exp OR 'lethality'/exp OR 'death certificate'/exp OR 'autopsy'/exp OR 'cancer survival'/exp OR 'survival rate'/exp	
#3	'non-hodgkin lymphoma'/exp	
#4	'lymphoma, non hodgkin':ab,ti	
#5	'lymphoma, small cleaved-cell, diffuse':ab,ti	
#6	'lymphoma, atypical diffuse small lymphoid':ab,ti	
#7	'diffuse small cleaved-cell lymphoma':ab,ti	
#8	'diffuse small cleaved cell lymphoma':ab,ti	
#9	'lymphoma, small cleaved cell, diffuse':ab,ti	
#10	'lymphoma, nonhodgkins':ab,ti	
#11	'nonhodgkins lymphoma':ab,ti	
#12	'lymphoma, non-hodgkins':ab,ti	
#13	'lymphoma, non hodgkins':ab,ti	
#14	'lymphoma, non hodgkins':ab,ti	
#15	'non-hodgkins lymphoma':ab,ti	
#16	'non-hodgkin lymphoma':ab,ti	
#17	'lymphoma, nonhodgkin':ab,ti	
#18	'small cleaved-cell lymphoma, diffuse':ab,ti	
#19	'lymphoma, diffuse':ab,ti	
#20	'ldiffuse lymphomae':ab,ti	
#21	'diffuse lymphomae':ab,ti	
#22	'diffuse lymphomas':ab,ti	
#23	'diffuse lymphoma':ab,ti	
#24	'sarcoma, lymphatic':ab,ti	
#25	'lymphosarcoma':ab,ti	
#26	'lymphosarcomas':ab,ti	
#27	'llymphatic sarcomas':ab,ti	
#28	'lymphatic sarcomas':ab,ti	
#29	'lymphatic sarcomas':ab,ti	
#30	'lymphoma, low-grade':ab,ti	
#31	'low-grade lymphoma':ab,ti	
#32	'low-grade lymphomas':ab,ti	
#33	'lymphoma, low grade':ab,ti	
#34	'lymphoma, mixed-cell':ab,ti	
#35	'lymphoma, mixed cell':ab,ti	
#36	'mixed-cell lymphoma':ab,ti	
#37	'mixed cell lymphoma':ab,ti	
#38	'mixed-cell lymphomas':ab,ti	
#39	'lymphoma, mixed':ab,ti	
#40	'mixed lymphoma':ab,ti	

For peer review only

#41	'lymphoma, mixed lymphocytic-histiocytic':ab,ti	
#42	'lymphocytic-histiocytic lymphoma, mixed':ab,ti	
#43	'mixed lymphocytic-histiocytic lymphoma':ab,ti	
#44	'mixed lymphocytic-histiocytic lymphomas':ab,ti	
#45	'lymphoma, non-hodgkin, familial':ab,ti	
#46	'lymphoma, small noncleaved-cell':ab,ti	
#47	'lymphoma, small noncleaved cell':ab,ti	
#48	'small noncleaved-cell lymphoma':ab,ti	
#49	'noncleaved-cell lymphoma, small':ab,ti	
#50	'small noncleaved cell lymphoma':ab,ti	
#51	'small noncleaved-cell lymphomas':ab,ti	
#52	'diffuse undifferentiated lymphoma':ab,ti	
#53	'diffuse undifferentiated lymphomas':ab,ti	
#54	#3 OR #4 OR #5 OR #6 OR #7 OR #8 OR #9 OR #10 OR #11 OR #12 OR #13 OR #14 OR #15 OR #16 OR #17 OR #18 OR #19 OR #20 OR #21 OR #22 OR #23 OR #24 OR #25 OR #26 OR #27 OR #28 OR #29 OR #30 OR #31 OR #32 OR #33 OR #34 OR #35 OR #36 OR #37 OR #38 OR #39 OR #40 OR #41 OR #42 OR #43 OR #44 OR #45 OR #46 OR #47 OR #48 OR #49 OR #50 OR #51 OR #52 OR #53	
#55	#1 AND #2 AND #54	

Cochrane Library

Set	Search	Results
#1	MeSH descriptor: [HIV] explode all trees	
#2	(hiv):ti,ab,kw (Word variations have been searched)	
#3	#1 OR #2	
#4	("non-Hodgkin lymphoma"):ti,ab,kw (Word variations have been searched)	
#5	MeSH descriptor: [Lymphoma, Non-Hodgkin] explode all trees	
#6	#4 OR #5	
#7	#6 OR #3	

Web of Science

Set	Search	Results
#1	"TS=(prevalence OR incidence OR epidemiol* OR survey OR ""rapid assessment"" OR ""situation assessment"" OR ""situational assessment"" OR rar OR cohort OR surveillance OR seroprevalence OR seroincidence OR seroepidemiol* OR screening OR mortality OR mortalities OR mortal OR dead OR death OR deaths OR decease* OR demise* OR die OR died OR dies OR dying OR fatal OR fatality OR fatalities OR non-survival* OR Nonsurvival* OR survival* OR lethality OR ""longitudinal studies"" OR ""follow-up studies"" OR ""prospective studies"" OR ""Mass Screening"" OR	

For peer review only

	""Autopsy"")	
#2	"TS=("hiv" OR "hiv-1" OR "hiv-2" OR "hiv1" OR "hiv2" OR hiv infect* OR "human immunodeficiency virus" OR "human immunedeficiency virus" OR "human immuno-deficiency virus" OR "human immune-deficiency virus" OR ((human immun*) AND ("deficiency virus")) OR "acquired immunodeficiency syndrome" OR "acquired immunedeficiency syndrome" OR "acquired immuno-deficiency syndrome" OR "acquired immune-deficiency syndrome" OR ((acquired immun*) AND ("deficiency syndrome")))	
#3	"TS=("Non-Hodgkin lymphoma" OR "Lymphoma, Non Hodgkin" OR "Lymphoma, Small Cleaved-Cell, Diffuse" OR "Lymphoma, Atypical Diffuse Small Lymphoid" OR "Diffuse Small Cleaved-Cell Lymphoma" OR "Diffuse Small Cleaved Cell Lymphoma" OR "Lymphoma, Small Cleaved Cell, Diffuse" OR "Lymphoma, Nonhodgkins" OR "Nonhodgkins Lymphoma" OR "Lymphoma, Non-Hodgkins" OR "Lymphoma, Non Hodgkins" OR "Non-Hodgkins Lymphoma" OR "Non-Hodgkin Lymphoma" OR "Non Hodgkin Lymphoma" OR "Lymphoma, Nonhodgkin's" OR "Lymphoma, Nonhodgkin" OR "Small Cleaved-Cell Lymphoma, Diffuse" OR "Small Cleaved Cell Lymphoma, Diffuse" OR "Lymphoma, Non-Hodgkin's" OR "Lymphoma, Non Hodgkin's" OR "Lymphoma, Diffuse" OR "Diffuse Lymphoma" OR "Diffuse Lymphomas" OR "Sarcoma, Lymphatic" OR "Lymphosarcoma" OR "Lymphosarcomas" OR "Lymphatic Sarcoma" OR "Lymphatic Sarcomas" OR "Lymphoma, Low-Grade" OR "Low-Grade Lymphoma" OR "Low-Grade Lymphomas" OR "Lymphoma, Low Grade" OR "Lymphoma, Mixed-Cell" OR "Lymphoma, Mixed Cell" OR "Mixed-Cell Lymphoma" OR "Mixed Cell Lymphoma" OR "Mixed-Cell Lymphomas" OR "Lymphoma, Mixed" OR "Mixed Lymphoma" OR "Mixed Lymphomas" OR "Lymphoma, Mixed Lymphocytic-Histiocytic"OR "Lymphocytic-Histiocytic Lymphoma, Mixed" OR "Lymphocytic-Histiocytic Lymphomas, Mixed" OR "Lymphoma, Mixed Lymphocytic Histiocytic" OR "Mixed Lymphocytic-Histiocytic Lymphoma" OR "Mixed Lymphocytic-Histiocytic Lymphomas" OR "Lymphoma, Non-Hodgkin, Familial" OR "Lymphoma, Small Noncleaved-Cell" OR "Lymphoma, Small Noncleaved Cell" OR "Small Noncleaved-Cell Lymphoma" OR "Noncleaved-Cell Lymphoma, Small" OR "Small Noncleaved Cell Lymphoma" OR "Small Noncleaved-Cell Lymphomas" OR "Diffuse Undifferentiated Lymphoma" OR "Diffuse Undifferentiated Lymphomas" OR "Lymphoma, Diffuse Undifferentiated" OR "Undifferentiated Lymphoma, Diffuse" OR "Lymphoma, Small Non-Cleaved-Cell" OR "Lymphoma, Small Non Cleaved Cell" OR "Non-Cleaved-Cell Lymphoma, Small" OR "Small Non-Cleaved-Cell Lymphomas" OR "Small Non-Cleaved-Cell Lymphoma" OR "Small Non Cleaved Cell Lymphoma" OR "Lymphoma, Undifferentiated, Diffuse" OR "Lymphoma, Intermediate-Grade" OR "Intermediate-Grade Lymphoma" OR "Intermediate-Grade Lymphomas" OR "Lymphoma,	

For peer review only

	Intermediate Grade" OR "Lymphoma, Undifferentiated" OR "Lymphoma, Pleomorphic" OR "Pleomorphic Lymphoma" OR "Pleomorphic Lymphomas" OR "Undifferentiated Lymphoma" OR "Undifferentiated Lymphomas" OR "Reticulum-Cell Sarcoma" OR "Reticulum-Cell Sarcomas" OR "Sarcoma, Reticulum-Cell" OR "Sarcoma, Reticulum Cell" OR "Reticulum Cell Sarcoma" OR "Reticulum Cell Sarcomas" OR "Reticulosarcoma" OR "Reticulosarcomas" OR "Lymphoma, High-Grade" OR "High-Grade Lymphoma" OR "High-Grade Lymphomas" OR "Lymphoma, High Grade" OR "Lymphoma, Mixed-Cell, Diffuse" OR "Mixed Small and Large Cell Lymphoma, Diffuse" OR "Lymphoma, Mixed Small and Large Cell, Diffuse" OR "Lymphoma, Diffuse, Mixed Lymphocytic-Histiocytic" OR "Lymphoma, Small and Large Cleaved-Cell, Diffuse" OR "Lymphoma, Mixed Cell, Diffuse" OR "Mixed-Cell Lymphoma, Diffuse" OR "Mixed Cell Lymphoma, Diffuse" OR "Diffuse Mixed Small and Large Cell Lymphoma" OR "Diffuse Mixed-Cell Lymphoma" OR "Diffuse Mixed Cell Lymphoma" OR "Diffuse Mixed-Cell Lymphomas" OR "Lymphoma, Diffuse Mixed-Cel" OR "Non-Hodgkin's Lymphoma" OR "Non Hodgkin's Lymphoma" OR "Nonhodgkin's Lymphoma")	
#4	#1 AND #2 AND #3	

For peer review only

Appendix 2. Countries and regions by UNAIDS

UNAIDS Regions	Country	ISO Code	Unaids Regions	Country	ISO Code
**Global**	~	03M49WLD	**Latin America**	Argentina	ARG
**Asia and the Pacific**	Afghanistan	AFG		Bolivia	BOL
	Australia	AUS		Brazil	BRA
	Bangladesh	BGD		Chile	CHL
	Bhutan	BTN		Colombia	COL
	Brunei	BRN		Costa Rica	CRI
	Cambodia	KHM		Ecuador	ECU
	China	CHN		El Salvador	SLV
	South Korea	PRK		Guatemala	GTM
	Micronesia	FSM		Honduras	HND
	Fiji	FJI		Mexico	MEX
	India	IND		Nicaragua	NIC
	Indonesia	IDN		Panama	PAN
	Japan	JPN		Paraguay	PRY
	Kiribati	KIR		Peru	PER
	Laos	LAO		Uruguay	URY
	Malaysia	MYS		Venezuela	VEN
	Maldives	MDV	**Middle East and North Africa**	Algeria	DZA
	Marshall Islands	MHL		Bahrain	BHR
	Mongolia	MNG		Djibouti	DJI
	Myanmar	MMR		Egypt	EGY
	Nauru	NRU		Iraq	IRQ

For peer review only

	Nepal	NPL		Iran	IRN
	New Zealand	NZL		Jordan	JOR
	Pakistan	PAK		Kuwait	KWT
	Palau	PLW		Lebanon	LBN
	Papua New Guinea	PNG		Libya	LBY
	Philippines	PHL		Morocco	MAR
	North Korea	KOR		Oman	OMN
	Samoa	WSM		Qatar	QAT
	Singapore	SGP		Saudi Arabia	SAU
	Solomon Islands	SLB		Somalia	SOM
	Sri Lanka	LKA		Sudan	SDN
	Thailand	THA		Syria	SYR
	Timor-Leste	TLS		Tunisia	TUN
	Tonga	TON		United Arab Emirates	ARE
	Tuvalu	TUV		Yemen	YEM
	Vanuatu	VUT	**Western and central Africa**	Benin	BEN
	Vietnam	VNM		Burkina Faso	BFA
**Caribbean**	Antigua	ATG		Burundi	BDI
	Bahamas	BMH		Ivory Coast	CIV
	Barbados	BRB		Cameroon	CMR
	Belize	BLZ		Cape Verde	CPV
	Cuba	CUB		Central African Republic	CAF
	Dominica	DMA		Chad	TCD
	Dominican Republic	DOM		Republic of Congo	COG
	Grenada	GRD		Democratic Republic of	COD

For peer review only

				the Congo	
	Guyana	GUY		Equatorial Guinea	GNQ
	Haiti	HTI		Gabon	GAB
	Jamaica	JAM		Gambia	GMB
	Saint Kitts	KNA		Ghana	GHA
	Saint Lucia	LCA		Guinea	GIN
	Saint Vincent	VCT		Guinea-Bissau	GNB
	Suriname	SUR		Liberia	LBR
	Trinidad	TTO		Mali	MLI
**Eastern and southern Africa**	Angola	AGO		Mauritania	MRT
	Botswana	BWA		Niger	NER
	Comoros	COM		Nigeria	NGA
	Eritrea	ERI		Sao Tome and Principe	STP
	Swaziland	SWZ		Senegal	SEN
	Ethiopia	ETH		Sierra Leone	SLE
	Kenya	KEN		Togo	TGO
	Lesotho	LSO	**Western and central Europe and North America**	Andorra	AND
	Madagascar	MDG		Austria	AUT
	Malawi	MWI		Belgium	BEL
	Mauritius	MUS		Bulgaria	BGR
	Mozambique	MOZ		Canada	CAN
	Namibia	NAM		Croatia	HRV
	Rwanda	RWA		Cyprus	CYP
	Seychelles	SYC		Czech Republic	CZE
	South Africa	ZAF		Denmark	DNK

For peer review only

	South Sudan	SSD		Estonia	EST
	Uganda	UGA		Finland	FIN
	Tanzania	TZA		France	FRA
	Zambia	ZMB		Germany	DEU
	Zimbabwe	ZWE		Greece	GRC
**Eastern Europe and central Asia**	Albania	ALB		Hungary	HUN
	Armenia	ARM		Iceland	ISL
	Azerbaijan	AZE		Ireland	IRL
	Belarus	BLR		Israel	ISR
	Bosnia and Herzegovina	BIH		Italy	ITA
	Georgia	GEO		Latvia	LVA
	Kazakhstan	KAZ		Lithuania	LTU
	Kyrgyzstan	KGZ		Luxembourg	LUX
	Montenegro	MNE		Netherlands	NLD
	Moldova	MDA		Norway	NOR
	Russia	RUS		Poland	POL
	Tajikistan	TJK		Portugal	PRT
	Micronesia (Federated States of)	MKD		Romania	ROU
	Turkmenistan	TKM		Serbia	SRB
	Ukraine	UKR		Slovakia	SVK
	Uzbekistan	UZB		Slovenia	SVN
				Spain	ESP
				Sweden	SWE
				Switzerland	CHE

For peer review only

				Turkey	TUR
				UK	GBR
				USA	USA

For peer review only

PRISMA-P (preferred reporting items for systematic review and meta-analysis protocols) 2015 checklist: recommended items to address in a systematic review protocol

Section and topic	Item No	Checklist item	Page number
**Administrative information**			
Title:			
Identification	1a	Identify the report as a protocol of a systematic review	1
Update	1b	If the protocol is for an update of a previous systematic review, identify as such	NA
Registration	2	If registered, provide the name of the registry (such as PROSPERO) and registration number	3 & 5
Authors:			
Contact	3a	Provide name, institutional affiliation, e-mail address of all protocol authors; provide physical mailing address of corresponding author	1
Contributions	3b	Describe contributions of protocol authors and identify the guarantor of the review	11
Amendments	4	If the protocol represents an amendment of a previously completed or published protocol, identify as such and list changes; otherwise, state plan for documenting important protocol amendments	NA
Support:			
Sources	5a	Indicate sources of financial or other support for the review	12
Sponsor	5b	Provide name for the review funder and/or sponsor	12
Role of sponsor or funder	5c	Describe roles of funder(s), sponsor(s), and/or institution(s), if any, in developing the protocol	12
**Introduction**			
Rationale	6	Describe the rationale for the review in the context of what is already known	4 & 5
Objectives	7	Provide an explicit statement of the question(s) the review will address with reference to participants, interventions, comparators, and outcomes (PICO)	5
**Methods**			
Eligibility criteria	8	Specify the study characteristics (such as PICO, study design, setting, time frame) and report characteristics (such as years considered, language, publication status) to be used as criteria for eligibility for the review	6
Information sources	9	Describe all intended information sources (such as electronic databases, contact with study authors, trial registers or other grey literature sources) with planned dates of coverage	6 & 9
Search strategy	10	Present draft of search strategy to be used for at least one electronic database, including planned limits, such that it could be repeated	18 & 25

Study records:			
Data management	11a	Describe the mechanism(s) that will be used to manage records and data throughout the review	7
Selection process	11b	State the process that will be used for selecting studies (such as two independent reviewers) through each phase of the review (that is, screening, eligibility and inclusion in meta-analysis)	7 & 8
Data collection process	11c	Describe planned method of extracting data from reports (such as piloting forms, done independently, in duplicate), any processes for obtaining and confirming data from investigators	8
Data items	12	List and define all variables for which data will be sought (such as PICO items, funding sources), any pre-planned data assumptions and simplifications	8 & 15
Outcomes and prioritization	13	List and define all outcomes for which data will be sought, including prioritization of main and additional outcomes, with rationale	6
Risk of bias in individual studies	14	Describe anticipated methods for assessing risk of bias of individual studies, including whether this will be done at the outcome or study level, or both; state how this information will be used in data synthesis	9
Data synthesis	15a	Describe criteria under which study data will be quantitatively synthesised	6
	15b	If data are appropriate for quantitative synthesis, describe planned summary measures, methods of handling data and methods of combining data from studies, including any planned exploration of consistency (such as I^2, Kendall's τ)	8 & 9
	15c	Describe any proposed additional analyses (such as sensitivity or subgroup analyses, meta-regression)	8 & 9
	15d	If quantitative synthesis is not appropriate, describe the type of summary planned	8 & 9
Meta-bias(es)	16	Specify any planned assessment of meta-bias(es) (such as publication bias across studies, selective reporting within studies)	9
Confidence in cumulative evidence	17	Describe how the strength of the body of evidence will be assessed (such as GRADE)	8

From: Shamseer L, Moher D, Clarke M, Ghersi D, Liberati A, Petticrew M, Shekelle P, Stewart L, PRISMA-P Group. Preferred reporting items for systematic review and meta-analysis protocols (PRISMA-P) 2015: elaboration and explanation. BMJ. 2015 Jan 2;349(jan02 1):g7647.

Global and regional burden estimation of HIV-associated Non-Hodgkin Lymphoma: a meta-analysis and modeling analysis protocol

Journal:	*BMJ Open*
Manuscript ID	bmjopen-2023-075933.R1
Article Type:	Protocol
Date Submitted by the Author:	07-Nov-2023
Complete List of Authors:	Chen, Yan; Southwest Medical University, School of Public Health; University of Electronic Science and Technology of China, Department of Health Management Center & Institute of Health Management<br>Sun, Ping; University of Electronic Science and Technology of China, Department of Health Management Center & Institute of Health Management<br>Sun, Zhaochen; Southwest Medical University, School of Public Health; University of Electronic Science and Technology of China, Department of Health Management Center & Institute of Health Management<br>Liu, Yuping; University of Electronic Science and Technology of China, Department of Health Management Center & Institute of Health Management<br>Wan, Zhengwei; University of Electronic Science and Technology of China, Department of Health Management Center & Institute of Health Management<br>Ye, Yunli; Southwest Medical University, School of Public Health
**Primary Subject Heading**:	Global health
Secondary Subject Heading:	Oncology, HIV/AIDS, Public health
Keywords:	HIV & AIDS < INFECTIOUS DISEASES, Lymphoma < ONCOLOGY, Meta-Analysis, PUBLIC HEALTH

SCHOLARONE™ Manuscripts

Global and regional burden estimation of HIV-associated Non-Hodgkin Lymphoma: a meta-analysis and modeling analysis protocol

Yan Chen[1,2], Ping Sun[2,3], Zhaochen Sun[1,2], Yuping Liu[2,3], Zhengwei Wan[2,3#], Yunli Ye[1#]

[1] School of Public Health, Southwest Medical University, Luzhou, China.

[2] Department of Health Management Center & Institute of Health Management, Sichuan Provincial People's Hospital, University of Electronic Science and Technology of China, Chengdu, China.

[3]Chinese Academy of Sciences Sichuan Translational Medicine Research Hospital, Chengdu, China.

Email address:

Yan Chen: chenyan122071@163.com

Ping Sun: sunping612@foxmail.com

Zhaochen Sun: 13585928551@163.com

Yuping Liu: 18981838972@163.com

[#] Corresponding authors

Prof. Yunli Ye

Address: No.1 First Section, Xianglin Rd, Longmatan Dist, Luzhou, China;

E-mail: wushuangyewu@163.com;

Tel: +86 18982785179.

Dr. Zhengwei Wan

Address: No. 32 West Second Section, First Ring Rd., Qingyang Dist., Chengdu, China;

E-mail: 18715799366@163.com;

Tel: +86 18715799366.

For peer review only

For peer review only - http://bmjopen.bmj.com/site/about/guidelines.xhtml

ABSTRACT

Introduction Human immunodeficiency virus (HIV) infection is one of the complex etiologies of Non-Hodgkin lymphoma (NHL). However, the contribution of HIV to the disease burden of NHL across times and regions has yet to be comprehensively reported and quantified. Thus, the purpose of this study is to evaluate the relative risk of NHL in individuals with HIV-infection compared to those without HIV-infection by performing a comprehensive meta-analysis. Additionally, we intend to further estimate quantitatively the degree of HIV contributing to the burden of NHL with population attributable fraction (PAF) modeling analysis.

Methods and analysis This study will screen a mass of records searched from electronic databases (PubMed, EMBASE, Cochrane Library, and Web of Science). The main outcomes of this review are specific effect values and corresponding 95% confidence intervals (95% CI) for NHL among persons with HIV compared to those without HIV to quantify the association between HIV-infection and NHL. After quality assessment and data extraction, a meta-analysis will be performed to evaluate the pooled risk ratio (RR) of NHL for HIV-infected population. Furthermore, the pooled RR value will be subjected to PAF modeling analysis, combined with the number of age-specific disability-adjusted life years (DALYs) and HIV prevalence data of 15 years old and above from 1990 to 2019, at global, regional, and country levels. We will mainly calculate the PAF, HIV-associated DALYs number and age-standardized rates (ASRs) to quantify the burden of HIV-associated NHL.

Ethics and dissemination Our study is based on published articles thus the ethnic approval is not essential. In addition, we intend to publish the results on peer-reviewed journal for better discussion. We believe that conducting research on estimating the global burden of NHL can provide valuable insights for developing targeted prevention and control strategies, thereby achieving significant benefits.

For peer review only - http://bmjopen.bmj.com/site/about/guidelines.xhtml

PROSPERO registration number: CRD 42023404150.

Keywords: HIV, non-Hodgkin Lymphoma, meta-analysis, population attributable fraction, global burden

Strengths and limitations of this study

1. The study will include meta-analysis and population attributable fraction estimation modeling analysis, which aiding in the formulation of precise prevention and control policies.

2. The quality of included studies will be assessed using an 11-iterm checklist recommended by the Agency for Healthcare Research and Quality (AHRQ).

3. The dissemination of the results may be limited because of the language restriction.

4. The pooled RR value will be applied to population attributable fraction modeling across global and regional areas, which may lead to bias.

For peer review only - http://bmjopen.bmj.com/site/about/guidelines.xhtml

Introduction

Non-Hodgkin lymphoma (NHL) is a common lymphoid tissues neoplasm that originates from B cell precursors, mature B cells, T cell precursors, and mature T cells.[1] According to the World Health Organization (WHO) classification, the most common NHL in Western countries are diffuse large B cell lymphoma (DLBCL), accounting for around 31% of adult cases, follicular lymphoma (FL) (22%) and Burkit lymphoma (BL) (2%).[2, 3] Based on the GLOBOCAN database of WHO's International Agency for Research on Cancer (IARC), which includes 36 types of cancer worldwide in 2020, NHL accounted for 2.8% of total cancers with 544,352 new cases, and 2.6% of total cancer-related deaths with 259,793 new deaths, ranking NHL within the top 10 cancers for both males (ranked 8th) and females (ranked 10th).[4]

The etiology of NHL exhibits significant complexity. Published articles have identified various potential causes of NHL, including but not limited to immunosuppression, viral infection (e.g., human immunodeficiency virus, HIV), family factor, occupational and environmental exposures, organ transplantation and autoimmune diseases.[5, 6, 7] Due to its association with immunosuppression, NHL is regarded as a defining event of acquired immune deficiency syndrome (AIDS),[6] thus establishing a strong connection between HIV and NHL. Based on a population-based review encompassing the United States, Australia and Italy, the risk of NHL among individuals with AIDS, when compared to the general population, ranged from 15-fold for low-grade NHL to 400-fold for high-grade NHL.[8] In addition, certain subtypes of NHL have been found to be associated with HIV infection. Another multicohort study conducted in Europe revealed a higher incidence of NHL among individuals with HIV, regardless of whether they received combination antiretroviral therapy (cART) treatment.[9] Although the introduction of cART in the late 1990s has significantly reduced the incidence of NHL,[10, 11] such as the United States alone recorded 74,680 new NHL cancer diagnoses and 19,910 NHL-related deaths in 2018. However, the high burden of HIV infection in South

For peer review only - http://bmjopen.bmj.com/site/about/guidelines.xhtml

Africa has contributed to an increased incidence of plasmablastic lymphoma (PBL), which is a subtype of NHL.[6, 12]

Because of the significant variation of HIV-associated NHL burden, however, the contribution of HIV to the disease burden of NHL across times and regions has yet to be comprehensively reported and quantified. This study aims to perform a comprehensive meta-analysis to evaluate the relative risk of NHL in individuals with HIV-infection compared to those without HIV exposure. Additionally, we intend to further estimate quantitatively the degree of HIV contributing to the burden of NHL using population attributable fraction (PAF) modeling analysis. The anticipated outcome of this study is expected to provide valuable insights for the development of targeted prevention and control strategies for NHL.

For peer review only

Objectives

This study aims to resolve questions:

1. Although NHL is considered an AIDS-defining disease, no meta-analysis has quantified the pooled risk ratio of NHL in HIV-infected individuals compared to those without HIV infection.

2. The contribution of HIV to the disease burden of NHL across times and regions has yet been comprehensively reported and quantified.

Methods and analysis

Our study will comprise two essential components as follows: 1) meta-analysis to evaluate relative risk of NHL for HIV-infected population; 2) PAF modeling analysis to assess HIV-associated burden of NHL.

1. Meta-analysis to assess the relative risk of NHL in HIV-infected population compared to people without HIV-infection

1.1 Registration

This protocol will follow to the guidelines of Preferred Reporting Items for Systematic reviews and Meta-Analyses for Protocols 2015 (PRISMA-P 2015)[13] and

For peer review only - http://bmjopen.bmj.com/site/about/guidelines.xhtml

has registered in the PROSPERO International prospective register of systematic reviews (https://www.crd.york.ac.uk/PROSPERO/) (CRD 42023404150).

1.2 Outcomes definition, inclusion criteria and exclusion criteria

The main outcomes of this review are specific effect values and corresponding 95% confidence intervals (95% CI) for NHL among persons with HIV compared to those without HIV to quantify the association between HIV and NHL. Effect values that will be considered are risk ratio (RR), odds ratio (OR) and standardized incidence rate (SIR).

For inclusion criteria, our target design types are cohort studies, case-control studies or cross-sectional studies with effective control group. These studies need to expound the association between HIV and NHL using target effect value. In addition, eligible studies should provide detailed participant information, including the total number of participants, the number of participants in case/exposure and control/non-exposure groups, as well as the quantity of NHL cases in case/exposure group.

For exclusion criteria, we will exclude records whose type are review, opinion pieces, poster presentations, letters, case reports or case series. If there is no effective effect value about association between HIV and NHL or it is not target study population, this record will be also excluded. In addition, when there is population overlap in multiple articles, we will choose the one with larger study size and better article quality. As regards duplicate records, we will exclude incomplete or earlier period version.

1.3 Search strategy

We will search for English articles without restrictions of date or setting from four electronic databases: PubMed, Excerpta Medica database (EMBASE), Cochrane Library, and Web of Science up to 31 March 2023 with keywords non-Hodgkin lymphoma, NHL, Burkitt lymphoma, BL, diffuse large B-cell lymphoma, DLBCL,

For peer review only

human immunodeficiency virus and HIV (i.e., NHL, subtypes of NHL, HIV, and their equivalent terms). In addition, references of eligible records will be also searched to provide a more comprehensive searching strategy. The full electronic search strategies are included in the additional information (Appendix 1).

1.4 Data management and study selection

All retrieved records resulting from the search strings will be imported into Endnote X9.2 for the purposes of organizing and screening eligible studies. Research members will enact selection procedures and inclusion criteria in advance all together.

Step 1: Duplicate record and article type screening

Two reviewers will initially utilize the built-in function of Endnote X9.2 to eliminate duplicate records. Subsequently, they will employ keyword searches within the software to exclude review and case reports that are not relevant to the type of literature in this study. Due to the limitation of incomplete duplication checking function, certain duplicate records still need to be removed manually. Thus, we leverage the advantage of two-person independent operation to reduce the possibility of omissions. Following the manual deletion process, the two reviewers will cross-check the exclusion results to ensure the highest level of completeness in the final record selection.

Step 2: Title screening

Next, two authors will review the titles of the remaining records, excluding those that do not involve HIV population or outcome of NHL. Moreover, these records that contain mice, mouse, review, case report, drug trial or other non-target exposure and outcome will also be excluded based on our target study types and study subject.

Step 3: Abstract screening

If any record contains the target effect values and accords with desired causal association in the abstract, it will proceed to the next screening phase. If there is any

For peer review only - http://bmjopen.bmj.com/site/about/guidelines.xhtml

uncertainty, it is necessary to read the full text.

Step 4: Full text screening

The full texts of the provisionally selected records will be downloaded from databases. Two authors will read full text throughout to reconfirm and extract data that can be utilized for statistical analyses. Articles will be ultimately included if they meet the eligibility criteria. Throughout the screening process, any discrepancies or conflicts will be solved by consensus or arbitration from a third reviewer. Additionally, meticulous records will be maintained for each step of the screening processes, which will facilitate the creation of a comprehensive flow chart and the composition of formal article.

1.5 Data extraction

Two authors evaluate independently all retrieved articles by title, abstract, and full text according to the above-mentioned inclusion and exclusion criteria. Solution of discrepancies or conflicts will be same as the part of study selection. EndNote X9.2 will be used to manage the citations and extract data. The following data will be considered to extract (Table).

1.6 Quality assessment

All included studies will be assessed independently by two authors using an 11-item checklist recommended by the Agency for Healthcare Research and Quality (AHRQ) (https://www.wjx.cn/m/85284180.aspx). In this checklist, an item would be scored as '0' if the answer is 'NO' or 'UNCLEAR', and '1' if the answer is 'YES'. The determination of study quality will be complied with correspondence relationships as follows: low quality (0-3), moderate quality (4-7), high quality (8-11).[14] The judgement of studies' risk level will accord to the result of study quality assessment to divide into high, middle and low risk level corresponding to low, middle, high quality in sequence. Any disagreements between the two authors will be resolved according to the procedures outlined in the study selection process.

For peer review only - http://bmjopen.bmj.com/site/about/guidelines.xhtml

1.7 Statistical analysis

Overall statistical analyses and calculation will be completed by the R software (version 4.2.2). Firstly, we will calculate a pooled RR and the corresponding 95% confidence intervals (95% CI) using function 'metagen' included in package 'meta' to quantitatively estimate the association between HIV and NHL. Then the heterogeneity will be assessed using the Chi-squared test on Cochrane's Q statistic, and the quantitation of heterogeneity will be measured by the Higgins's I^2 value presented as a percentage.[15] When the I^2 value exceeds 50% we will choose random effects model and fixed effects model will be used when I^2 value is less than or equal to 50%. If we can obtain sufficient data, subgroup analyses will be utilized to find the potential source of heterogeneity, including NHL subtypes, study period, population age, HIV mortality rate, study conducted region, study design types, setting of study (multi-center or single center), article quality risk level.

Furthermore, a meta-regression will be performed to ensure whether these factors can explain the heterogeneity and the degree to which they account for it. Regarding sensitivity analysis, we intend to analysis influence of single study by omitting study one-by-one and different risk levels. Furthermore, we will also use funnel plot and Egger's linear regression (if outcomes with more than 10 individual studies) to find potential publication bias from these articles. If publication bias is detected, we will address it using the trim-and-fill method to adjust for the bias.

2. Population attributable fraction modeling analysis for HIV-associated burden of NHL estimation

2.1 Information sources

The number of age-specific disability-adjusted life years (DALYs) will be obtained from the Global Health Data Exchange (GHDx) query tool (https://vizhub.healthdata.org/gbd-results/) in 5 years-old intervals. We will download national or territorial HIV prevalence of 15 years-old and above from 1990 to 2019

For peer review only - http://bmjopen.bmj.com/site/about/guidelines.xhtml

from the Joint United Nations Programme on HIV/AIDS (UNAIDS) (https://aidsinfo.unaids.org/). The criteria for dividing countries and regions will use UNAIDS classifications and ISO codes alpha-3 (Appendix 2).

2.2 Population attributable fraction modeling implementation

Once we have obtained the pooled RR value from the meta-analysis, we will proceed with the PAF modeling to estimate the burden of HIV-associated NHL (including HIV-related DALYs， and age-standardized DALYs rate) according to the following steps.

Step 1: Population attributable fraction (PAF, %) calculation

We will calculate PAF combined with HIV prevalence data and pooled RR, with the following assumptions: 1) the pooled RR is applicable across the global, regions and all countries; 2) we ignore various confounding biases between primary studies, which may cause slight deviation in our estimation; 3) we assume the pooled RR fits log normal distribution and beta distribution for the HIV prevalence, then we calculate 10,000 samples of PAF to obtain uncertainty intervals (2.5%, 97.5%) by R software. In practice, if eligible articles cover eight UNAIDS regions and have information on our planned subgroup analyses with sufficient data, we can calculate respective specific PAF such as NHL subtype, period, or age-specific RR. We will estimate the PAF using the Levin's formula [16]:

$$PAF = \frac{HIV\ prevalence * (RR - 1)}{1 + HIV\ prevalence * (RR - 1)}$$

Figure 1 illustrates the calculation steps of PAF, using South Africa as an example.

Step 2: Estimation of NHL disability-adjusted life years (DALYs) related to HIV

We calculate age standardized incidence rates (ASRs) per 100,000 population of all-cause DALYs and corresponding 95% confidence intervals by direct method, based on the age-specific all-cause NHL DALYs and world standard population reported in the GBD 2019. Formulas for calculating the ASR as below:

For peer review only - http://bmjopen.bmj.com/site/about/guidelines.xhtml

1)

$$Crude\ Rate = \frac{Case}{Population}$$

2)

$$Proportion = \frac{Age\ Group}{\sum Age\ Group}$$

3) for each age group: $Adjust\ Rate = Crude\ Rate * Proportion$

4) for ⩾15 years old: $Adjust\ Rate = 100000 * sum\ (Adjust\ Rates)$

Then we assume that both number of DALYs and ASRs fit log normal distribution and we calculate 10,000 samples of HIV-associated DALYs and ASRs to obtain uncertainty intervals (2.5%, 97.5%) by R software. We estimate the HIV-associated DALYs number and ASRs using formulas as below:

1) $DALY_{\text{attributed to HIV}} = All\_cause\ DALY * PAF$

2) $DALY\ ASR_{\text{attributed to HIV}} = All\_cause\ DALYs\ ASR * PAF$

Figure 2 shows the calculation steps of the HIV-associated NHL DALYs for South Africa.

3. Patient and public involvement

No patient involved.

Discussion

The incidence rate of NHL exhibited a dramatic increase starting around 1970, and despite experiencing a plateau in between, it still recorded over 500 thousand of new cases in 2020.[4, 17] The complex etiology of NHL, coupled with the accelerated aging process of global population, is expected to further contributed to an increase of patients and deaths number for NHL.[6, 17] According to the UNAIDS, there are approximately 4000 new HIV infections reported daily, with 1100 of those are young people (aged 15 to 24 years old).[18] Given to the close relationship between HIV and NHL, it is crucial to be concerned about the trajectory of the burden of HIV-associated NHL.

For peer review only - http://bmjopen.bmj.com/site/about/guidelines.xhtml

Our study aims to investigate the risk of NHL in individuals with HIV infection compared to those without HIV, and to quantify the contribution of HIV to incidence of NHL. To ensure the readability of the literature, we will only include articles published in English, which may limit the generalizability of our findings. In addition, it is also a limitation that we assume the pooled RR is applicable across global and regional areas. This is possible to lead to potential bias. Regarding the dissemination of our results, we will make every effort to publish them in a peer-reviewed journal as an academic article and present our findings at relevant field meetings. Since this review is based on published data, an ethical review is not required. Considering the global impact of HIV-associated NHL, we believe that conducting research on estimating the global burden of the disease can provide valuable insights for developing targeted prevention and control strategies, thereby achieving significant benefits.

Authors' contributions YC, YLY, ZWW, PS and ZCS designed the study protocol. YC and ZCS prepared the first draft. YLY, ZWW and YPL reviewed and revised the first draft. All authors read and approved the final manuscript.

Funding statement This work was supported by [Science Foundation for Young Scholars of Sichuan Provincial People's Hospital] grant number [NO. 2022QN18] and [Subject Founds of Health Care for Cadres of Sichuan Province] grant number [NO. 2022-220].

Competing interests The authors declared that they had no competing interests.

Patient consent for publication Not required.

References

1. Sapkota S, Shaikh H: **Non-Hodgkin Lymphoma**. In: *StatPearls.* Treasure Island (FL) ineligible companies; 2023.

For peer review only - http://bmjopen.bmj.com/site/about/guidelines.xhtml

2. Swerdlow SH, Campo E, Pileri SA, Harris NL, Stein H, Siebert R, Advani R, Ghielmini M, Salles GA, Zelenetz AD *et al*: **The 2016 revision of the World Health Organization classification of lymphoid neoplasms**. *Blood* 2016, **127**(20):2375-2390. doi: 10.1182/blood-2016-01-643569
3. Thandra KC, Barsouk A, Saginala K, Padala SA, Barsouk A, Rawla P: **Epidemiology of Non-Hodgkin's Lymphoma**. *Med Sci (Basel)* 2021, **9**(1). doi: 10.3390/medsci9010005
4. Sung H, Ferlay J, Siegel RL, Laversanne M, Soerjomataram I, Jemal A, Bray F: **Global Cancer Statistics 2020: GLOBOCAN Estimates of Incidence and Mortality Worldwide for 36 Cancers in 185 Countries**. *CA Cancer J Clin* 2021, **71**(3):209-249. doi: 10.3322/caac.21660
5. Muller AM, Ihorst G, Mertelsmann R, Engelhardt M: **Epidemiology of non-Hodgkin's lymphoma (NHL): trends, geographic distribution, and etiology**. *Ann Hematol* 2005, **84**(1):1-12. doi: 10.1007/s00277-004-0939-7
6. Bispo JAB, Pinheiro PS, Kobetz EK: **Epidemiology and Etiology of Leukemia and Lymphoma**. *Cold Spring Harb Perspect Med* 2020, **10**(6). doi: 10.1101/cshperspect.a034819
7. Rana I, Dahlberg S, Steinmaus C, Zhang L: **Benzene exposure and non-Hodgkin lymphoma: a systematic review and meta-analysis of human studies**. *Lancet Planet Health* 2021, **5**(9):e633-e643. doi: 10.1016/S2542-5196(21)00149-2
8. Dal Maso L, Franceschi S: **Epidemiology of non-Hodgkin lymphomas and other haemolymphopoietic neoplasms in people with AIDS**. *Lancet Oncol* 2003, **4**(2):110-119. doi: 10.1016/s1470-2045(03)00983-5
9. Collaboration of Observational HIVERESG, Bohlius J, Schmidlin K, Costagliola D, Fatkenheuer G, May M, Caro-Murillo AM, Mocroft A, Bonnet F, Clifford G *et al*: **Incidence and risk factors of HIV-related non-Hodgkin's lymphoma in the era of combination antiretroviral therapy: a European multicohort study**. *Antivir Ther* 2009, **14**(8):1065-1074. doi: 10.3851/IMP1462
10. Jacobson LP, Yamashita TE, Detels R, Margolick JB, Chmiel JS, Kingsley LA, Melnick S, Munoz A: **Impact of potent antiretroviral therapy on the incidence of Kaposi's sarcoma and non-Hodgkin's lymphomas among HIV-1-infected individuals. Multicenter AIDS Cohort Study**. *J Acquir Immune Defic Syndr* 1999, **21 Suppl 1**:S34-41.
11. Polesel J, Clifford GM, Rickenbach M, Dal Maso L, Battegay M, Bouchardy C, Furrer H, Hasse B, Levi F, Probst-Hensch NM *et al*: **Non-Hodgkin lymphoma incidence in the Swiss HIV Cohort Study before and after highly active antiretroviral therapy**. *AIDS* 2008, **22**(2):301-306. doi: 10.1097/QAD.0b013e3282f2705d
12. Vaughan J, Perner Y, Mayne E, Wiggill T: **Plasmablastic lymphoma in Johannesburg, South Africa, in the era of widescale antiretroviral therapy use**. *HIV Med* 2021, **22**(3):225-230. doi: 10.1111/hiv.12965
13. Moher D, Shamseer L, Clarke M, Ghersi D, Liberati A, Petticrew M, Shekelle P, Stewart LA, Group P-P: **Preferred reporting items for systematic review and meta-analysis protocols (PRISMA-P) 2015 statement**. *Syst Rev* 2015, **4**(1):1. doi: 10.1186/2046-4053-4-1
14. Hu J, Dong Y, Chen X, Liu Y, Ma D, Liu X, Zheng R, Mao X, Chen T, He W: **Prevalence of suicide attempts among Chinese adolescents: A meta-analysis of cross-sectional studies**. *Compr Psychiatry* 2015, **61**:78-89. doi: 10.1016/j.comppsych.2015.05.001
15. Higgins JP, Thompson SG: **Quantifying heterogeneity in a meta-analysis**. *Stat Med* 2002, **21**(11):1539-1558. doi: 10.1002/sim.1186
16. Khosravi A, Nielsen RO, Mansournia MA: **Methods matter: population attributable fraction (PAF) in sport and exercise medicine**. *Br J Sports Med* 2020, **54**(17):1049-1054. doi:

For peer review only - http://bmjopen.bmj.com/site/about/guidelines.xhtml

10.1136/bjsports-2020-101977

17. Skrabek P, Turner D, Seftel M: **Epidemiology of non-Hodgkin lymphoma**. *Transfus Apher Sci* 2013, **49**(2):133-138. doi: 10.1016/j.transci.2013.07.014

18. UNAIDS. IN DANGER: UNAIDS Global AIDS Update 2022: UNAIDS; 2022 [cited 2023 14 May]. Available from: https://www.unaids.org/en/resources/documents/2022/in-danger-global-aids-update.

For peer review only

For peer review only - http://bmjopen.bmj.com/site/about/guidelines.xhtml

Table. Data to be extracted from eligible articles.

Category	Data
Publication details	Author, publication year
Type of design	Cohort study, CCS, or cross-sectional study with effective control group
Information of study	Time period of study, NHL subtype, HIV mortality rate, median patient age ,country and region of research conducted, study setting (multi-center or single center), disease classification criterion, data source or name of cohort registered
Effect values	RR, OR and SIR and their corresponding 95% CI
Study participants	Total number of HIV patients, total number of control individuals, number of NHL in the case group and number of control group

Abbreviations: CCS-Case-control study; RR-Risk ratio; OR-Odds ratio; SIR-Standardized incidence rate; CI-Confidence intervals; HIV-Human immunodeficiency virus; NHL-Non-Hodgkin lymphoma

Figure legends:

Figure 1. Calculation steps of PAF for South Africa

Schematic diagram of PAF calculation using South Africa.

Abbreviations: PAF-Population attributable fraction

Figure 2. Calculation steps of the HIV-associated NHL DALYs for South Africa

Schematic diagram of calculation for HIV-associated NHL DALYs using South Africa.

Abbreviations: HIV-Human immunodeficiency virus; NHL-Non-Hodgkin lymphoma; DALY-Disability-adjusted life year

For peer review only - http://bmjopen.bmj.com/site/about/guidelines.xhtml

South Africa in 2000:

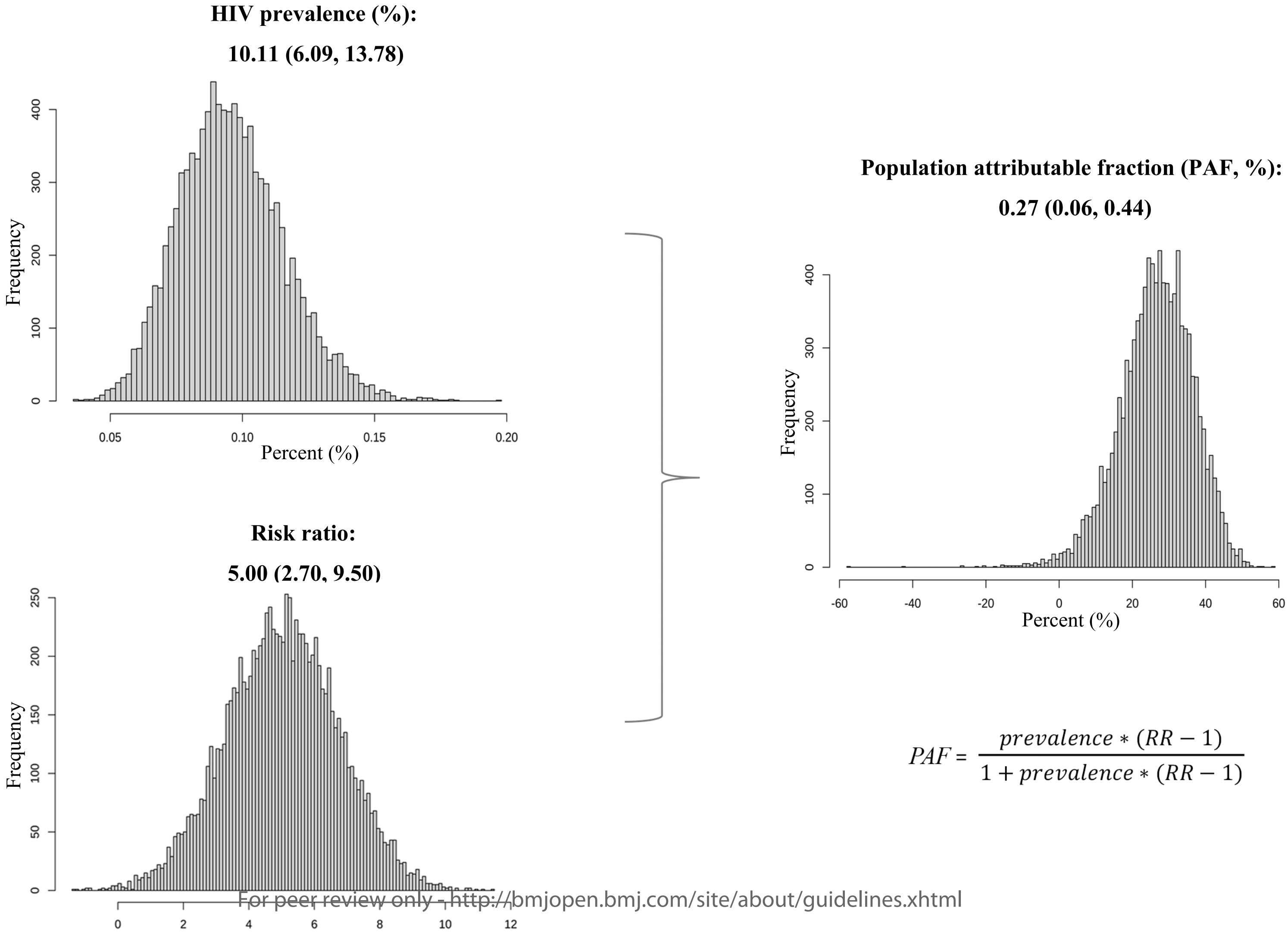

$$PAF = \frac{prevalence * (RR - 1)}{1 + prevalence * (RR - 1)}$$

For peer review only - http://bmjopen.bmj.com/site/about/guidelines.xhtml

South Africa in 2000:

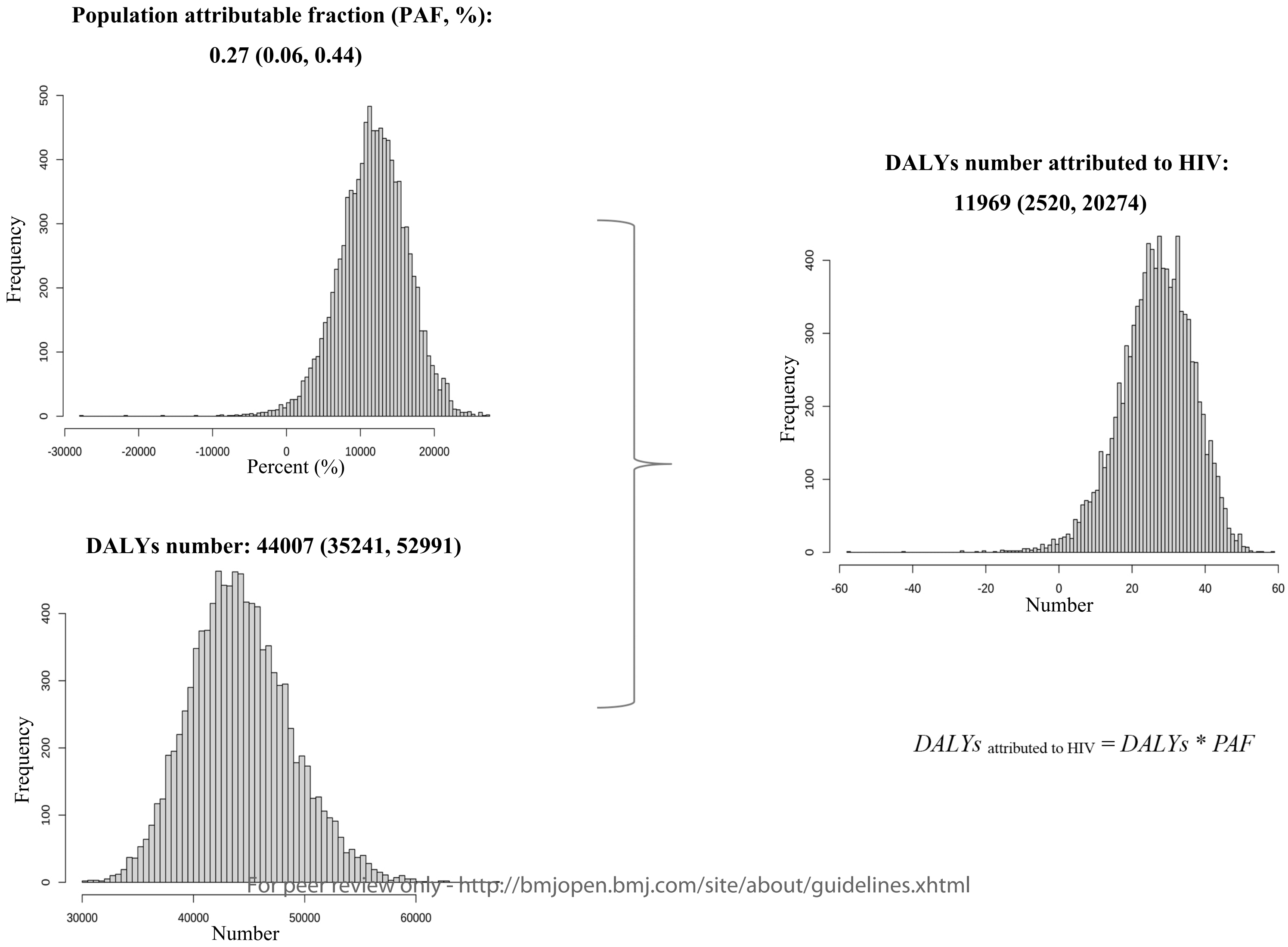

Appendix

Appendix 1. Electronic databases search strategies in PubMed, Embase, Cochrane Library and Web of Science

Pubmed

Set	Search	Results
#1	"lymphoma, non hodgkin"[MeSH Terms]	
#2	"hiv"[Title/Abstract] OR "hiv-1"[Title/Abstract] OR "hiv 1"[Title/Abstract] OR "hiv 2"[Title/Abstract] OR "hiv-2"[Title/Abstract] OR "human immunodeficiency virus"[Title/Abstract] OR "human immuno deficiency virus"[Title/Abstract] OR "human immunedeficiency virus"[Title/Abstract] OR "human immune deficiency virus"[Title/Abstract] OR "acquired immunodeficiency syndrome"[MeSH Terms] OR "acquired immunodeficiency syndrome"[Title/Abstract] OR "acquired immuno-deficiency syndrome"[Title/Abstract]OR "acquired immunedeficiency syndrome"[Title/Abstract]	
#3	"lymphoma non hodgkin"[Title/Abstract] OR (("Lymphoma"[MeSH Terms] OR "Lymphoma"[All Fields] OR "Lymphomas"[All Fields] OR "lymphoma s"[All Fields]) AND "small cleaved cell diffuse"[Title/Abstract]) OR ((("Lymphoma"[MeSH Terms] OR "Lymphoma"[All Fields] OR "Lymphomas"[All Fields] OR "lymphoma s"[All Fields]) AND ("atypic"[All Fields] OR "atypical"[All Fields] OR "atypicalities"[All Fields] OR "atypicality"[All Fields] OR "atypically"[All Fields] OR "atypicals"[All Fields] OR "atypism"[All Fields] OR "atypisms"[All Fields]) AND ("diffusable"[All Fields] OR "diffusant"[All Fields] OR "diffusants"[All Fields] OR "Diffuse"[All Fields] OR "diffusely"[All Fields] OR "diffuses"[All Fields] OR "diffusibility"[All Fields] OR "diffusible"[All Fields] OR "diffusion"[MeSH Terms] OR "diffusion"[All Fields] OR "diffused"[All Fields] OR "diffusing"[All Fields] OR "diffusions"[All Fields] OR "diffusive"[All Fields] OR "diffusively"[All Fields] OR "diffusivities"[All Fields] OR "diffusivity"[All Fields])) AND "small lymphoid"[Title/Abstract]) OR "diffuse small cleaved cell lymphoma"[Title/Abstract] OR "diffuse small cleaved cell lymphoma"[Title/Abstract] OR (("Lymphoma"[MeSH Terms] OR "Lymphoma"[All Fields] OR "Lymphomas"[All Fields] OR "lymphoma s"[All Fields]) AND "small cleaved cell diffuse"[Title/Abstract]) OR (("Lymphoma"[MeSH Terms] OR "Lymphoma"[All Fields] OR "Lymphomas"[All Fields] OR "lymphoma s"[All Fields]) AND "Nonhodgkins"[Title/Abstract]) OR "nonhodgkins lymphoma"[Title/Abstract] OR "lymphoma non hodgkins"[Title/Abstract] OR "lymphoma non hodgkins"[Title/Abstract] OR "non hodgkins lymphoma"[Title/Abstract] OR	

For peer review only

	"non hodgkin lymphoma"[Title/Abstract] OR "non hodgkin lymphoma"[Title/Abstract] OR "lymphoma nonhodgkin s"[Title/Abstract] OR "lymphoma nonhodgkin"[Title/Abstract] OR "small cleaved cell lymphoma diffuse"[Title/Abstract] OR "small cleaved cell lymphoma diffuse"[Title/Abstract] OR "lymphoma non hodgkin s"[Title/Abstract] OR "lymphoma non hodgkin s"[Title/Abstract] OR "lymphoma diffuse"[Title/Abstract] OR "diffuse lymphoma"[Title/Abstract] OR "diffuse lymphomas"[Title/Abstract] OR "sarcoma lymphatic"[Title/Abstract] OR "Lymphosarcoma"[Title/Abstract] OR "Lymphosarcomas"[Title/Abstract] OR "lymphatic sarcoma"[Title/Abstract] OR "lymphatic sarcomas"[Title/Abstract] OR "lymphoma low grade"[Title/Abstract] OR "low grade lymphoma"[Title/Abstract] OR "low grade lymphomas"[Title/Abstract] OR "lymphoma low grade"[Title/Abstract] OR "lymphoma mixed cell"[Title/Abstract] OR "lymphoma mixed cell"[Title/Abstract] OR "mixed cell lymphoma"[Title/Abstract] OR "mixed cell lymphoma"[Title/Abstract] OR "mixed cell lymphomas"[Title/Abstract] OR "lymphoma mixed"[Title/Abstract] OR "mixed lymphoma"[Title/Abstract] OR "mixed lymphomas"[Title/Abstract] OR "lymphoma mixed lymphocytic histiocytic"[Title/Abstract] OR ("Lymphocytic-Histiocytic"[All Fields] AND "lymphoma mixed"[Title/Abstract]) OR ("Lymphocytic-Histiocytic"[All Fields] AND "lymphomas mixed"[Title/Abstract]) OR "lymphoma mixed lymphocytic histiocytic"[Title/Abstract] OR "mixed lymphocytic histiocytic lymphoma"[Title/Abstract] OR "mixed lymphocytic histiocytic lymphomas"[Title/Abstract] OR (("lymphoma, non hodgkin"[MeSH Terms] OR ("Lymphoma"[All Fields] AND "Non-Hodgkin"[All Fields]) OR "non-hodgkin lymphoma"[All Fields] OR ("Lymphoma"[All Fields] AND "Non"[All Fields] AND "Hodgkin"[All Fields]) OR "lymphoma non hodgkin"[All Fields]) AND "Familial"[Title/Abstract]) OR "lymphoma small noncleaved cell"[Title/Abstract] OR "lymphoma small noncleaved cell"[Title/Abstract] OR "small noncleaved cell lymphoma"[Title/Abstract] OR ("Noncleaved-Cell"[All Fields] AND "lymphoma small"[Title/Abstract]) OR "small noncleaved cell lymphoma"[Title/Abstract] OR "small noncleaved cell lymphomas"[Title/Abstract] OR "diffuse undifferentiated lymphoma"[Title/Abstract] OR "diffuse undifferentiated lymphomas"[Title/Abstract] OR "lymphoma diffuse undifferentiated"[Title/Abstract] OR "undifferentiated lymphoma diffuse"[Title/Abstract] OR "lymphoma small non cleaved cell"[Title/Abstract] OR "lymphoma small non cleaved cell"[Title/Abstract] OR ("Non-Cleaved-Cell"[All Fields] AND "lymphoma small"[Title/Abstract]) OR "small non cleaved cell lymphomas"[Title/Abstract] OR "small non cleaved cell lymphoma"[Title/Abstract] OR "small non cleaved cell lymphoma"[Title/Abstract] OR (("Lymphoma"[MeSH Terms] OR "Lymphoma"[All Fields] OR "Lymphomas"[All Fields] OR "lymphoma s"[All Fields]) AND "undifferentiated diffuse"[Title/Abstract]) OR "lymphoma	

For peer review only

	intermediate grade"[Title/Abstract] OR "intermediate grade lymphoma"[Title/Abstract] OR "intermediate grade lymphomas"[Title/Abstract] OR "lymphoma intermediate grade"[Title/Abstract] OR "lymphoma undifferentiated"[Title/Abstract] OR "lymphoma pleomorphic"[Title/Abstract] OR "pleomorphic lymphoma"[Title/Abstract] OR "diffuse mixed cell lymphomas"[Title/Abstract] OR "diffuse mixed cell lymphoma"[Title/Abstract] OR "diffuse mixed cell lymphoma"[Title/Abstract] OR "non hodgkin s lymphoma"[Title/Abstract] OR "non hodgkin s lymphoma"[Title/Abstract] OR "nonhodgkin s lymphoma"[Title/Abstract] OR ("diffuse mixed small"[Title/Abstract] AND "large cell lymphoma"[Title/Abstract]) OR (("Mixed"[All Fields] OR "mixes"[All Fields] OR "mixing"[All Fields] OR "mixings"[All Fields]) AND "cell lymphoma diffuse"[Title/Abstract]) OR ("Mixed-Cell"[All Fields] AND "lymphoma diffuse"[Title/Abstract]) OR (("Lymphoma"[MeSH Terms] OR "Lymphoma"[All Fields] OR "Lymphomas"[All Fields] OR "lymphoma s"[All Fields]) AND "mixed cell diffuse"[Title/Abstract]) OR ("lymphoma small"[Title/Abstract] AND ((("Large"[All Fields] OR "largely"[All Fields] OR "larges"[All Fields]) AND "Cleaved-Cell"[All Fields]) AND "Diffuse"[Title/Abstract])) OR (("lymphoma, non hodgkin"[MeSH Terms] OR ("Lymphoma"[All Fields] AND "Non-Hodgkin"[All Fields]) OR "non-hodgkin lymphoma"[All Fields] OR ("Lymphoma"[All Fields] AND "Diffuse"[All Fields]) OR "lymphoma diffuse"[All Fields]) AND "mixed lymphocytic histiocytic"[Title/Abstract]) OR ("lymphoma mixed small"[Title/Abstract] AND "large cell diffuse"[Title/Abstract]) OR ("mixed small"[Title/Abstract] AND "large cell lymphoma diffuse"[Title/Abstract]) OR (("Lymphoma"[MeSH Terms] OR "Lymphoma"[All Fields] OR "Lymphomas"[All Fields] OR "lymphoma s"[All Fields]) AND "mixed cell diffuse"[Title/Abstract]) OR "lymphoma high grade"[Title/Abstract] OR "high grade lymphomas"[Title/Abstract] OR "high grade lymphoma"[Title/Abstract] OR "lymphoma high grade"[Title/Abstract] OR "Reticulosarcomas"[Title/Abstract] OR "Reticulosarcoma"[Title/Abstract] OR "reticulum cell sarcomas"[Title/Abstract] OR "reticulum cell sarcoma"[Title/Abstract] OR "sarcoma reticulum cell"[Title/Abstract] OR "sarcoma reticulum cell"[Title/Abstract] OR "reticulum cell sarcomas"[Title/Abstract] OR "reticulum cell sarcoma"[Title/Abstract] OR "undifferentiated lymphomas"[Title/Abstract] OR "undifferentiated lymphoma"[Title/Abstract] OR "pleomorphic lymphomas"[Title/Abstract]	
#4	"prevalence"[Text Word] OR "incidence"[Text Word] OR "epidemiol*"[Text Word] OR "survey"[Text Word] OR "rapid assessment"[Text Word] OR "situation assessment"[Text Word] OR "situational assessment"[Text Word] OR "rar"[Text Word] OR "cohort"[Text Word] OR "surveillance"[Text Word] OR "seroprevalence"[Text Word] OR "seroincidence"[Text Word] OR "screening"[Text Word] OR "mortality"[Title/Abstract] OR "mortalities"[Title/Abstract] OR "mortal"[Title/Abstract] OR "dead"[Title/Abstract] OR "death"[Title/Abstract] OR "deaths"[Title/Abstract]	

For peer review only

	OR "decease*"[Title/Abstract] OR "demise*"[Title/Abstract] OR "die"[Title/Abstract] OR "died"[Title/Abstract] OR "dies"[Title/Abstract] OR "dying"[Title/Abstract] OR "fatal"[Title/Abstract] OR "fatality"[Title/Abstract] OR "fatalities"[Title/Abstract] OR "nonsurvival*"[Title/Abstract] OR "nonsurvival*"[Title/Abstract] OR "survival*"[Title/Abstract] OR "lethality"[Title/Abstract] OR "epidemiologic methods"[MeSH Major Topic] OR "epidemiologic studies"[MeSH Major Topic] OR "sentinel surveillance"[MeSH Major Topic] OR "seroepidemiologic studies"[MeSH Major Topic] OR "cohort studies"[MeSH Major Topic] OR "longitudinal studies"[MeSH Major Topic] OR "follow up studies"[MeSH Major Topic] OR "prospective studies"[MeSH Major Topic] OR "diagnostic services"[MeSH Major Topic] OR "mass screening"[MeSH Major Topic] OR "mortality"[MeSH Major Topic] OR "hospital mortality"[MeSH Major Topic] OR "death"[MeSH Major Topic] OR "cause of death"[MeSH Major Topic] OR "death certificates"[MeSH Major Topic] OR "fatal outcome"[MeSH Major Topic] OR "autopsy"[MeSH Major Topic] OR "survival rate"[MeSH Major Topic]	
#5	#1 or #2	
#6	#3 and #4 and #5	

Embase

Set	Search	Results
#1	'human immunodeficiency virus infection'/exp OR 'human immunodeficiency virus'/exp OR 'hiv survivor'/exp OR hiv:ti,ab,kw OR 'hiv-1':ti,ab,kw OR 'hiv-2':ti,ab,kw OR 'human immunodeficiency virus':ti,ab,kw OR 'human immunodeficiency':ti,ab,kw OR 'human immunedeficiency virus':ti,ab,kw OR 'human immune deficiency virus':ti,ab,kw OR 'human immune-deficiency virus':ti,ab,kw OR 'acquired immune-deficiency syndrome':ti,ab,kw OR 'acquired immunedeficiency syndrome':ti,ab,kw OR 'acquired immunodeficiency syndrome':ti,ab,kw OR 'acquired immuno-deficiency syndrome':ti,ab,kw	
#2	prevalence:ti,ab,de OR incidence:ti,ab,de OR epidemiol*:ti,ab,de OR survey:ti,ab,de OR 'rapid assessment':ti,ab,de OR 'situation assessment':ti,ab,de OR 'situational assessment':ti,ab,de OR rar:ti,ab,de OR cohort:ti,ab,de OR surveillance:ti,ab,de OR seroprevalence:ti,ab,de OR seroincidence:ti,ab,de OR seroepidemiol*:ti,ab,de OR screening:ti,ab,de OR 'mass screening':ti,ab,de OR mortality:ti,ab OR mortalities:ti,ab OR mortal:ti,ab OR dead:ti,ab OR death:ti,ab OR deaths:ti,ab OR decease*:ti,ab OR demise*:ti,ab OR die:ti,ab OR died:ti,ab OR dies:ti,ab OR dying:ti,ab OR fatal:ti,ab OR fatality:ti,ab OR fatalities:ti,ab OR 'non survival*':ti,ab OR nonsurvival*:ti,ab OR survival*:ti,ab OR lethality:ti,ab OR epidemiology:de OR 'cancer epidemiology'/exp OR 'cancer statistics'/exp OR 'disease surveillance'/exp OR 'health survey'/exp OR 'incidence'/exp OR 'infection rate'/exp OR 'prevelance' OR 'sentinel surveillance'/exp OR	

	'seroepidemiology'/exp OR 'cohort analysis'/exp OR 'longitudinal study'/exp OR 'prospective study'/exp OR 'preventive health service'/exp OR 'mass screening'/exp OR 'cancer mortality'/exp OR 'all cause mortality'/exp OR 'mortality':de OR 'hospital mortality'/exp OR 'mortality rate'/exp OR 'death'/exp OR 'cause of death'/exp OR 'dying'/exp OR 'fatality'/exp OR 'lethality'/exp OR 'death certificate'/exp OR 'autopsy'/exp OR 'cancer survival'/exp OR 'survival rate'/exp	
#3	'non-hodgkin lymphoma'/exp	
#4	'lymphoma, non hodgkin':ab,ti	
#5	'lymphoma, small cleaved-cell, diffuse':ab,ti	
#6	'lymphoma, atypical diffuse small lymphoid':ab,ti	
#7	'diffuse small cleaved-cell lymphoma':ab,ti	
#8	'diffuse small cleaved cell lymphoma':ab,ti	
#9	'lymphoma, small cleaved cell, diffuse':ab,ti	
#10	'lymphoma, nonhodgkins':ab,ti	
#11	'nonhodgkins lymphoma':ab,ti	
#12	'lymphoma, non-hodgkins':ab,ti	
#13	'lymphoma, non hodgkins':ab,ti	
#14	'lymphoma, non hodgkins':ab,ti	
#15	'non-hodgkins lymphoma':ab,ti	
#16	'non-hodgkin lymphoma':ab,ti	
#17	'lymphoma, nonhodgkin':ab,ti	
#18	'small cleaved-cell lymphoma, diffuse':ab,ti	
#19	'lymphoma, diffuse':ab,ti	
#20	'ldiffuse lymphomae':ab,ti	
#21	'diffuse lymphomae':ab,ti	
#22	'diffuse lymphomas':ab,ti	
#23	'diffuse lymphoma':ab,ti	
#24	'sarcoma, lymphatic':ab,ti	
#25	'lymphosarcoma':ab,ti	
#26	'lymphosarcomas':ab,ti	
#27	'llymphatic sarcomas':ab,ti	
#28	'lymphatic sarcomas':ab,ti	
#29	'lymphatic sarcomas':ab,ti	
#30	'lymphoma, low-grade':ab,ti	
#31	'low-grade lymphoma':ab,ti	
#32	'low-grade lymphomas':ab,ti	
#33	'lymphoma, low grade':ab,ti	
#34	'lymphoma, mixed-cell':ab,ti	
#35	'lymphoma, mixed cell':ab,ti	
#36	'mixed-cell lymphoma':ab,ti	
#37	'mixed cell lymphoma':ab,ti	
#38	'mixed-cell lymphomas':ab,ti	

For peer review only

#39	'lymphoma, mixed':ab,ti	
#40	'mixed lymphoma':ab,ti	
#41	'lymphoma, mixed lymphocytic-histiocytic':ab,ti	
#42	'lymphocytic-histiocytic lymphoma, mixed':ab,ti	
#43	'mixed lymphocytic-histiocytic lymphoma':ab,ti	
#44	'mixed lymphocytic-histiocytic lymphomas':ab,ti	
#45	'lymphoma, non-hodgkin, familial':ab,ti	
#46	'lymphoma, small noncleaved-cell':ab,ti	
#47	'lymphoma, small noncleaved cell':ab,ti	
#48	'small noncleaved-cell lymphoma':ab,ti	
#49	'noncleaved-cell lymphoma, small':ab,ti	
#50	'small noncleaved cell lymphoma':ab,ti	
#51	'small noncleaved-cell lymphomas':ab,ti	
#52	'diffuse undifferentiated lymphoma':ab,ti	
#53	'diffuse undifferentiated lymphomas':ab,ti	
#54	#3 OR #4 OR #5 OR #6 OR #7 OR #8 OR #9 OR #10 OR #11 OR #12 OR #13 OR #14 OR #15 OR #16 OR #17 OR #18 OR #19 OR #20 OR #21 OR #22 OR #23 OR #24 OR #25 OR #26 OR #27 OR #28 OR #29 OR #30 OR #31 OR #32 OR #33 OR #34 OR #35 OR #36 OR #37 OR #38 OR #39 OR #40 OR #41 OR #42 OR #43 OR #44 OR #45 OR #46 OR #47 OR #48 OR #49 OR #50 OR #51 OR #52 OR #53	
#55	#1 AND #2 AND #54	

Cochrane Library

Set	Search	Results
#1	MeSH descriptor: [HIV] explode all trees	
#2	(hiv):ti,ab,kw (Word variations have been searched)	
#3	#1 OR #2	
#4	("non-Hodgkin lymphoma"):ti,ab,kw (Word variations have been searched)	
#5	MeSH descriptor: [Lymphoma, Non-Hodgkin] explode all trees	
#6	#4 OR #5	
#7	#6 OR #3	

For peer review only

Web of Science

Set	Search	Results
#1	"TS=(prevalence OR incidence OR epidemiol* OR survey OR ""rapid assessment"" OR ""situation assessment"" OR ""situational assessment"" OR rar OR cohort OR surveillance OR seroprevalence OR seroincidence OR seroepidemiol* OR screening OR mortality OR mortalities OR mortal OR dead OR death OR deaths OR decease* OR demise* OR die OR died OR dies OR dying OR fatal OR fatality OR fatalities OR non-survival* OR	

	Nonsurvival* OR survival* OR lethality OR ""longitudinal studies"" OR ""follow-up studies"" OR ""prospective studies"" OR ""Mass Screening"" OR ""Autopsy"")	
#2	"TS=(“hiv” OR “hiv-1” OR “hiv-2” OR “hiv1” OR “hiv2” OR hiv infect* OR “human immunodeficiency virus” OR “human immunedeficiency virus” OR “human immuno-deficiency virus” OR “human immune-deficiency virus” OR ((human immun*) AND (“deficiency virus”)) OR “acquired immunodeficiency syndrome” OR “acquired immunedeficiency syndrome” OR “acquired immuno-deficiency syndrome” OR “acquired immune-deficiency syndrome” OR ((acquired immun*) AND (“deficiency syndrome”)))	
#3	"TS=(“Non-Hodgkin lymphoma” OR “Lymphoma, Non Hodgkin” OR “Lymphoma, Small Cleaved-Cell, Diffuse” OR “Lymphoma, Atypical Diffuse Small Lymphoid” OR “Diffuse Small Cleaved-Cell Lymphoma” OR “Diffuse Small Cleaved Cell Lymphoma” OR “Lymphoma, Small Cleaved Cell, Diffuse” OR “Lymphoma, Nonhodgkins” OR “Nonhodgkins Lymphoma” OR “Lymphoma, Non-Hodgkins” OR “Lymphoma, Non Hodgkins” OR “Non-Hodgkins Lymphoma” OR “Non-Hodgkin Lymphoma” OR “Non Hodgkin Lymphoma” OR “Lymphoma, Nonhodgkin's” OR “Lymphoma, Nonhodgkin” OR “Small Cleaved-Cell Lymphoma, Diffuse” OR “Small Cleaved Cell Lymphoma, Diffuse” OR “Lymphoma, Non-Hodgkin's” OR “Lymphoma, Non Hodgkin's” OR “Lymphoma, Diffuse” OR “Diffuse Lymphoma” OR “Diffuse Lymphomas” OR “Sarcoma, Lymphatic” OR “Lymphosarcoma” OR “Lymphosarcomas” OR “Lymphatic Sarcoma” OR “Lymphatic Sarcomas” OR “Lymphoma, Low-Grade” OR “Low-Grade Lymphoma” OR “Low-Grade Lymphomas” OR “Lymphoma, Low Grade” OR “Lymphoma, Mixed-Cell” OR “Lymphoma, Mixed Cell” OR “Mixed-Cell Lymphoma” OR “Mixed Cell Lymphoma” OR “Mixed-Cell Lymphomas” OR “Lymphoma, Mixed” OR “Mixed Lymphoma” OR “Mixed Lymphomas” OR “Lymphoma, Mixed Lymphocytic-Histiocytic”OR “Lymphocytic-Histiocytic Lymphoma, Mixed” OR “Lymphocytic-Histiocytic Lymphomas, Mixed” OR “Lymphoma, Mixed Lymphocytic Histiocytic” OR “Mixed Lymphocytic-Histiocytic Lymphoma” OR “Mixed Lymphocytic-Histiocytic Lymphomas” OR “Lymphoma, Non-Hodgkin, Familial” OR “Lymphoma, Small Noncleaved-Cell” OR “Lymphoma, Small Noncleaved Cell” OR “Small Noncleaved-Cell Lymphoma” OR “Noncleaved-Cell Lymphoma, Small” OR “Small Noncleaved Cell Lymphoma” OR “Small Noncleaved-Cell Lymphomas” OR “Diffuse Undifferentiated Lymphoma” OR “Diffuse Undifferentiated Lymphomas” OR “Lymphoma, Diffuse Undifferentiated” OR “Undifferentiated Lymphoma, Diffuse” OR “Lymphoma, Small Non-Cleaved-Cell” OR “Lymphoma, Small Non Cleaved Cell” OR “Non-Cleaved-Cell Lymphoma, Small” OR “Small Non-Cleaved-Cell Lymphomas” OR “Small Non-Cleaved-Cell Lymphoma” OR “Small Non Cleaved Cell Lymphoma” OR “Lymphoma, Undifferentiated,	

For peer review only

	Diffuse” OR “Lymphoma, Intermediate-Grade” OR “Intermediate-Grade Lymphoma” OR “Intermediate-Grade Lymphomas” OR “Lymphoma, Intermediate Grade” OR “Lymphoma, Undifferentiated” OR “Lymphoma, Pleomorphic” OR “Pleomorphic Lymphoma” OR “Pleomorphic Lymphomas” OR “Undifferentiated Lymphoma” OR “Undifferentiated Lymphomas” OR “Reticulum-Cell Sarcoma” OR “Reticulum-Cell Sarcomas” OR “Sarcoma, Reticulum-Cell” OR “Sarcoma, Reticulum Cell” OR “Reticulum Cell Sarcoma” OR “Reticulum Cell Sarcomas” OR “Reticulosarcoma” OR “Reticulosarcomas” OR “Lymphoma, High-Grade” OR “High-Grade Lymphoma” OR “High-Grade Lymphomas” OR “Lymphoma, High Grade” OR “Lymphoma, Mixed-Cell, Diffuse” OR “Mixed Small and Large Cell Lymphoma, Diffuse” OR “Lymphoma, Mixed Small and Large Cell, Diffuse” OR “Lymphoma, Diffuse, Mixed Lymphocytic-Histiocytic” OR “Lymphoma, Small and Large Cleaved-Cell, Diffuse” OR “Lymphoma, Mixed Cell, Diffuse” OR “Mixed-Cell Lymphoma, Diffuse” OR “Mixed Cell Lymphoma, Diffuse” OR “Diffuse Mixed Small and Large Cell Lymphoma” OR “Diffuse Mixed-Cell Lymphoma” OR “Diffuse Mixed Cell Lymphoma” OR “Diffuse Mixed-Cell Lymphomas” OR “Lymphoma, Diffuse Mixed-Cel” OR “Non-Hodgkin's Lymphoma” OR “Non Hodgkin's Lymphoma” OR “Nonhodgkin's Lymphoma”)	
#4	#1 AND #2 AND #3	

Appendix 2. Countries and regions by UNAIDS

UNAIDS Regions	Country	ISO Code	Unaids Regions	Country	ISO Code
**Global**	~	03M49WLD	**Latin America**	Argentina	ARG
**Asia and the Pacific**	Afghanistan	AFG		Bolivia	BOL
	Australia	AUS		Brazil	BRA
	Bangladesh	BGD		Chile	CHL
	Bhutan	BTN		Colombia	COL
	Brunei	BRN		Costa Rica	CRI
	Cambodia	KHM		Ecuador	ECU
	China	CHN		El Salvador	SLV
	South Korea	PRK		Guatemala	GTM
	Micronesia	FSM		Honduras	HND
	Fiji	FJI		Mexico	MEX
	India	IND		Nicaragua	NIC
	Indonesia	IDN		Panama	PAN
	Japan	JPN		Paraguay	PRY
	Kiribati	KIR		Peru	PER
	Laos	LAO		Uruguay	URY
	Malaysia	MYS		Venezuela	VEN
	Maldives	MDV	**Middle East and North Africa**	Algeria	DZA
	Marshall Islands	MHL		Bahrain	BHR
	Mongolia	MNG		Djibouti	DJI
	Myanmar	MMR		Egypt	EGY
	Nauru	NRU		Iraq	IRQ

For peer review only

	Nepal	NPL		Iran	IRN
	New Zealand	NZL		Jordan	JOR
	Pakistan	PAK		Kuwait	KWT
	Palau	PLW		Lebanon	LBN
	Papua New Guinea	PNG		Libya	LBY
	Philippines	PHL		Morocco	MAR
	North Korea	KOR		Oman	OMN
	Samoa	WSM		Qatar	QAT
	Singapore	SGP		Saudi Arabia	SAU
	Solomon Islands	SLB		Somalia	SOM
	Sri Lanka	LKA		Sudan	SDN
	Thailand	THA		Syria	SYR
	Timor-Leste	TLS		Tunisia	TUN
	Tonga	TON		United Arab Emirates	ARE
	Tuvalu	TUV		Yemen	YEM
	Vanuatu	VUT	**Western and central Africa**	Benin	BEN
	Vietnam	VNM		Burkina Faso	BFA
**Caribbean**	Antigua	ATG		Burundi	BDI
	Bahamas	BMH		Ivory Coast	CIV
	Barbados	BRB		Cameroon	CMR
	Belize	BLZ		Cape Verde	CPV
	Cuba	CUB		Central African Republic	CAF
	Dominica	DMA		Chad	TCD
	Dominican Republic	DOM		Republic of Congo	COG
	Grenada	GRD		Democratic Republic of	COD

For peer review only

				the Congo	
	Guyana	GUY		Equatorial Guinea	GNQ
	Haiti	HTI		Gabon	GAB
	Jamaica	JAM		Gambia	GMB
	Saint Kitts	KNA		Ghana	GHA
	Saint Lucia	LCA		Guinea	GIN
	Saint Vincent	VCT		Guinea-Bissau	GNB
	Suriname	SUR		Liberia	LBR
	Trinidad	TTO		Mali	MLI
**Eastern and southern Africa**	Angola	AGO		Mauritania	MRT
	Botswana	BWA		Niger	NER
	Comoros	COM		Nigeria	NGA
	Eritrea	ERI		Sao Tome and Principe	STP
	Swaziland	SWZ		Senegal	SEN
	Ethiopia	ETH		Sierra Leone	SLE
	Kenya	KEN		Togo	TGO
	Lesotho	LSO	**Western and central Europe and North America**	Andorra	AND
	Madagascar	MDG		Austria	AUT
	Malawi	MWI		Belgium	BEL
	Mauritius	MUS		Bulgaria	BGR
	Mozambique	MOZ		Canada	CAN
	Namibia	NAM		Croatia	HRV
	Rwanda	RWA		Cyprus	CYP
	Seychelles	SYC		Czech Republic	CZE
	South Africa	ZAF		Denmark	DNK

For peer review only

	South Sudan	SSD		Estonia	EST
	Uganda	UGA		Finland	FIN
	Tanzania	TZA		France	FRA
	Zambia	ZMB		Germany	DEU
	Zimbabwe	ZWE		Greece	GRC
**Eastern Europe and central Asia**	Albania	ALB		Hungary	HUN
	Armenia	ARM		Iceland	ISL
	Azerbaijan	AZE		Ireland	IRL
	Belarus	BLR		Israel	ISR
	Bosnia and Herzegovina	BIH		Italy	ITA
	Georgia	GEO		Latvia	LVA
	Kazakhstan	KAZ		Lithuania	LTU
	Kyrgyzstan	KGZ		Luxembourg	LUX
	Montenegro	MNE		Netherlands	NLD
	Moldova	MDA		Norway	NOR
	Russia	RUS		Poland	POL
	Tajikistan	TJK		Portugal	PRT
	Micronesia (Federated States of)	MKD		Romania	ROU
	Turkmenistan	TKM		Serbia	SRB
	Ukraine	UKR		Slovakia	SVK
	Uzbekistan	UZB		Slovenia	SVN
				Spain	ESP
				Sweden	SWE
				Switzerland	CHE

For peer review only

For peer review only - http://bmjopen.bmj.com/site/about/guidelines.xhtml

				Turkey	TUR
				UK	GBR
				USA	USA

For peer review only

PRISMA-P (preferred reporting items for systematic review and meta-analysis protocols) 2015 checklist: recommended items to address in a systematic review protocol

Section and topic	Item No	Checklist item	Page number
**Administrative information**			
Title:			
Identification	1a	Identify the report as a protocol of a systematic review	1
Update	1b	If the protocol is for an update of a previous systematic review, identify as such	NA
Registration	2	If registered, provide the name of the registry (such as PROSPERO) and registration number	3 & 6
Authors:			
Contact	3a	Provide name, institutional affiliation, e-mail address of all protocol authors; provide physical mailing address of corresponding author	1
Contributions	3b	Describe contributions of protocol authors and identify the guarantor of the review	12
Amendments	4	If the protocol represents an amendment of a previously completed or published protocol, identify as such and list changes; otherwise, state plan for documenting important protocol amendments	NA
Support:			
Sources	5a	Indicate sources of financial or other support for the review	12
Sponsor	5b	Provide name for the review funder and/or sponsor	12
Role of sponsor or funder	5c	Describe roles of funder(s), sponsor(s), and/or institution(s), if any, in developing the protocol	NA
**Introduction**			
Rationale	6	Describe the rationale for the review in the context of what is already known	4 & 5
Objectives	7	Provide an explicit statement of the question(s) the review will address with reference to participants, interventions, comparators, and outcomes (PICO)	5
**Methods**			
Eligibility criteria	8	Specify the study characteristics (such as PICO, study design, setting, time frame) and report characteristics (such as years considered, language, publication status) to be used as criteria for eligibility for the review	6
Information sources	9	Describe all intended information sources (such as electronic databases, contact with study authors, trial registers or other grey literature sources) with planned dates of coverage	6 & 9
Search strategy	10	Present draft of search strategy to be used for at least one electronic database, including planned limits, such that it could be repeated	6

Study records:			
Data management	11a	Describe the mechanism(s) that will be used to manage records and data throughout the review	7
Selection process	11b	State the process that will be used for selecting studies (such as two independent reviewers) through each phase of the review (that is, screening, eligibility and inclusion in meta-analysis)	7 & 8
Data collection process	11c	Describe planned method of extracting data from reports (such as piloting forms, done independently, in duplicate), any processes for obtaining and confirming data from investigators	8
Data items	12	List and define all variables for which data will be sought (such as PICO items, funding sources), any pre-planned data assumptions and simplifications	8 & 12
Outcomes and prioritization	13	List and define all outcomes for which data will be sought, including prioritization of main and additional outcomes, with rationale	6
Risk of bias in individual studies	14	Describe anticipated methods for assessing risk of bias of individual studies, including whether this will be done at the outcome or study level, or both; state how this information will be used in data synthesis	9
Data synthesis	15a	Describe criteria under which study data will be quantitatively synthesised	6
	15b	If data are appropriate for quantitative synthesis, describe planned summary measures, methods of handling data and methods of combining data from studies, including any planned exploration of consistency (such as I^2, Kendall's τ)	9
	15c	Describe any proposed additional analyses (such as sensitivity or subgroup analyses, meta-regression)	9
	15d	If quantitative synthesis is not appropriate, describe the type of summary planned	NA
Meta-bias(es)	16	Specify any planned assessment of meta-bias(es) (such as publication bias across studies, selective reporting within studies)	9
Confidence in cumulative evidence	17	Describe how the strength of the body of evidence will be assessed (such as GRADE)	8

From: Shamseer L, Moher D, Clarke M, Ghersi D, Liberati A, Petticrew M, Shekelle P, Stewart L, PRISMA-P Group. Preferred reporting items for systematic review and meta-analysis protocols (PRISMA-P) 2015: elaboration and explanation. BMJ. 2015 Jan 2;349(jan02 1):g7647.

BMJ Open

Global and regional burden estimation of HIV-associated non-Hodgkin lymphoma: a meta-analysis and modeling analysis protocol

Journal:	*BMJ Open*
Manuscript ID	bmjopen-2023-075933.R2
Article Type:	Protocol
Date Submitted by the Author:	22-Dec-2023
Complete List of Authors:	Chen, Yan; Southwest Medical University, School of Public Health; University of Electronic Science and Technology of China, Department of Health Management Center & Institute of Health Management<br>Sun, Zhaochen; Southwest Medical University, School of Public Health; University of Electronic Science and Technology of China, Department of Health Management Center & Institute of Health Management<br>Sun, Ping; University of Electronic Science and Technology of China, Department of Health Management Center & Institute of Health Management<br>Liu, Yuping; University of Electronic Science and Technology of China, Department of Health Management Center & Institute of Health Management<br>Wan, Zhengwei; University of Electronic Science and Technology of China, Department of Health Management Center & Institute of Health Management<br>Ye, Yunli; Southwest Medical University, School of Public Health
**Primary Subject Heading**:	Global health
Secondary Subject Heading:	Oncology, HIV/AIDS, Public health
Keywords:	HIV & AIDS < INFECTIOUS DISEASES, Lymphoma < ONCOLOGY, Meta-Analysis, PUBLIC HEALTH

SCHOLARONE™
Manuscripts

Global and regional burden estimation of HIV-associated non-Hodgkin lymphoma: a meta-analysis and modeling analysis protocol

Yan Chen[1,2*], Zhaochen Sun[1,2*], Ping Sun[2], Yuping Liu[2], Zhengwei Wan[2#], Yunli Ye[1#]

[1]School of Public Health, Southwest Medical University, Luzhou, China.

[2]Department of Health Management Center & Institute of Health Management, Sichuan Provincial People's Hospital, University of Electronic Science and Technology of China, Chengdu, China.

Email address:

Yan Chen: chenyan122071@163.com

Zhaochen Sun: 13585928551@163.com

Ping Sun: sunping612@foxmail.com

Yuping Liu: 18981838972@163.com

* These authors contributed equally and share first authorship.

[#] Corresponding authors

Prof. Yunli Ye

Address: No.1 First Section, Xianglin Rd, Longmatan Dist, Luzhou, China;

E-mail: wushuangyewu@163.com;

Tel: +86 18982785179.

Dr. Zhengwei Wan

Address: No. 32 West Second Section, First Ring Rd., Qingyang Dist., Chengdu, China;

E-mail: 18715799366@163.com;

Tel: +86 18715799366.

For peer review only - http://bmjopen.bmj.com/site/about/guidelines.xhtml

ABSTRACT

Introduction Human immunodeficiency virus (HIV) infection is one of the complex etiologies of non-Hodgkin lymphoma (NHL). However, the contribution of HIV to burden of NHL across time and region has not yet been comprehensively reported and quantified. Thus, this study aims to evaluate the relative risk of NHL in individuals with HIV-infection compared to those without by performing a comprehensive meta-analysis. Additionally, we intend to further estimate quantitatively the degree of HIV contributing to burden of NHL using population attributable fraction (PAF) modeling analysis.

Methods and analysis This study will screen a mass of records searched from four electronic databases (PubMed, Embase, Cochrane Library, and Web of Science). The main outcomes are specific effect values and corresponding 95% confidence intervals (CIs) for NHL among population with HIV compared to those without to quantify the association between HIV-infection and NHL. After quality assessment and data extraction, we will undertake a meta-analysis to calculate the pooled risk ratio (RR). Furthermore, PAF calculation based on pooled RR combines with number of age-specific disability-adjusted life year (DALY) and HIV prevalence data (aged ⩾ 15 years old) from 1990 to 2019, at global, regional, and country levels. We will calculate the PAF, HIV-associated DALY number and age-standardised rate (ASR) to quantify the burden of HIV-associated NHL.

Ethics and dissemination This study is based on published articles thus the ethic approval is not essential. In addition, we intend to publish the results on peer-reviewed journal for more discussion. We believe that research on estimating global burden of NHL can provide valuable insights for developing targeted prevention and control strategies, thereby achieving significant benefits.

PROSPERO registration number: CRD 42023404150.

For peer review only - http://bmjopen.bmj.com/site/about/guidelines.xhtml

Keywords: HIV, non-Hodgkin lymphoma, meta-analysis, population attributable fraction, global burden

Strengths and limitations of this study

1. The study will include meta-analysis and population attributable fraction (PAF) estimation modeling analysis, which provides aid in the formulation of precise prevention and control policies.

2. The quality of eligible studies will be assessed using an 11-iterm checklist recommended by the Agency for Healthcare Research and Quality (AHRQ).

3. The dissemination of the results may be limited because of the language restriction.

4. The pooled RR value will be applied to PAF modeling across global and regional areas, which may lead to bias.

For peer review only - http://bmjopen.bmj.com/site/about/guidelines.xhtml

Introduction

Non-Hodgkin lymphoma (NHL) is a common lymphoid tissues neoplasm that originates from B cell precursors, mature B cells, T cell precursors, and mature T cells.[1] According to the World Health Organization (WHO) classification, the most common NHL in western countries are diffuse large B cell lymphoma (DLBCL) (accounting for around 31% of adult cases), follicular lymphoma (FL) (22%) and Burkitt lymphoma (BL) (2%).[2, 3] According to GLOBOCAN database of WHO's International Agency for Research on Cancer (IARC), NHL accounted for 2.8% of total cancers with 544,352 new cases, and 2.6% of total cancer-related deaths with 259,793 new deaths, ranking NHL within the top 10 cancers for both males (ranked 8th) and females (ranked 10th) in 2020.[4]

The etiology of NHL exhibits significant complexity. Published articles have identified various potential causes of NHL, including immunosuppression, viral infection (e.g., human immunodeficiency virus, HIV), family factor, occupational and environmental exposures, organ transplantation, and autoimmune diseases.[5, 6, 7] NHL is regarded as a defining event of acquired immune deficiency syndrome (AIDS),[6] thus establishing a strong connection between HIV and NHL. Based on a population-based review encompassing the United States, Australia, and Italy, the risk of NHL among individuals with AIDS compared to general population, ranged from 15-fold for low-grade NHL to 400-fold for high-grade NHL.[8] In addition, certain subtypes of NHL have been found to be associated with HIV infection. Another multi-cohort study conducted in Europe revealed a higher incidence of NHL among individuals with HIV, regardless of whether they received combination antiretroviral therapy (cART) treatment.[9] Although the introduction of cART in the late 1990s has significantly reduced the incidence of NHL,[10, 11] such as the United States alone recorded 74,680 new NHL cancer diagnoses and 19,910 NHL-related deaths in 2018. Meanwhile, the high burden of HIV infection in South Africa has contributed to an increased incidence of plasmablastic lymphoma (PBL).[6, 12]

For peer review only - http://bmjopen.bmj.com/site/about/guidelines.xhtml

Despite the strong association between HIV infection and NHL, the contribution of HIV to burden of NHL across time and region has not been comprehensively reported and quantified yet. Therefore, this study aims to perform a meta-analysis to quantify the association of HIV with NHL. Additionally, we intend to further estimate the degree of HIV contributing to burden of NHL using population attributable fraction (PAF) modeling analysis. The anticipated outcome of this study is expected to provide valuable insights for the development of targeted prevention and control strategies for NHL.

Objectives

This study aims to resolve questions:

1. Although HIV infection has a strong connection with NHL, there is no article quantifying this association comprehensively.

2. The contribution of HIV to burden of NHL across time and region has not been reported and quantified yet.

Methods and analysis

Our study will comprise two essential components as follows: 1) meta-analysis to quantify the association between HIV infection and NHL; 2) PAF modeling analysis to assess HIV-associated burden of NHL.

1. Meta-analysis to quantify the association between HIV infection and NHL

1.1 Registration

This protocol will follow to the guidelines of Preferred Reporting Items for Systematic reviews and Meta-Analyses for Protocols 2015 (PRISMA-P 2015)[13] and has registered on the PROSPERO (https://www.crd.york.ac.uk/PROSPERO/) (CRD 42023404150).

1.2 Outcomes definition, inclusion criteria and exclusion criteria

The main outcomes of this review are specific effect values and corresponding 95% confidence intervals (CIs) for NHL among individuals with HIV compared to those

For peer review only - http://bmjopen.bmj.com/site/about/guidelines.xhtml

without to quantify the association between HIV and NHL. Effect values that will be considered are risk ratio (RR), odds ratio (OR) and standardised incidence rate (SIR).

For inclusion criteria, our target design types are cohort study, case-control study, or cross-sectional study with effective control group. These studies need to expound the association between HIV and NHL using target effect value. In addition, eligible studies should provide detailed participant information, such as the total number of participants, total number of HIV patients, total number of control group, and number of NHL patients in case/control groups.

For exclusion criteria, we will exclude records whose types are review, opinion pieces, poster presentations, case reports or case series, and letters. If there is no targeted effect value or study population, this record will be also excluded. In addition, when exists overlap population in multiple articles, we will choose the one with larger study scale and higher article quality. As regards duplicate records, we will exclude incomplete or earlier period version.

1.3 Search strategy

We will search for English articles without restrictions of date or setting from four electronic databases: PubMed, Embase, Cochrane Library, and Web of Science up to 31 March 2023 with keywords "non-Hodgkin lymphoma", "human immunodeficiency virus", "Burkitt lymphoma", "follicular lymphoma", and "diffuse large B-cell lymphoma", (i.e., NHL, subtypes of NHL, HIV, and their equivalent terms). In addition, we will also screen references from eligible studies to ensure the integrity of the searches. The full electronic search strategies are available at the additional information (Appendix 1).

1.4 Data management and study selection

All retrieved records will be imported into EndNote X9.2 to manage and screen eligible studies. Research members will enact selection procedures and inclusion

For peer review only - http://bmjopen.bmj.com/site/about/guidelines.xhtml

criteria in advance all together.

Step 1: Duplicate record and article type screening

Two reviewers will initially utilize the built-in function of Endnote X9.2 to eliminate duplicate records. Subsequently, they will employ keyword searches within the software to exclude review and case reports. Due to the limitation of incomplete duplication checking function, certain duplicate records still need to be removed manually. Thus, we leverage the advantage of two-person independent operation to reduce the possibility of omissions. Following the manual deletion process, the two reviewers will cross-check the exclusion results to ensure the highest level of completeness in the final record selection.

Step 2: Title screening

Next, two authors will review the titles of the remaining records, excluding that does not involve HIV population or outcome of NHL. Moreover, these records that contain mice, mouse, review, case report, drug trial, or other non-target exposure and outcome will also be excluded based on our target study type and study subject.

Step 3: Abstract screening

In the abstract, if any record contains the target effect values and accords with desired causal association, it will proceed to the next screening phase. If there is any uncertainty or content deficiency, it is necessary to read the full text.

Step 4: Full text screening

The full text of remaining records will be downloaded from databases. Two authors will read full text throughout to reconfirm and extract data that can be utilized for statistical analyses. Articles will be ultimately included if they meet the eligibility criteria. Throughout the screening process, any discrepancy or conflict will be solved by consensus or arbitration from a third reviewer. Additionally, meticulous records will be maintained for each step of the screening processes, which will facilitate the

For peer review only - http://bmjopen.bmj.com/site/about/guidelines.xhtml

creation of a clear flow chart and the composition of formal article.

1.5 Data extraction

Two authors evaluate independently all retrieved articles by title, abstract, and full text according to inclusion and exclusion criteria. Solution of discrepancy or conflict will be same as the part of study selection. EndNote X9.2 will be used to manage the citations and extract data. The following data will be considered to extract (Table).

1.6 Quality assessment

All included studies will be assessed independently by two authors using an 11-item checklist recommended by the Agency for Healthcare Research and Quality (AHRQ) (https://www.wjx.cn/m/85284180.aspx). In this checklist, an item would be scored as '0' if the answer is 'NO' or 'UNCLEAR', and '1' if the answer is 'YES'. The determination of study quality will be complied with correspondence relationships as follows: low quality (0-3), moderate quality (4-7), high quality (8-11).[14] The judgement of studies' risk level will accord to the result of study quality assessment to divide into high, middle and low risk level corresponding to low, middle, high quality in sequence. Any disagreements between two authors will be resolved according to the method outlined in study selection process.

1.7 Statistical analysis

Overall statistical analyses and calculation will be completed by the R software (version 4.2.2). Firstly, we will calculate a pooled RR and corresponding 95% CI using function 'metagen' included in package 'meta' to quantify the association between HIV infection and NHL. Then the heterogeneity will be assessed using the chi-squared test on Cochrane's Q statistic, and the quantitation of heterogeneity will be measured by the Higgins's I^2 value presented as a percentage.[15] When the I^2 value exceeds 50% we will choose random-effect model, otherwise, a fixed-effect model will be used. If we can obtain sufficient data, subgroup analyses will be utilized to find the potential source of heterogeneity, including NHL subtype, study period,

For peer review only - http://bmjopen.bmj.com/site/about/guidelines.xhtml

population age, study conducted region, study design, setting of study (multi-center or single center), and article risk level. Furthermore, a meta-regression will be performed to ensure whether these factors can explain the heterogeneity and the degree they account for it. Regarding sensitivity analysis, we intend to analyse influence of single study by omitting one-by-one. And we remove studies with different risk levels to judge the robustness of the results. Moreover, we will use Egger's linear regression and funnel plot (if >10 individual studies) to find potential publication bias from these articles. If publication bias exists, we will measure it using the trim-and-fill method.

2. Population attributable fraction modeling analysis for HIV-associated burden of NHL estimation

2.1 Information sources

The number of age-specific disability-adjusted life year (DALY) will be obtained from the Global Health Data Exchange (GHDx) query tool (https://vizhub.healthdata.org/gbd-results/) in 5 years-old intervals. We will download national HIV prevalence data (≥15 years old) from 1990 to 2019 from the Joint United Nations Programme on HIV/AIDS (UNAIDS) (https://aidsinfo.unaids.org/). The criteria for dividing countries and regions will use UNAIDS classification and ISO codes alpha-3 (Appendix 2).

2.2 Population attributable fraction modeling implementation

Once we have obtained the pooled RR value based on meta-analysis, we will proceed with the PAF modeling to estimate the burden of HIV-associated NHL (including HIV-related DALY， and age-standardised DALY rate) according to the following steps.

Step 1: Population attributable fraction (PAF, %) calculation

We will calculate PAF combined with HIV prevalence data and pooled RR, with the following assumptions: 1) the pooled RR is applicable across the global, regions and all countries; 2) we ignore various confounding biases between primary studies,

For peer review only - http://bmjopen.bmj.com/site/about/guidelines.xhtml

which may cause slight deviation in our estimation; 3) we assume the pooled RR fits log normal distribution and beta distribution for the HIV prevalence, then we calculate 10,000 samples of PAF to obtain uncertainty intervals (2.5%, 97.5%) by R software. In practice, if eligible articles cover eight UNAIDS regions and have information on our planned subgroup analyses with sufficient data, we can calculate respective PAF with specific RR such as NHL subtype, study period, sex, or age. We will estimate the PAF using the Levin's formula [16]:

$$PAF = \frac{HIV\ prevalence * (RR - 1)}{1 + HIV\ prevalence * (RR - 1)}$$

Figure 1 illustrates the calculation process of PAF using South Africa as an example.

Step 2: Estimation of NHL disability-adjusted life year (DALY) related to HIV

We calculate age standardised rate (ASR) per 100,000 population of all-cause DALY and corresponding 95% CI by direct method, based on the age-specific all-cause NHL DALY and world standard population reported in the GBD 2019.[17] Formulas for calculating the ASR as below:

1)

$$Crude\ Rate = \frac{Case}{Population}$$

2)

$$Proportion = \frac{Age\ Group}{\sum Age\ Group}$$

3) for each age group: $Adjust\ Rate = Crude\ Rate * Proportion$

4) for aged ≥15 years old: $Adjust\ Rate = 100000 * sum\ (Adjust\ Rates)$

Then we assume that both number of DALY and ASR fit log normal distribution and we calculate 10,000 samples of HIV-associated DALY and ASR to obtain uncertainty interval (2.5%, 97.5%) by R software. We estimate the HIV-associated DALY number and ASR using formulas as below:

1) $DALY_{\text{attributed to HIV}} = All\_cause\ DALY * PAF$

2) $DALY\ ASR_{\text{attributed to HIV}} = All\_cause\ DALY\ ASR * PAF$

Figure 2 shows the calculation process of the HIV-associated NHL DALY for South Africa.

For peer review only - http://bmjopen.bmj.com/site/about/guidelines.xhtml

3. Patient and public involvement

No patient and public involved.

Discussion

The incidence rate of NHL exhibited a dramatic increase starting around 1970, and despite experiencing a plateau in between, it still recorded over 500 thousand of new cases in 2020.[4, 18] The complex etiology of NHL, coupled with the accelerated aging process of global population, is expected to further contributed to an increase of patients and deaths number for NHL.[6, 18] According to the UNAIDS, there are approximately 4000 new HIV infections reported daily, with 1100 of those are young people (aged from 15 to 24 years old).[19] Given to the close relationship between HIV and NHL, it is crucial to be concerned about the trajectory of the burden of HIV-associated NHL.

Our study aims to investigate the risk of NHL in individuals with HIV infection compared to those without HIV, and to quantify the contribution of HIV to incidence of NHL. To ensure the readability of the literature, we will only include articles published in English, which may limit the generalizability of our findings. In addition, it is also a limitation that we assume the pooled RR is applicable across global and regional areas. This is possible to lead to potential bias. Regarding the dissemination of our results, we will make every effort to publish them in a peer-reviewed journal as an academic article and present our findings at relevant field meetings. Since this review is based on published data, an ethical review is not required. Considering the global impact of HIV-associated NHL, we believe that conducting research on estimating the global burden of the disease can provide valuable insights for developing targeted prevention and control strategies, thereby achieving significant benefits.

Authors' contributions YC, YLY, ZWW, ZCS and PS designed the study protocol.

For peer review only - http://bmjopen.bmj.com/site/about/guidelines.xhtml

YC and ZCS prepared the first draft. YLY, ZWW and YPL reviewed and revised the first draft. All authors read and approved the final manuscript.

Funding statement This work was supported by [Science Foundation for Young Scholars of Sichuan Provincial People's Hospital] grant number [NO. 2022QN18] and [Subject Founds of Health Care for Cadres of Sichuan Province] grant number [NO. 2022-220].

Competing interests All authors declared that they had no competing interests.

Patient consent for publication Not required.

References

1. Sapkota S, Shaikh H: **Non-Hodgkin Lymphoma**. In: *StatPearls.* Treasure Island (FL) ineligible companies; 2023.
2. Swerdlow SH, Campo E, Pileri SA, Harris NL, Stein H, Siebert R, Advani R, Ghielmini M, Salles GA, Zelenetz AD *et al*: **The 2016 revision of the World Health Organization classification of lymphoid neoplasms**. *Blood* 2016, **127**(20):2375-2390. doi: 10.1182/blood-2016-01-643569
3. Thandra KC, Barsouk A, Saginala K, Padala SA, Barsouk A, Rawla P: **Epidemiology of Non-Hodgkin's Lymphoma**. *Med Sci (Basel)* 2021, **9**(1). doi: 10.3390/medsci9010005
4. Sung H, Ferlay J, Siegel RL, Laversanne M, Soerjomataram I, Jemal A, Bray F: **Global Cancer Statistics 2020: GLOBOCAN Estimates of Incidence and Mortality Worldwide for 36 Cancers in 185 Countries**. *CA Cancer J Clin* 2021, **71**(3):209-249. doi: 10.3322/caac.21660
5. Muller AM, Ihorst G, Mertelsmann R, Engelhardt M: **Epidemiology of non-Hodgkin's lymphoma (NHL): trends, geographic distribution, and etiology**. *Ann Hematol* 2005, **84**(1):1-12. doi: 10.1007/s00277-004-0939-7
6. Bispo JAB, Pinheiro PS, Kobetz EK: **Epidemiology and Etiology of Leukemia and Lymphoma**. *Cold Spring Harb Perspect Med* 2020, **10**(6). doi: 10.1101/cshperspect.a034819
7. Rana I, Dahlberg S, Steinmaus C, Zhang L: **Benzene exposure and non-Hodgkin lymphoma: a systematic review and meta-analysis of human studies**. *Lancet Planet Health* 2021, **5**(9):e633-e643. doi: 10.1016/S2542-5196(21)00149-2
8. Dal Maso L, Franceschi S: **Epidemiology of non-Hodgkin lymphomas and other haemolymphopoietic neoplasms in people with AIDS**. *Lancet Oncol* 2003, **4**(2):110-119. doi: 10.1016/s1470-2045(03)00983-5
9. Collaboration of Observational HIVERESG, Bohlius J, Schmidlin K, Costagliola D, Fatkenheuer G, May M, Caro-Murillo AM, Mocroft A, Bonnet F, Clifford G *et al*: **Incidence and risk factors of HIV-related non-Hodgkin's lymphoma in the era of combination antiretroviral therapy: a European multicohort study**. *Antivir Ther* 2009, **14**(8):1065-1074. doi: 10.3851/IMP1462

For peer review only - http://bmjopen.bmj.com/site/about/guidelines.xhtml

10. Jacobson LP, Yamashita TE, Detels R, Margolick JB, Chmiel JS, Kingsley LA, Melnick S, Munoz A: **Impact of potent antiretroviral therapy on the incidence of Kaposi's sarcoma and non-Hodgkin's lymphomas among HIV-1-infected individuals. Multicenter AIDS Cohort Study**. *J Acquir Immune Defic Syndr* 1999, **21 Suppl 1**:S34-41.

11. Polesel J, Clifford GM, Rickenbach M, Dal Maso L, Battegay M, Bouchardy C, Furrer H, Hasse B, Levi F, Probst-Hensch NM *et al*: **Non-Hodgkin lymphoma incidence in the Swiss HIV Cohort Study before and after highly active antiretroviral therapy**. *AIDS* 2008, **22**(2):301-306. doi: 10.1097/QAD.0b013e3282f2705d

12. Vaughan J, Perner Y, Mayne E, Wiggill T: **Plasmablastic lymphoma in Johannesburg, South Africa, in the era of widescale antiretroviral therapy use**. *HIV Med* 2021, **22**(3):225-230. doi: 10.1111/hiv.12965

13. Moher D, Shamseer L, Clarke M, Ghersi D, Liberati A, Petticrew M, Shekelle P, Stewart LA, Group P-P: **Preferred reporting items for systematic review and meta-analysis protocols (PRISMA-P) 2015 statement**. *Syst Rev* 2015, **4**(1):1. doi: 10.1186/2046-4053-4-1

14. Hu J, Dong Y, Chen X, Liu Y, Ma D, Liu X, Zheng R, Mao X, Chen T, He W: **Prevalence of suicide attempts among Chinese adolescents: A meta-analysis of cross-sectional studies**. *Compr Psychiatry* 2015, **61**:78-89. doi: 10.1016/j.comppsych.2015.05.001

15. Higgins JP, Thompson SG: **Quantifying heterogeneity in a meta-analysis**. *Stat Med* 2002, **21**(11):1539-1558. doi: 10.1002/sim.1186

16. Khosravi A, Nielsen RO, Mansournia MA: **Methods matter: population attributable fraction (PAF) in sport and exercise medicine**. *Br J Sports Med* 2020, **54**(17):1049-1054. doi: 10.1136/bjsports-2020-101977

17. Collaborators GBDD: **Global age-sex-specific fertility, mortality, healthy life expectancy (HALE), and population estimates in 204 countries and territories, 1950-2019: a comprehensive demographic analysis for the Global Burden of Disease Study 2019**. *Lancet* 2020, **396**(10258):1160-1203. doi: 10.1016/S0140-6736(20)30977-6

18. Skrabek P, Turner D, Seftel M: **Epidemiology of non-Hodgkin lymphoma**. *Transfus Apher Sci* 2013, **49**(2):133-138. doi: 10.1016/j.transci.2013.07.014

19. UNAIDS. IN DANGER: UNAIDS Global AIDS Update 2022: UNAIDS; 2022 [cited 2023 14 May]. Available from: https://www.unaids.org/en/resources/documents/2022/in-danger-global-aids-update.

For peer review only - http://bmjopen.bmj.com/site/about/guidelines.xhtml

Table. Data to be extracted from eligible articles.

Category	Data
Publication details	Author, publication year
Type of design	Cohort study, CCS, or cross-sectional study with effective control group
Information of study	Study period, NHL subtype, country and region of study conducted, study setting (multi-center or single center), disease classification criterion, data source or name of cohort registered, median patient age
Effect values	RR, OR and SIR and their corresponding 95% CI
Study participants	Total number of HIV patients, total number of control group, number of NHL in the case/control group

Abbreviations: CCS-Case-control study; NHL-Non-Hodgkin lymphoma, RR-Risk ratio; OR-Odds ratio; SIR-Standardised incidence rate; CI-Confidence interval; HIV-Human immunodeficiency virus

Figure legends:

Figure 1. Example procedure for calculating PAF in South Africa

Abbreviations: PAF-Population attributable fraction

Figure 2. Example procedure for calculating the HIV-associated NHL DALY in South Africa

Abbreviations: HIV-Human immunodeficiency virus; NHL-Non-Hodgkin lymphoma; DALY-Disability-adjusted life year

For peer review only

For peer review only - http://bmjopen.bmj.com/site/about/guidelines.xhtml

South Africa in 2000:

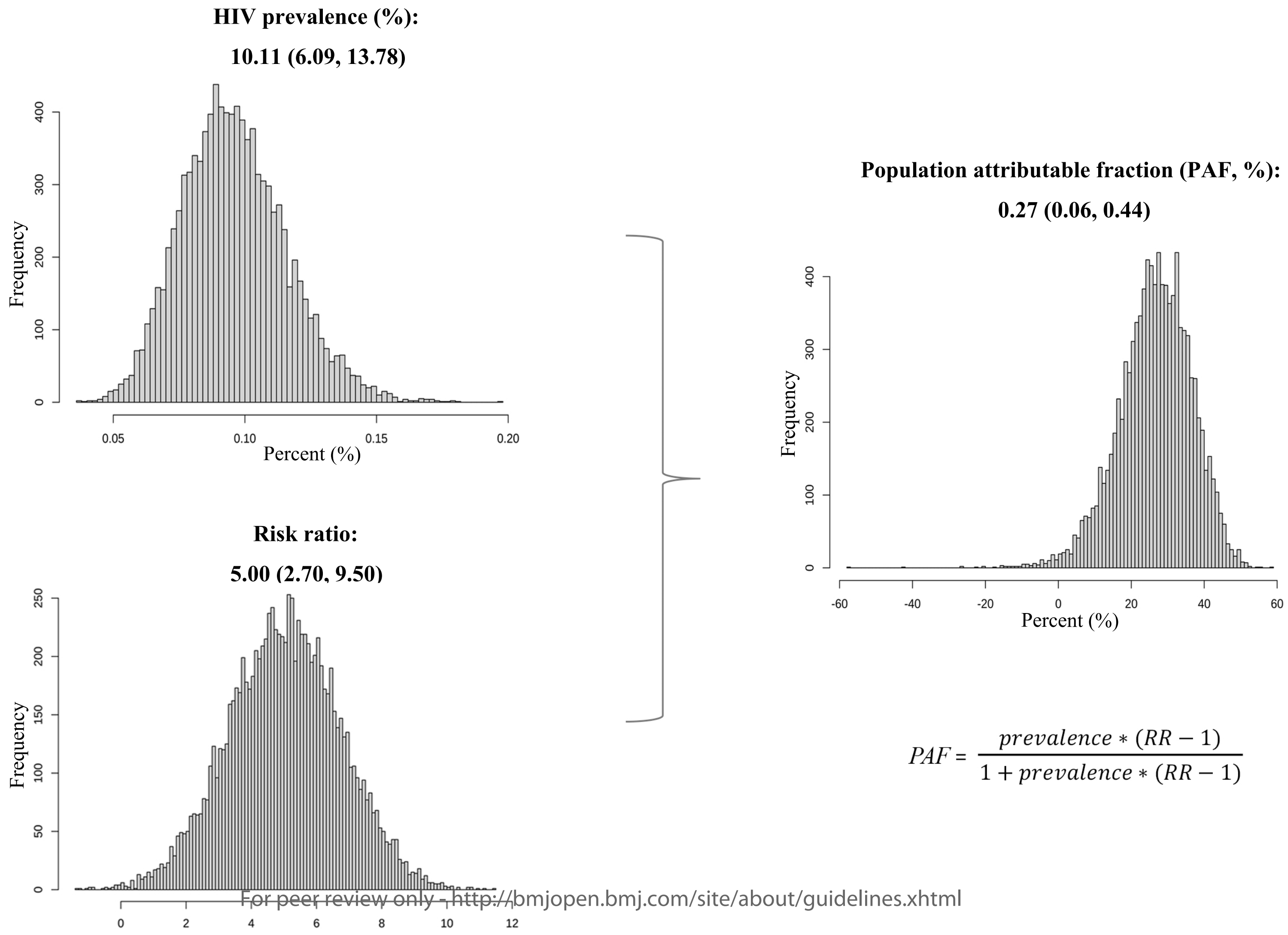

For peer review only - http://bmjopen.bmj.com/site/about/guidelines.xhtml

South Africa in 2000:

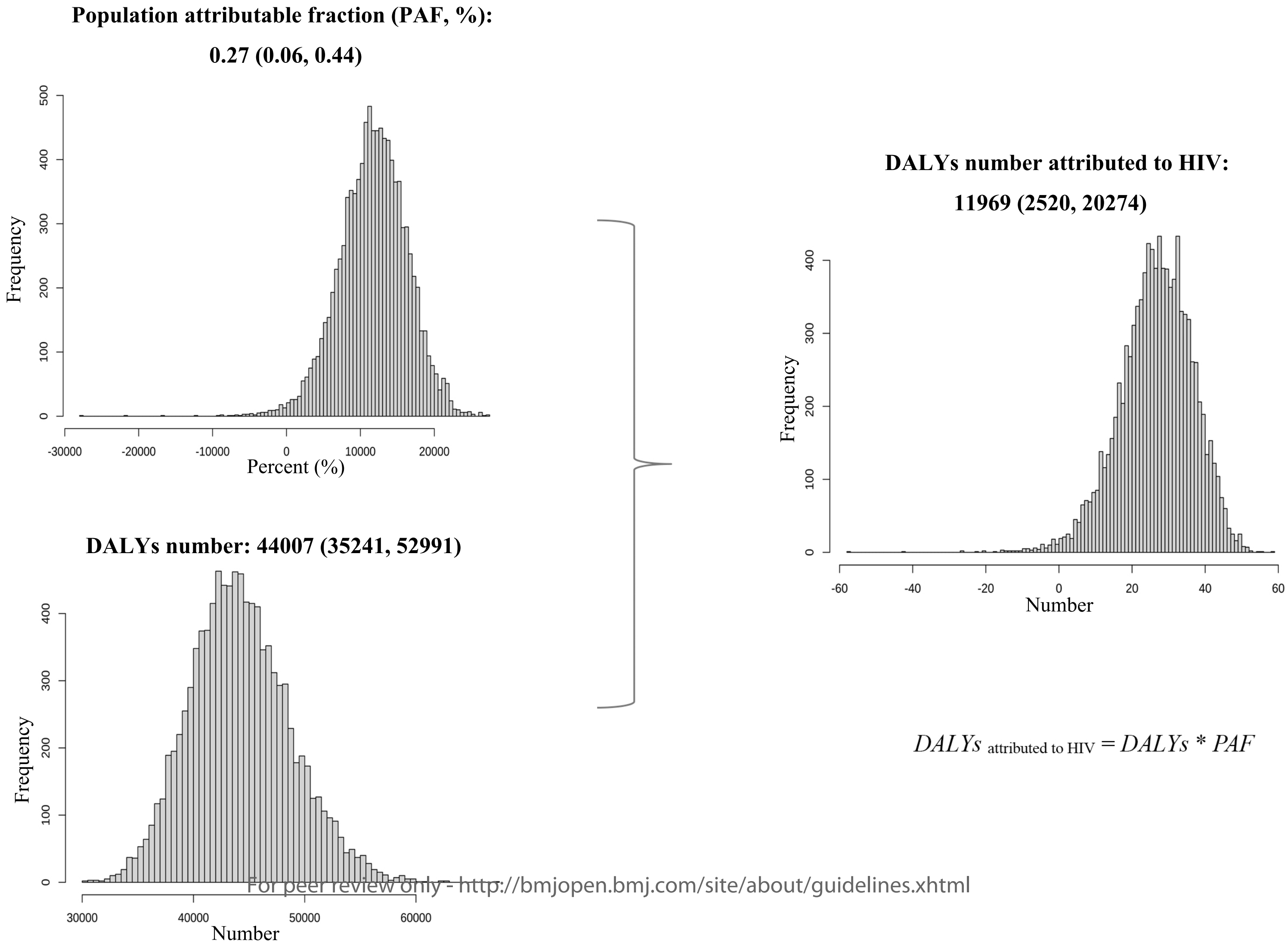

For peer review only - http://bmjopen.bmj.com/site/about/guidelines.xhtml

Appendix

Appendix 1. Electronic databases search strategies in PubMed, Embase, Cochrane Library and Web of Science

PubMed

Set	Search	Results
#1	"lymphoma, non hodgkin"[MeSH Terms]	
#2	"hiv"[Title/Abstract] OR "hiv-1"[Title/Abstract] OR "hiv 1"[Title/Abstract] OR "hiv 2"[Title/Abstract] OR "hiv-2"[Title/Abstract] OR "human immunodeficiency virus"[Title/Abstract] OR "human immuno deficiency virus"[Title/Abstract] OR "human immunedeficiency virus"[Title/Abstract] OR "human immune deficiency virus"[Title/Abstract] OR "acquired immunodeficiency syndrome"[MeSH Terms] OR "acquired immunodeficiency syndrome"[Title/Abstract] OR "acquired immuno-deficiency syndrome"[Title/Abstract]OR "acquired immunedeficiency syndrome"[Title/Abstract]	
#3	"lymphoma non hodgkin"[Title/Abstract] OR (("Lymphoma"[MeSH Terms] OR "Lymphoma"[All Fields] OR "Lymphomas"[All Fields] OR "lymphoma s"[All Fields]) AND "small cleaved cell diffuse"[Title/Abstract]) OR ((("Lymphoma"[MeSH Terms] OR "Lymphoma"[All Fields] OR "Lymphomas"[All Fields] OR "lymphoma s"[All Fields]) AND ("atypic"[All Fields] OR "atypical"[All Fields] OR "atypicalities"[All Fields] OR "atypicality"[All Fields] OR "atypically"[All Fields] OR "atypicals"[All Fields] OR "atypism"[All Fields] OR "atypisms"[All Fields]) AND ("diffusable"[All Fields] OR "diffusant"[All Fields] OR "diffusants"[All Fields] OR "Diffuse"[All Fields] OR "diffusely"[All Fields] OR "diffuses"[All Fields] OR "diffusibility"[All Fields] OR "diffusible"[All Fields] OR "diffusion"[MeSH Terms] OR "diffusion"[All Fields] OR "diffused"[All Fields] OR "diffusing"[All Fields] OR "diffusions"[All Fields] OR "diffusive"[All Fields] OR "diffusively"[All Fields] OR "diffusivities"[All Fields] OR "diffusivity"[All Fields])) AND "small lymphoid"[Title/Abstract]) OR "diffuse small cleaved cell lymphoma"[Title/Abstract] OR "diffuse small cleaved cell lymphoma"[Title/Abstract] OR (("Lymphoma"[MeSH Terms] OR "Lymphoma"[All Fields] OR "Lymphomas"[All Fields] OR "lymphoma s"[All Fields]) AND "small cleaved cell diffuse"[Title/Abstract]) OR (("Lymphoma"[MeSH Terms] OR "Lymphoma"[All Fields] OR "Lymphomas"[All Fields] OR "lymphoma s"[All Fields]) AND "Nonhodgkins"[Title/Abstract]) OR "nonhodgkins lymphoma"[Title/Abstract] OR "lymphoma non hodgkins"[Title/Abstract] OR "lymphoma non hodgkins"[Title/Abstract] OR "non hodgkins lymphoma"[Title/Abstract] OR "non hodgkin lymphoma"[Title/Abstract] OR "non hodgkin lymphoma"[Title/Abstract] OR "lymphoma nonhodgkin s"[Title/Abstract] OR	

For peer review only

	"lymphoma nonhodgkin"[Title/Abstract] OR "small cleaved cell lymphoma diffuse"[Title/Abstract] OR "small cleaved cell lymphoma diffuse"[Title/Abstract] OR "lymphoma non hodgkin s"[Title/Abstract] OR "lymphoma non hodgkin s"[Title/Abstract] OR "lymphoma diffuse"[Title/Abstract] OR "diffuse lymphoma"[Title/Abstract] OR "diffuse lymphomas"[Title/Abstract] OR "sarcoma lymphatic"[Title/Abstract] OR "Lymphosarcoma"[Title/Abstract] OR "Lymphosarcomas"[Title/Abstract] OR "lymphatic sarcoma"[Title/Abstract] OR "lymphatic sarcomas"[Title/Abstract] OR "lymphoma low grade"[Title/Abstract] OR "low grade lymphoma"[Title/Abstract] OR "low grade lymphomas"[Title/Abstract] OR "lymphoma low grade"[Title/Abstract] OR "lymphoma mixed cell"[Title/Abstract] OR "lymphoma mixed cell"[Title/Abstract] OR "mixed cell lymphoma"[Title/Abstract] OR "mixed cell lymphoma"[Title/Abstract] OR "mixed cell lymphomas"[Title/Abstract] OR "lymphoma mixed"[Title/Abstract] OR "mixed lymphoma"[Title/Abstract] OR "mixed lymphomas"[Title/Abstract] OR "lymphoma mixed lymphocytic histiocytic"[Title/Abstract] OR ("Lymphocytic-Histiocytic"[All Fields] AND "lymphoma mixed"[Title/Abstract]) OR ("Lymphocytic-Histiocytic"[All Fields] AND "lymphomas mixed"[Title/Abstract]) OR "lymphoma mixed lymphocytic histiocytic"[Title/Abstract] OR "mixed lymphocytic histiocytic lymphoma"[Title/Abstract] OR "mixed lymphocytic histiocytic lymphomas"[Title/Abstract] OR (("lymphoma, non hodgkin"[MeSH Terms] OR ("Lymphoma"[All Fields] AND "Non-Hodgkin"[All Fields]) OR "non-hodgkin lymphoma"[All Fields] OR ("Lymphoma"[All Fields] AND "Non"[All Fields] AND "Hodgkin"[All Fields]) OR "lymphoma non hodgkin"[All Fields]) AND "Familial"[Title/Abstract]) OR "lymphoma small noncleaved cell"[Title/Abstract] OR "lymphoma small noncleaved cell"[Title/Abstract] OR "small noncleaved cell lymphoma"[Title/Abstract] OR ("Noncleaved-Cell"[All Fields] AND "lymphoma small"[Title/Abstract]) OR "small noncleaved cell lymphoma"[Title/Abstract] OR "small noncleaved cell lymphomas"[Title/Abstract] OR "diffuse undifferentiated lymphoma"[Title/Abstract] OR "diffuse undifferentiated lymphomas"[Title/Abstract] OR "lymphoma diffuse undifferentiated"[Title/Abstract] OR "undifferentiated lymphoma diffuse"[Title/Abstract] OR "lymphoma small non cleaved cell"[Title/Abstract] OR "lymphoma small non cleaved cell"[Title/Abstract] OR ("Non-Cleaved-Cell"[All Fields] AND "lymphoma small"[Title/Abstract]) OR "small non cleaved cell lymphomas"[Title/Abstract] OR "small non cleaved cell lymphoma"[Title/Abstract] OR "small non cleaved cell lymphoma"[Title/Abstract] OR (("Lymphoma"[MeSH Terms] OR "Lymphoma"[All Fields] OR "Lymphomas"[All Fields] OR "lymphoma s"[All Fields]) AND "undifferentiated diffuse"[Title/Abstract]) OR "lymphoma intermediate grade"[Title/Abstract] OR "intermediate grade lymphoma"[Title/Abstract] OR "intermediate grade lymphomas"[Title/Abstract]	

For peer review only

	OR "lymphoma intermediate grade"[Title/Abstract] OR "lymphoma undifferentiated"[Title/Abstract] OR "lymphoma pleomorphic"[Title/Abstract] OR "pleomorphic lymphoma"[Title/Abstract] OR "diffuse mixed cell lymphomas"[Title/Abstract] OR "diffuse mixed cell lymphoma"[Title/Abstract] OR "diffuse mixed cell lymphoma"[Title/Abstract] OR "non hodgkin s lymphoma"[Title/Abstract] OR "non hodgkin s lymphoma"[Title/Abstract] OR "nonhodgkin s lymphoma"[Title/Abstract] OR ("diffuse mixed small"[Title/Abstract] AND "large cell lymphoma"[Title/Abstract]) OR (("Mixed"[All Fields] OR "mixes"[All Fields] OR "mixing"[All Fields] OR "mixings"[All Fields]) AND "cell lymphoma diffuse"[Title/Abstract]) OR ("Mixed-Cell"[All Fields] AND "lymphoma diffuse"[Title/Abstract]) OR (("Lymphoma"[MeSH Terms] OR "Lymphoma"[All Fields] OR "Lymphomas"[All Fields] OR "lymphoma s"[All Fields]) AND "mixed cell diffuse"[Title/Abstract]) OR ("lymphoma small"[Title/Abstract] AND ((("Large"[All Fields] OR "largely"[All Fields] OR "larges"[All Fields]) AND "Cleaved-Cell"[All Fields]) AND "Diffuse"[Title/Abstract])) OR (("lymphoma, non hodgkin"[MeSH Terms] OR ("Lymphoma"[All Fields] AND "Non-Hodgkin"[All Fields]) OR "non-hodgkin lymphoma"[All Fields] OR ("Lymphoma"[All Fields] AND "Diffuse"[All Fields]) OR "lymphoma diffuse"[All Fields]) AND "mixed lymphocytic histiocytic"[Title/Abstract]) OR ("lymphoma mixed small"[Title/Abstract] AND "large cell diffuse"[Title/Abstract]) OR ("mixed small"[Title/Abstract] AND "large cell lymphoma diffuse"[Title/Abstract]) OR (("Lymphoma"[MeSH Terms] OR "Lymphoma"[All Fields] OR "Lymphomas"[All Fields] OR "lymphoma s"[All Fields]) AND "mixed cell diffuse"[Title/Abstract]) OR "lymphoma high grade"[Title/Abstract] OR "high grade lymphomas"[Title/Abstract] OR "high grade lymphoma"[Title/Abstract] OR "lymphoma high grade"[Title/Abstract] OR "Reticulosarcomas"[Title/Abstract] OR "Reticulosarcoma"[Title/Abstract] OR "reticulum cell sarcomas"[Title/Abstract] OR "reticulum cell sarcoma"[Title/Abstract] OR "sarcoma reticulum cell"[Title/Abstract] OR "sarcoma reticulum cell"[Title/Abstract] OR "reticulum cell sarcomas"[Title/Abstract] OR "reticulum cell sarcoma"[Title/Abstract] OR "undifferentiated lymphomas"[Title/Abstract] OR "undifferentiated lymphoma"[Title/Abstract] OR "pleomorphic lymphomas"[Title/Abstract]	
#4	"prevalence"[Text Word] OR "incidence"[Text Word] OR "epidemiol*"[Text Word] OR "survey"[Text Word] OR "rapid assessment"[Text Word] OR "situation assessment"[Text Word] OR "situational assessment"[Text Word] OR "rar"[Text Word] OR "cohort"[Text Word] OR "surveillance"[Text Word] OR "seroprevalence"[Text Word] OR "seroincidence"[Text Word] OR "screening"[Text Word] OR "mortality"[Title/Abstract] OR "mortalities"[Title/Abstract] OR "mortal"[Title/Abstract] OR "dead"[Title/Abstract] OR "death"[Title/Abstract] OR "deaths"[Title/Abstract] OR "decease*"[Title/Abstract] OR "demise*"[Title/Abstract] OR "die"[Title/Abstract] OR "died"[Title/Abstract] OR "dies"[Title/Abstract] OR	

For peer review only

	"dying"[Title/Abstract] OR "fatal"[Title/Abstract] OR "fatality"[Title/Abstract] OR "fatalities"[Title/Abstract] OR "nonsurvival*"[Title/Abstract] OR "nonsurvival*"[Title/Abstract] OR "survival*"[Title/Abstract] OR "lethality"[Title/Abstract] OR "epidemiologic methods"[MeSH Major Topic] OR "epidemiologic studies"[MeSH Major Topic] OR "sentinel surveillance"[MeSH Major Topic] OR "seroepidemiologic studies"[MeSH Major Topic] OR "cohort studies"[MeSH Major Topic] OR "longitudinal studies"[MeSH Major Topic] OR "follow up studies"[MeSH Major Topic] OR "prospective studies"[MeSH Major Topic] OR "diagnostic services"[MeSH Major Topic] OR "mass screening"[MeSH Major Topic] OR "mortality"[MeSH Major Topic] OR "hospital mortality"[MeSH Major Topic] OR "death"[MeSH Major Topic] OR "cause of death"[MeSH Major Topic] OR "death certificates"[MeSH Major Topic] OR "fatal outcome"[MeSH Major Topic] OR "autopsy"[MeSH Major Topic] OR "survival rate"[MeSH Major Topic]	
#5	#1 OR #2	
#6	#3 and #4 and #5	

Embase

Set	Search	Results
#1	'human immunodeficiency virus infection'/exp OR 'human immunodeficiency virus'/exp OR 'hiv survivor'/exp OR hiv:ti,ab,kw OR 'hiv-1':ti,ab,kw OR 'hiv-2':ti,ab,kw OR 'human immunodeficiency virus':ti,ab,kw OR 'human immunodeficiency':ti,ab,kw OR 'human immunedeficiency virus':ti,ab,kw OR 'human immune deficiency virus':ti,ab,kw OR 'human immune-deficiency virus':ti,ab,kw OR 'acquired immune-deficiency syndrome':ti,ab,kw OR 'acquired immunedeficiency syndrome':ti,ab,kw OR 'acquired immunodeficiency syndrome':ti,ab,kw OR 'acquired immuno-deficiency syndrome':ti,ab,kw	
#2	prevalence:ti,ab,de OR incidence:ti,ab,de OR epidemiol*:ti,ab,de OR survey:ti,ab,de OR 'rapid assessment':ti,ab,de OR 'situation assessment':ti,ab,de OR 'situational assessment':ti,ab,de OR rar:ti,ab,de OR cohort:ti,ab,de OR surveillance:ti,ab,de OR seroprevalence:ti,ab,de OR seroincidence:ti,ab,de OR seroepidemiol*:ti,ab,de OR screening:ti,ab,de OR 'mass screening':ti,ab,de OR mortality:ti,ab OR mortalities:ti,ab OR mortal:ti,ab OR dead:ti,ab OR death:ti,ab OR deaths:ti,ab OR decease*:ti,ab OR demise*:ti,ab OR die:ti,ab OR died:ti,ab OR dies:ti,ab OR dying:ti,ab OR fatal:ti,ab OR fatality:ti,ab OR fatalities:ti,ab OR 'non survival*':ti,ab OR nonsurvival*:ti,ab OR survival*:ti,ab OR lethality:ti,ab OR epidemiology:de OR 'cancer epidemiology'/exp OR 'cancer statistics'/exp OR 'disease surveillance'/exp OR 'health survey'/exp OR 'incidence'/exp OR 'infection rate'/exp OR 'prevelance' OR 'sentinel surveillance'/exp OR 'seroepidemiology'/exp OR 'cohort analysis'/exp OR 'longitudinal study'/exp OR 'prospective study'/exp OR 'preventive health service'/exp OR 'mass	

	screening'/exp OR 'cancer mortality'/exp OR 'all cause mortality'/exp OR 'mortality':de OR 'hospital mortality'/exp OR 'mortality rate'/exp OR 'death'/exp OR 'cause of death'/exp OR 'dying'/exp OR 'fatality'/exp OR 'lethality'/exp OR 'death certificate'/exp OR 'autopsy'/exp OR 'cancer survival'/exp OR 'survival rate'/exp	
#3	'non-hodgkin lymphoma'/exp	
#4	'lymphoma, non hodgkin':ab,ti	
#5	'lymphoma, small cleaved-cell, diffuse':ab,ti	
#6	'lymphoma, atypical diffuse small lymphoid':ab,ti	
#7	'diffuse small cleaved-cell lymphoma':ab,ti	
#8	'diffuse small cleaved cell lymphoma':ab,ti	
#9	'lymphoma, small cleaved cell, diffuse':ab,ti	
#10	'lymphoma, nonhodgkins':ab,ti	
#11	'nonhodgkins lymphoma':ab,ti	
#12	'lymphoma, non-hodgkins':ab,ti	
#13	'lymphoma, non hodgkins':ab,ti	
#14	'lymphoma, non hodgkins':ab,ti	
#15	'non-hodgkins lymphoma':ab,ti	
#16	'non-hodgkin lymphoma':ab,ti	
#17	'lymphoma, nonhodgkin':ab,ti	
#18	'small cleaved-cell lymphoma, diffuse':ab,ti	
#19	'lymphoma, diffuse':ab,ti	
#20	'ldiffuse lymphomae':ab,ti	
#21	'diffuse lymphomae':ab,ti	
#22	'diffuse lymphomas':ab,ti	
#23	'diffuse lymphoma':ab,ti	
#24	'sarcoma, lymphatic':ab,ti	
#25	'lymphosarcoma':ab,ti	
#26	'lymphosarcomas':ab,ti	
#27	'llymphatic sarcomas':ab,ti	
#28	'lymphatic sarcomas':ab,ti	
#29	'lymphatic sarcomas':ab,ti	
#30	'lymphoma, low-grade':ab,ti	
#31	'low-grade lymphoma':ab,ti	
#32	'low-grade lymphomas':ab,ti	
#33	'lymphoma, low grade':ab,ti	
#34	'lymphoma, mixed-cell':ab,ti	
#35	'lymphoma, mixed cell':ab,ti	
#36	'mixed-cell lymphoma':ab,ti	
#37	'mixed cell lymphoma':ab,ti	
#38	'mixed-cell lymphomas':ab,ti	
#39	'lymphoma, mixed':ab,ti	
#40	'mixed lymphoma':ab,ti	

For peer review only

#41	'lymphoma, mixed lymphocytic-histiocytic':ab,ti	
#42	'lymphocytic-histiocytic lymphoma, mixed':ab,ti	
#43	'mixed lymphocytic-histiocytic lymphoma':ab,ti	
#44	'mixed lymphocytic-histiocytic lymphomas':ab,ti	
#45	'lymphoma, non-hodgkin, familial':ab,ti	
#46	'lymphoma, small noncleaved-cell':ab,ti	
#47	'lymphoma, small noncleaved cell':ab,ti	
#48	'small noncleaved-cell lymphoma':ab,ti	
#49	'noncleaved-cell lymphoma, small':ab,ti	
#50	'small noncleaved cell lymphoma':ab,ti	
#51	'small noncleaved-cell lymphomas':ab,ti	
#52	'diffuse undifferentiated lymphoma':ab,ti	
#53	'diffuse undifferentiated lymphomas':ab,ti	
#54	#3 OR #4 OR #5 OR #6 OR #7 OR #8 OR #9 OR #10 OR #11 OR #12 OR #13 OR #14 OR #15 OR #16 OR #17 OR #18 OR #19 OR #20 OR #21 OR #22 OR #23 OR #24 OR #25 OR #26 OR #27 OR #28 OR #29 OR #30 OR #31 OR #32 OR #33 OR #34 OR #35 OR #36 OR #37 OR #38 OR #39 OR #40 OR #41 OR #42 OR #43 OR #44 OR #45 OR #46 OR #47 OR #48 OR #49 OR #50 OR #51 OR #52 OR #53	
#55	#1 AND #2 AND #54	

Cochrane Library

Set	Search	Results
#1	MeSH descriptor: [HIV] explode all trees	
#2	(hiv):ti,ab,kw (Word variations have been searched)	
#3	#1 OR #2	
#4	("non-Hodgkin lymphoma"):ti,ab,kw (Word variations have been searched)	
#5	MeSH descriptor: [Lymphoma, Non-Hodgkin] explode all trees	
#6	#4 OR #5	
#7	#6 OR #3	

Web of Science

Set	Search	Results
#1	"TS=(prevalence OR incidence OR epidemiol* OR survey OR ""rapid assessment"" OR ""situation assessment"" OR ""situational assessment"" OR rar OR cohort OR surveillance OR seroprevalence OR seroincidence OR seroepidemiol* OR screening OR mortality OR mortalities OR mortal OR dead OR death OR deaths OR decease* OR demise* OR die OR died OR dies OR dying OR fatal OR fatality OR fatalities OR non-survival* OR Nonsurvival* OR survival* OR lethality OR ""longitudinal studies"" OR ""follow-up studies"" OR ""prospective studies"" OR ""Mass Screening"" OR	

For peer review only

	""Autopsy"")	
#2	"TS=("hiv" OR "hiv-1" OR "hiv-2" OR "hiv1" OR "hiv2" OR hiv infect* OR "human immunodeficiency virus" OR "human immunedeficiency virus" OR "human immuno-deficiency virus" OR "human immune-deficiency virus" OR ((human immun*) AND ("deficiency virus")) OR "acquired immunodeficiency syndrome" OR "acquired immunedeficiency syndrome" OR "acquired immuno-deficiency syndrome" OR "acquired immune-deficiency syndrome" OR ((acquired immun*) AND ("deficiency syndrome")))	
#3	"TS=("Non-Hodgkin lymphoma" OR "Lymphoma, Non Hodgkin" OR "Lymphoma, Small Cleaved-Cell, Diffuse" OR "Lymphoma, Atypical Diffuse Small Lymphoid" OR "Diffuse Small Cleaved-Cell Lymphoma" OR "Diffuse Small Cleaved Cell Lymphoma" OR "Lymphoma, Small Cleaved Cell, Diffuse" OR "Lymphoma, Nonhodgkins" OR "Nonhodgkins Lymphoma" OR "Lymphoma, Non-Hodgkins" OR "Lymphoma, Non Hodgkins" OR "Non-Hodgkins Lymphoma" OR "Non-Hodgkin Lymphoma" OR "Non Hodgkin Lymphoma" OR "Lymphoma, Nonhodgkin's" OR "Lymphoma, Nonhodgkin" OR "Small Cleaved-Cell Lymphoma, Diffuse" OR "Small Cleaved Cell Lymphoma, Diffuse" OR "Lymphoma, Non-Hodgkin's" OR "Lymphoma, Non Hodgkin's" OR "Lymphoma, Diffuse" OR "Diffuse Lymphoma" OR "Diffuse Lymphomas" OR "Sarcoma, Lymphatic" OR "Lymphosarcoma" OR "Lymphosarcomas" OR "Lymphatic Sarcoma" OR "Lymphatic Sarcomas" OR "Lymphoma, Low-Grade" OR "Low-Grade Lymphoma" OR "Low-Grade Lymphomas" OR "Lymphoma, Low Grade" OR "Lymphoma, Mixed-Cell" OR "Lymphoma, Mixed Cell" OR "Mixed-Cell Lymphoma" OR "Mixed Cell Lymphoma" OR "Mixed-Cell Lymphomas" OR "Lymphoma, Mixed" OR "Mixed Lymphoma" OR "Mixed Lymphomas" OR "Lymphoma, Mixed Lymphocytic-Histiocytic"OR "Lymphocytic-Histiocytic Lymphoma, Mixed" OR "Lymphocytic-Histiocytic Lymphomas, Mixed" OR "Lymphoma, Mixed Lymphocytic Histiocytic" OR "Mixed Lymphocytic-Histiocytic Lymphoma" OR "Mixed Lymphocytic-Histiocytic Lymphomas" OR "Lymphoma, Non-Hodgkin, Familial" OR "Lymphoma, Small Noncleaved-Cell" OR "Lymphoma, Small Noncleaved Cell" OR "Small Noncleaved-Cell Lymphoma" OR "Noncleaved-Cell Lymphoma, Small" OR "Small Noncleaved Cell Lymphoma" OR "Small Noncleaved-Cell Lymphomas" OR "Diffuse Undifferentiated Lymphoma" OR "Diffuse Undifferentiated Lymphomas" OR "Lymphoma, Diffuse Undifferentiated" OR "Undifferentiated Lymphoma, Diffuse" OR "Lymphoma, Small Non-Cleaved-Cell" OR "Lymphoma, Small Non Cleaved Cell" OR "Non-Cleaved-Cell Lymphoma, Small" OR "Small Non-Cleaved-Cell Lymphomas" OR "Small Non-Cleaved-Cell Lymphoma" OR "Small Non Cleaved Cell Lymphoma" OR "Lymphoma, Undifferentiated, Diffuse" OR "Lymphoma, Intermediate-Grade" OR "Intermediate-Grade Lymphoma" OR "Intermediate-Grade Lymphomas" OR "Lymphoma,	

For peer review only

	Intermediate Grade" OR "Lymphoma, Undifferentiated" OR "Lymphoma, Pleomorphic" OR "Pleomorphic Lymphoma" OR "Pleomorphic Lymphomas" OR "Undifferentiated Lymphoma" OR "Undifferentiated Lymphomas" OR "Reticulum-Cell Sarcoma" OR "Reticulum-Cell Sarcomas" OR "Sarcoma, Reticulum-Cell" OR "Sarcoma, Reticulum Cell" OR "Reticulum Cell Sarcoma" OR "Reticulum Cell Sarcomas" OR "Reticulosarcoma" OR "Reticulosarcomas" OR "Lymphoma, High-Grade" OR "High-Grade Lymphoma" OR "High-Grade Lymphomas" OR "Lymphoma, High Grade" OR "Lymphoma, Mixed-Cell, Diffuse" OR "Mixed Small and Large Cell Lymphoma, Diffuse" OR "Lymphoma, Mixed Small and Large Cell, Diffuse" OR "Lymphoma, Diffuse, Mixed Lymphocytic-Histiocytic" OR "Lymphoma, Small and Large Cleaved-Cell, Diffuse" OR "Lymphoma, Mixed Cell, Diffuse" OR "Mixed-Cell Lymphoma, Diffuse" OR "Mixed Cell Lymphoma, Diffuse" OR "Diffuse Mixed Small and Large Cell Lymphoma" OR "Diffuse Mixed-Cell Lymphoma" OR "Diffuse Mixed Cell Lymphoma" OR "Diffuse Mixed-Cell Lymphomas" OR "Lymphoma, Diffuse Mixed-Cel" OR "Non-Hodgkin's Lymphoma" OR "Non Hodgkin's Lymphoma" OR "Nonhodgkin's Lymphoma")	
#4	#1 AND #2 AND #3	

For peer review only

Appendix 2. Countries and regions by UNAIDS

UNAIDS Regions	Country	ISO Code	Unaids Regions	Country	ISO Code
**Global**	—	03M49WLD	**Latin America**	Argentina	ARG
**Asia and the Pacific**	Afghanistan	AFG		Bolivia	BOL
	Australia	AUS		Brazil	BRA
	Bangladesh	BGD		Chile	CHL
	Bhutan	BTN		Colombia	COL
	Brunei	BRN		Costa Rica	CRI
	Cambodia	KHM		Ecuador	ECU
	China	CHN		El Salvador	SLV
	South Korea	PRK		Guatemala	GTM
	Micronesia	FSM		Honduras	HND
	Fiji	FJI		Mexico	MEX
	India	IND		Nicaragua	NIC
	Indonesia	IDN		Panama	PAN
	Japan	JPN		Paraguay	PRY
	Kiribati	KIR		Peru	PER
	Laos	LAO		Uruguay	URY
	Malaysia	MYS		Venezuela	VEN
	Maldives	MDV	**Middle East and North Africa**	Algeria	DZA
	Marshall Islands	MHL		Bahrain	BHR
	Mongolia	MNG		Djibouti	DJI
	Myanmar	MMR		Egypt	EGY
	Nauru	NRU		Iraq	IRQ

For peer review only

	Nepal	NPL		Iran	IRN
	New Zealand	NZL		Jordan	JOR
	Pakistan	PAK		Kuwait	KWT
	Palau	PLW		Lebanon	LBN
	Papua New Guinea	PNG		Libya	LBY
	Philippines	PHL		Morocco	MAR
	North Korea	KOR		Oman	OMN
	Samoa	WSM		Qatar	QAT
	Singapore	SGP		Saudi Arabia	SAU
	Solomon Islands	SLB		Somalia	SOM
	Sri Lanka	LKA		Sudan	SDN
	Thailand	THA		Syria	SYR
	Timor-Leste	TLS		Tunisia	TUN
	Tonga	TON		United Arab Emirates	ARE
	Tuvalu	TUV		Yemen	YEM
	Vanuatu	VUT	**Western and central Africa**	Benin	BEN
	Vietnam	VNM		Burkina Faso	BFA
**Caribbean**	Antigua	ATG		Burundi	BDI
	Bahamas	BMH		Ivory Coast	CIV
	Barbados	BRB		Cameroon	CMR
	Belize	BLZ		Cape Verde	CPV
	Cuba	CUB		Central African Republic	CAF
	Dominica	DMA		Chad	TCD
	Dominican Republic	DOM		Republic of Congo	COG
	Grenada	GRD		Democratic Republic of	COD

For peer review only

				the Congo	
	Guyana	GUY		Equatorial Guinea	GNQ
	Haiti	HTI		Gabon	GAB
	Jamaica	JAM		Gambia	GMB
	Saint Kitts	KNA		Ghana	GHA
	Saint Lucia	LCA		Guinea	GIN
	Saint Vincent	VCT		Guinea-Bissau	GNB
	Suriname	SUR		Liberia	LBR
	Trinidad	TTO		Mali	MLI
**Eastern and southern Africa**	Angola	AGO		Mauritania	MRT
	Botswana	BWA		Niger	NER
	Comoros	COM		Nigeria	NGA
	Eritrea	ERI		Sao Tome and Principe	STP
	Swaziland	SWZ		Senegal	SEN
	Ethiopia	ETH		Sierra Leone	SLE
	Kenya	KEN		Togo	TGO
	Lesotho	LSO	**Western and central Europe and North America**	Andorra	AND
	Madagascar	MDG		Austria	AUT
	Malawi	MWI		Belgium	BEL
	Mauritius	MUS		Bulgaria	BGR
	Mozambique	MOZ		Canada	CAN
	Namibia	NAM		Croatia	HRV
	Rwanda	RWA		Cyprus	CYP
	Seychelles	SYC		Czech Republic	CZE
	South Africa	ZAF		Denmark	DNK

For peer review only

	South Sudan	SSD		Estonia	EST
	Uganda	UGA		Finland	FIN
	Tanzania	TZA		France	FRA
	Zambia	ZMB		Germany	DEU
	Zimbabwe	ZWE		Greece	GRC
**Eastern Europe and central Asia**	Albania	ALB		Hungary	HUN
	Armenia	ARM		Iceland	ISL
	Azerbaijan	AZE		Ireland	IRL
	Belarus	BLR		Israel	ISR
	Bosnia and Herzegovina	BIH		Italy	ITA
	Georgia	GEO		Latvia	LVA
	Kazakhstan	KAZ		Lithuania	LTU
	Kyrgyzstan	KGZ		Luxembourg	LUX
	Montenegro	MNE		Netherlands	NLD
	Moldova	MDA		Norway	NOR
	Russia	RUS		Poland	POL
	Tajikistan	TJK		Portugal	PRT
	Micronesia (Federated States of)	MKD		Romania	ROU
	Turkmenistan	TKM		Serbia	SRB
	Ukraine	UKR		Slovakia	SVK
	Uzbekistan	UZB		Slovenia	SVN
				Spain	ESP
				Sweden	SWE
				Switzerland	CHE

For peer review only

				Turkey	TUR
				UK	GBR
				USA	USA

For peer review only

PRISMA-P (preferred reporting items for systematic review and meta-analysis protocols) 2015 checklist: recommended items to address in a systematic review protocol

Section and topic	Item No	Checklist item	Page number
**Administrative information**			
Title:			
Identification	1a	Identify the report as a protocol of a systematic review	1
Update	1b	If the protocol is for an update of a previous systematic review, identify as such	NA
Registration	2	If registered, provide the name of the registry (such as PROSPERO) and registration number	2 & 5
Authors:			
Contact	3a	Provide name, institutional affiliation, e-mail address of all protocol authors; provide physical mailing address of corresponding author	1
Contributions	3b	Describe contributions of protocol authors and identify the guarantor of the review	12
Amendments	4	If the protocol represents an amendment of a previously completed or published protocol, identify as such and list changes; otherwise, state plan for documenting important protocol amendments	NA
Support:			
Sources	5a	Indicate sources of financial or other support for the review	12
Sponsor	5b	Provide name for the review funder and/or sponsor	12
Role of sponsor or funder	5c	Describe roles of funder(s), sponsor(s), and/or institution(s), if any, in developing the protocol	NA
**Introduction**			
Rationale	6	Describe the rationale for the review in the context of what is already known	4 & 5
Objectives	7	Provide an explicit statement of the question(s) the review will address with reference to participants, interventions, comparators, and outcomes (PICO)	5
**Methods**			
Eligibility criteria	8	Specify the study characteristics (such as PICO, study design, setting, time frame) and report characteristics (such as years considered, language, publication status) to be used as criteria for eligibility for the review	5 & 6
Information sources	9	Describe all intended information sources (such as electronic databases, contact with study authors, trial registers or other grey literature sources) with planned dates of coverage	6 & 9
Search strategy	10	Present draft of search strategy to be used for at least one electronic database, including planned limits, such that it could be repeated	6

Study records:			
Data management	11a	Describe the mechanism(s) that will be used to manage records and data throughout the review	6
Selection process	11b	State the process that will be used for selecting studies (such as two independent reviewers) through each phase of the review (that is, screening, eligibility and inclusion in meta-analysis)	7 & 8
Data collection process	11c	Describe planned method of extracting data from reports (such as piloting forms, done independently, in duplicate), any processes for obtaining and confirming data from investigators	8
Data items	12	List and define all variables for which data will be sought (such as PICO items, funding sources), any pre-planned data assumptions and simplifications	8 & 14
Outcomes and prioritization	13	List and define all outcomes for which data will be sought, including prioritization of main and additional outcomes, with rationale	5 & 6
Risk of bias in individual studies	14	Describe anticipated methods for assessing risk of bias of individual studies, including whether this will be done at the outcome or study level, or both; state how this information will be used in data synthesis	9
Data synthesis	15a	Describe criteria under which study data will be quantitatively synthesised	5 & 6
	15b	If data are appropriate for quantitative synthesis, describe planned summary measures, methods of handling data and methods of combining data from studies, including any planned exploration of consistency (such as I^2, Kendall's τ)	8
	15c	Describe any proposed additional analyses (such as sensitivity or subgroup analyses, meta-regression)	8 & 9
	15d	If quantitative synthesis is not appropriate, describe the type of summary planned	NA
Meta-bias(es)	16	Specify any planned assessment of meta-bias(es) (such as publication bias across studies, selective reporting within studies)	9
Confidence in cumulative evidence	17	Describe how the strength of the body of evidence will be assessed (such as GRADE)	NA

From: Shamseer L, Moher D, Clarke M, Ghersi D, Liberati A, Petticrew M, Shekelle P, Stewart L, PRISMA-P Group. Preferred reporting items for systematic review and meta-analysis protocols (PRISMA-P) 2015: elaboration and explanation. BMJ. 2015 Jan 2;349(jan02 1):g7647.
